# Binuclear Lanthanide Complexes as Magnetic Resonance and Optical Imaging Probes for Redox Sensing

**DOI:** 10.1002/chem.202404748

**Published:** 2025-05-03

**Authors:** Charlie H. Simms, Daniel Kovacs, Lina Hacker, Euan T. Sarson, Daria Sokolova, Kirsten E. Christensen, Alexandr Khrapichev, Louise A. W. Martin, Kylie Vincent, Stuart J. Conway, Ester M. Hammond, Matthew J. Langton, Stephen Faulkner

**Affiliations:** ^1^ Department of Chemistry Chemistry Research Laboratory University of Oxford Mansfield Road Oxford OX1 3TA UK; ^2^ Department of Oncology University of Oxford Oxford OX3 7DQ UK; ^3^ Department of Chemistry Inorganic Chemistry Laboratory University of Oxford South Parks Road Oxford OX1 3QR UK; ^4^ Department of Chemistry and Biochemistry University of California, Los Angeles 607 Charles E. Young Drive East, Box 951569 Los Angeles CA 90095–1569 USA

**Keywords:** luminescence, magnetic resonance, optical, redox, sensing

## Abstract

We report a family of lanthanide(III) complexes that act as redox probes via both magnetic resonance (MR) and luminescence outputs. The ligands are functionalized with nitro, azobenzene and azide groups which are reduced to a common aniline product, and each responds to both chemical and biocatalytic reductive conditions at different cathodic onset potentials. By judicious choice of complexed Ln(III), the probes can be optimized either for use in MR imaging (Ln = Gd), or as highly efficient turn‐on luminescence probes (Ln = Tb). The Tb(III) analogues are essentially nonemissive, until reductive generation of the aniline affords a complex which when excited by visible light (488 nm) emits green light with a quantum yield of 45% and millisecond long luminescent lifetimes (ms). The tunable redox response and imaging modalities of these versatile complexes have the potential to open up new approaches to redox sensing, such as the imaging of hypoxic environments in biology.

## Graphical abstract

Binuclear lanthanide complexes bearing nitrophenol, azophenol and azide chromophores can be reduced to the amine, giving rise to dramatic changes in the luminescence behavior. Such reductions can be achieved using enzymes as well as by chemical means. The tunable redox response and imaging modalities of these versatile complexes have the potential to open up new approaches to redox sensing.

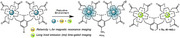

## 1 Introduction

Hypoxia is a condition defined by low levels of oxygen in biological tissues.^[^ ^1^ ^]^ While hypoxia can be induced by prolonged exposure at high altitude,^[^ ^2^ ^]^ it is more commonly a result of underlying disease, such as chronic obstructive pulmonary disease (COPD), diabetes and cancer.^[^ ^3^ ^]^ In cancer, the uncontrollable and rapid growth of malignant cells and inefficient tumour vasculature, results in inefficient blood supply and uneven oxygen distribution.^[^ ^4^ ^]^ Tumours which experience extreme levels of hypoxia (radiobiological hypoxia, <0.1% O_2_) are particularly resistant to radiotherapy, requiring up to three times the radiation dose to achieve cell death.^[^ ^5^, ^6^ ^]^ Therefore, the detection and imaging of hypoxic tissue can lead to improved understanding of the role of hypoxia in cancer, ultimately leading to superior therapeutic strategies and improved patient prognosis.^[^ ^7^ ^]^

Hypoxia presents a unique, highly reductive biochemical environment, due to both reduced oxygen levels and the overexpression of a wide range of reductase enzymes^[^ ^8^ ^]^ including nitro reductases,^[^ ^9^ ^]^ azoreductases,^[^ ^10^ ^]^ and cytochrome P450 reductases.^[^ ^11^ ^]^ These enzymes can catalyse the oxygen‐dependent multi‐electron reduction of common classes of compounds, including nitroaromatics, azobenzenes, and azides,^[^ ^12^ ^]^ ultimately through to a common aniline derivative. The transformation of these functional groups has been successfully integrated into the design of organic fluorescent probes for imaging hypoxic environments.^[^ ^12^, ^13^ ^]^ However, the mechanisms and physiological effects of hypoxia are complex, and further investigation is required to fully understand the generation and impact of hypoxic environments in biology.

To this end, it would be desirable to possess molecular probes that respond to a broad variety of reductive stimuli, and which allow the biochemistry of hypoxia to be interrogated in detail. For instance, hydrogen sulfide is a key endogenous gasotransmitter and a biomarker for hypoxia. Under hypoxic conditions, hydrogen sulfide can accumulate and inhibit mitochondrial respiration,^[^ ^14^ ^]^ and as such, there is a pressing need for chemical probes for monitoring the presence of hydrogen sulfide. The reduction of aryl azides has been previously exploited to this effect.^[^ ^15^ ^]^

Lanthanide complexes, particularly those based on gadolinium(III), are widely employed as MR imaging agents,^[^ ^16^ ^]^ including “smart” systems which target specific tissue types or that exhibit a response modulated by external biochemical stimuli.^[^ ^17^ ^]^ Lanthanide(III) cations are also intrinsically luminescent and exhibit long excited‐state lifetimes and are promising tools for optical imaging applications.^[^ ^18^ ^]^ A common strategy used to overcome the weak emission of lanthanide(III) metals due to the forbidden f‐f transitions is through incorporation of a chromophore into the ligand structure (the “antenna” effect), which allows the lanthanide excited state to be indirectly populated through energy transfer from the chromophore's excited state.^[^ ^19^ ^]^ Modification of the antenna, through both binding and in situ reactions, has been exploited to develop several chemical probes to sense biologically relevant species including anions, cations, reactive oxygen species (ROS).^[^ ^20^ ^]^ Furthermore, the sharp emission bands and wide variety of luminescent lifetimes associated with lanthanide complexes lend themselves to multiplexing and parallel processing.^[^ ^20^, ^21^ ^]^

Recently, Allen and coworkers have developed a divalent europium system for imaging hypoxic environments, which utilises the Eu(II)/Eu(III) redox couple to exploit a “turn‐off” MR response in the presence of a hypoxic environment.^[^ ^22^ ^]^ However, Eu(II) compounds are unstable to air and preparation of the samples for treatment is challenging in a clinical setting. An alternative approach to imaging reductive environments is to exploit redox‐sensitive coordinating ligands, in which chemical transformation of the ligand modulates the sensitization of the lanthanide. This approach has recently been demonstrated in the context of an enzymatic‐triggered turn‐on luminescence response.^[^ ^23^ ^]^

**Figure 1:**
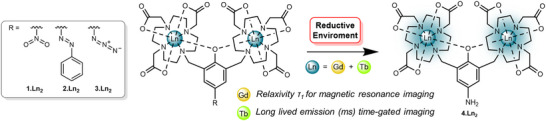
Redox‐activated, luminescent lanthanide probes based on the reduction of nitro, azo, and azide groups to a corresponding aniline. The Gd(III) complexes provide a magnetic resonance (MR) read‐out of reductive conditions, whilst the reduction of the Tb(III) species affords turn‐on luminescence sensors with long‐lived green‐light emission.

Herein, we report unprecedented dual‐modal redox‐responsive bimetallic lanthanide (III) probes able to image reductive environments through turn‐on luminescence and MR response (Figure 1). We prepared a family of nitro, azido, and azo‐based redox‐responsive ligands, which upon reduction generate a common aniline product. In the case of the Tb(III) complexes, this yields an unprecedented turn‐on probe for reductive environments, in which generation of the aniline derivative sensitises the Tb(III) luminescence. Preparation of the analogous redox‐triggered Gd(III) complexes allows access to MR responsive variants.

## 2 Results and Discussion

### 2.1 Design and Synthesis

**Figure 2:**
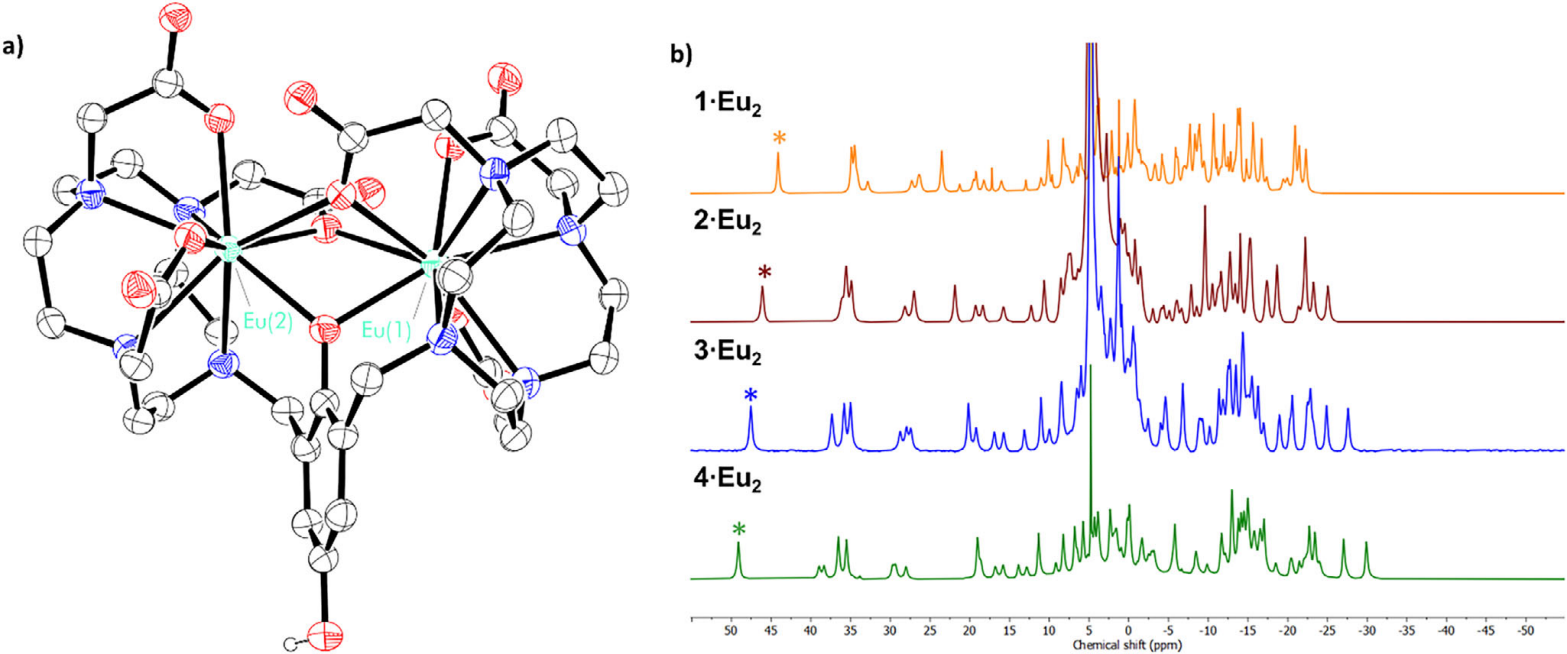
a) **5·Eu_2_**. All H atoms (excluding OH protons) and noncoordinated solvent molecules have been omitted for clarity b) ^1^H NMR of Eu(III) analogues, **1·Eu_2_**, **2·Eu_2_**, **3·Eu_2_** and **4·Eu_2_** (400 MHz, 298 K). * represents the most shifted axial DO3A N‐CH_2_CH_2_‐N protons in the macrocycle.

Previous studies on related kinetically stable phenolate bridged binuclear lanthanide compounds have shown that such systems are emissive, in contrast to their nonemissive mononuclear counterparts.^[^ ^24^ ^]^ A crystal structure of a related compound, **5·Eu_2_** (Figure 2a) reveals the compact nature of the complex, in which the two lanthanide metal centres are facing each other, shielded from the bulk environment. We anticipated that such a system should be amenable to generation of a reduction‐sensitive system, by judicious choice of bridging phenolate ligand. Therefore, we targeted a family of compounds in which the phenolate was functionalised with redox active groups in the *para* position (Figure 1).^[^ ^25^ ^]^

**Scheme 1:**
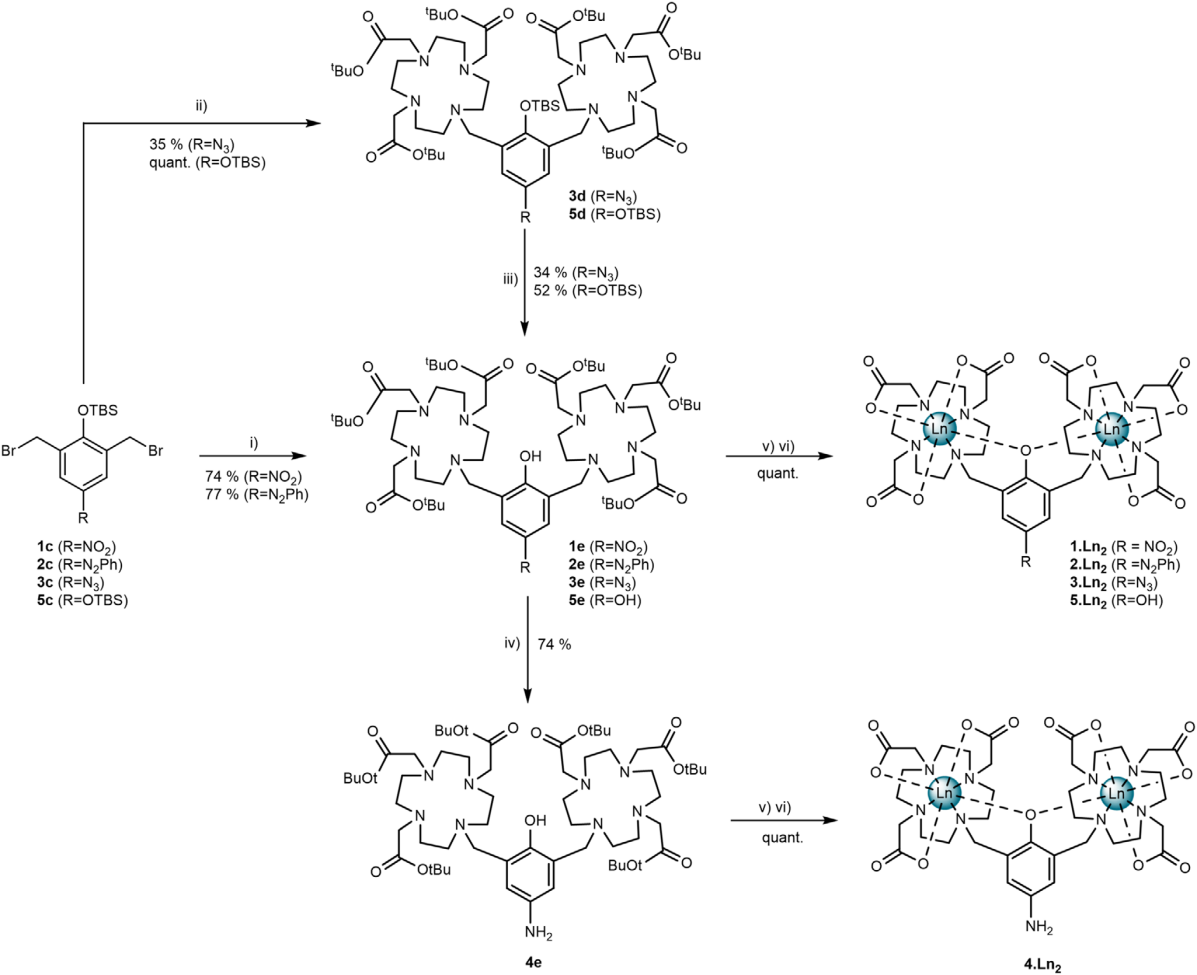
Synthesis of probes **1–5·Ln_2_**. Conditions: i)/ii) DO3A^t^Bu, Na_2_CO_3_, MeCN, 60 °C, 48 hours iii) TASF, DMF, rt, 18 hours iv) 10% Pd on carbon, H_2_, EtOH, rt, 5 days v) TFA: DCM (1:1), r.t., 18 hours vi) Ln(OTf)_3_, NaOH(aq), H_2_O, 50 °C, 24 hours.

Motivated by the redox sensitivity of nitroaromatics, azobenzene, and azide groups, all of which are reduced to a common aniline, we prepared three redox‐responsive ligands **1e‐3e** and two control ligands **4e** and **5e** (Scheme 1). These were prepared in 5–6 steps from commercially available precursors, following a common procedure in which the phenol derivative was protected with a *tert*‐butyldimethylsilyl (TBS) group, brominated and then reacted with two equivalents of the *tert*‐butyl ester derivative of 1,4,7,10‐tetraazacyclododecane‐1,4,7‐triacetic acid (DO3A). Successive deprotection of the TBS group and *tert*‐butyl protected esters yielded the ligands and subsequent complexation with the corresponding Ln(III) triflate yielded the desired Ln(III) complexes **1·Ln_2_‐ 5·Ln_2_**. The aniline, **4·Ln_2_** could be obtained by reduction of **1e** using palladium on carbon, under a hydrogen atmosphere to give **4e**, then successive deprotection of the *tert*‐butyl esters and finally complexation with the corresponding lanthanide triflate. Alternatively, the lanthanide complexes **1–3·Ln_2_** could be reduced in situ to yield **4·Ln_2_** (Scheme S1). For full synthesis and characterization details, see the Supporting Information.

We prepared the Tb(III) complexes of each ligand for luminescence experiments and the Gd(III) complexes to investigate MR properties. The corresponding Eu(III) complexes were synthesised for additional NMR analysis and characterization of the reduction chemistry

### 2.2 ^1^H NMR

The ^1^H spectra of all four paramagnetic Eu(III) complexes are shown in Figure 2b. Sharp, well‐resolved signals are indicative of a rigid structure where the Eu(III) metal center is strongly coordinated by the ligand.^[^ ^26^ ^]^ The axial proton (labelled as *) is de‐shielded in the presence of increasingly activating groups, providing a convenient handle to monitor reduction of **1–3·Eu_2_** to the aniline **4·Eu_2_** (Figures S27–29).

### 2.3 Optical Properties

Initially, the luminescence properties of the Tb(III) analogues were explored. In comparison to other Ln(III) ions, Tb(III) complexes can be highly luminescent reaching quantum yields of up to 54%.^[^ ^27^ ^]^ Tb(III) emits green light and has long excited state luminescent lifetimes in the millisecond range, and as such it is a promising candidate for microscopy and imaging applications.^[^ ^28^ ^]^

**Figure 3:**
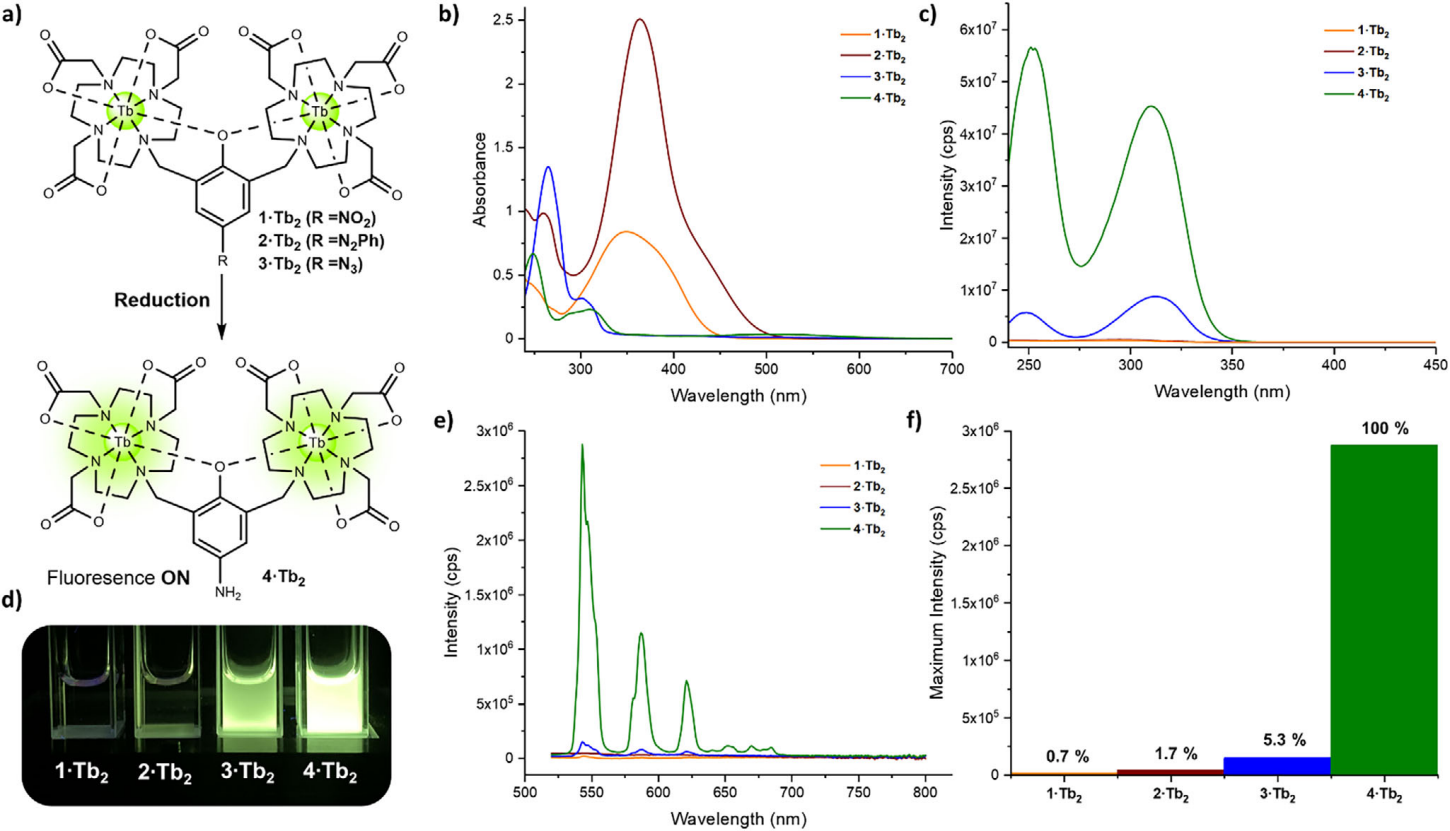
Optical properties of probes **1–4·Tb_2_** a) Schematic showing the reduction of probes **1–3·Tb_2_** to **4·Tb_2_** resulting in a “turn‐on” of long‐lived luminescence b) Absorbance spectra of **1–4·Tb_2_** at 90 mmol in PBS pH 7.4 c) Excitation spectra of **1–4·Tb_2_** were recorded with λ_em_ = 545 nm, emission slits 5 nm, excitation slit 1 nm, integration time 0.5 s d) Visualization of the difference in emission intensity of all probes upon excitation with 254 nm light e) Emission spectra were recorded with λ_ex_ = 488 nm, excitation slits 5 nm, emission slit 1 nm, integration time 0.5 s. f) Comparison of the maximum intensity recorded for each probe upon excitation at 488 nm, where they are represented as a percentage of the maximum intensity of **4·Tb_2_**.

The absorbance and excitation spectra of **1–4·Tb_2_** are shown in Figures 3b and c. Each complex exhibits a distinctive absorption profile dependent on the group present, with the azido and aniline derivatives predominantly absorbing in the UV, with no absorption in the blue region, whilst the electron withdrawing nitro and azo derivatives are comparatively red shifted, exhibiting strong absorbance in the blue region. This trend is reversed in the excitation spectra (Figure 3b), in which **3·Tb_2_** and **4·Tb_2_** act as efficient sensitisers for Tb(III) emission when excited at wavelengths between 240 and 350 nm. Conversely **1·Tb_2_** and **2·Tb_2_** are comparatively dark. The efficiency of the antenna effect, which describes the energy transfer from a chromophore to a lanthanide, is a function of the absorption coefficient, degree of spectral overlap with the lanthanide and the availability of nonradiative decay pathways. Here, **1·Tb_2_** and **2·Tb_2_** have inefficient spectral overlap with the Tb(III) centre, and in the case of **2·Tb_2_** the *E*/*Z* isomerization of the azo ligand provides an efficient nonradiative decay pathway, rendering the ligand a poor sensitizer for Tb(III).^[^ ^29^ ^]^ For means of comparison between all four Tb(III) complexes, the emission was recorded upon direct excitation into the ^7^F_6_ → ^5^D_4_ transition (λ_ex_ = 488 nm) of the Tb(III) metal centre, in order to explore the intrinsic efficiency of the Tb(III) emissive centre and the influence of available nonradiative decay pathways from the ligand framework.

Upon direct excitation into the Tb(III) centre, the aniline complex **4·Tb_2_** afforded bright green emission (Figure 3e). **4·Tb_2_** exhibited a quantum yield (ϕ_lum_) of 45% following excitation at 310 nm. In contrast, the masked probes **1–3·Tb_2_** were weakly emissive and almost completely quenched in the case of **1·Tb_2_** and **2·Tb_2_** (0.7% and 1.7% intensity in comparison to the intensity of the ^5^D_4_→ ^7^F_5_ transition of **4·Tb_2_**, respectively, Figure 3f). The azido derivative **3·Tb_2_** was prone to photoreduction, and prolonged exposure to blue light increased the emission intensity. The marked difference in emission between the highly emissive aniline **4·Tb_2_** complex and the weakly or nonemissive masked derivatives **1–3·Tb_2_** could be observed by the naked eye under 254 nm irradiation from a commercial UV lamp (Figure 3d). The stark difference in emission intensity between the masked probes **1–3·Tb_2_** and the unmasked **4·Tb_2_** revealed that such a system shows significant promise as an off‐on luminescent probe for reductive conditions.

Luminescent lifetimes for each Tb(III) complex were recorded in Phosphate Buffered Saline (PBS) buffered H_2_O (pH 7.4) and D_2_O (pD 7.4 (pH + 0.45)), following direct excitation into the ^5^D_4_→ ^7^F_5_ transition (*λ*_ex_ = 488 nm) of the metal centre (Table 1). A monoexponential decay profile was recorded for complexes **1·Tb_2_**, **3·Tb_2_,** and **4·Tb_2_** in water, whilst **2·Tb_2_** exhibited a biexponential decay profile. This is indicative of rapid quenching of the **2·Tb_2_** excited state, likely mediated by rapid *E*‐*Z* isomerization of the azobenzene fragment.^[^ ^30^ ^]^ Luminescent lifetimes for **1·Tb_2_**, **3·Tb_2_,** and **4·Tb_2_** ranged between 2.2 and 2.4 ms in water, increasing modestly to 2.5–2.8 ms in D_2_O, suggesting a contribution from an outer sphere oscillator effect.^[^ ^31^ ^]^ This is supported by calculation of the number of water molecules directly bound to the metal centre (*q*) using the modified Horrocks equation.^[^ ^32^ ^]^ Values of *q* of ∼0 in each case are indicative of an absence of inner sphere bound water molecules due to the coordinated phenol ligand, thus maximizing the luminescence of the un‐masked probe, **4·Tb_2_**.

**Table 1:** Luminescent lifetimes of **1–4·Tb_2_** in PBS buffered H_2_O (pH 7.4) and D_2_O (pD 7.4 (pH + 0.45)) upon excitation at 488 nm, and the corresponding *q* values (number of bound solvent) as calculated by the modified Horrocks equation^32^ (q_Tb_ = 5(τ_H2O_^−1^ − τ_D2O_^−1^ ‐ 0.06).

Compound	τH_2_O /ms	τD_2_O /ms	q_Tb_
**1·Tb_2_**	2.28	2.85	0.1
**2·Tb_2_**	1.00, 0.20^[^ ^a^ ^]^	0.85	‐
**3·Tb_2_**	2.38	2.54	∼ 0
**4·Tb_2_**	2.28	2.49	∼ 0

^[a]^ **2·Tb_2_** fitted to a biexponential lifetime

### 2.4 Chemical and Electrochemical Reduction

**Figure 4:**
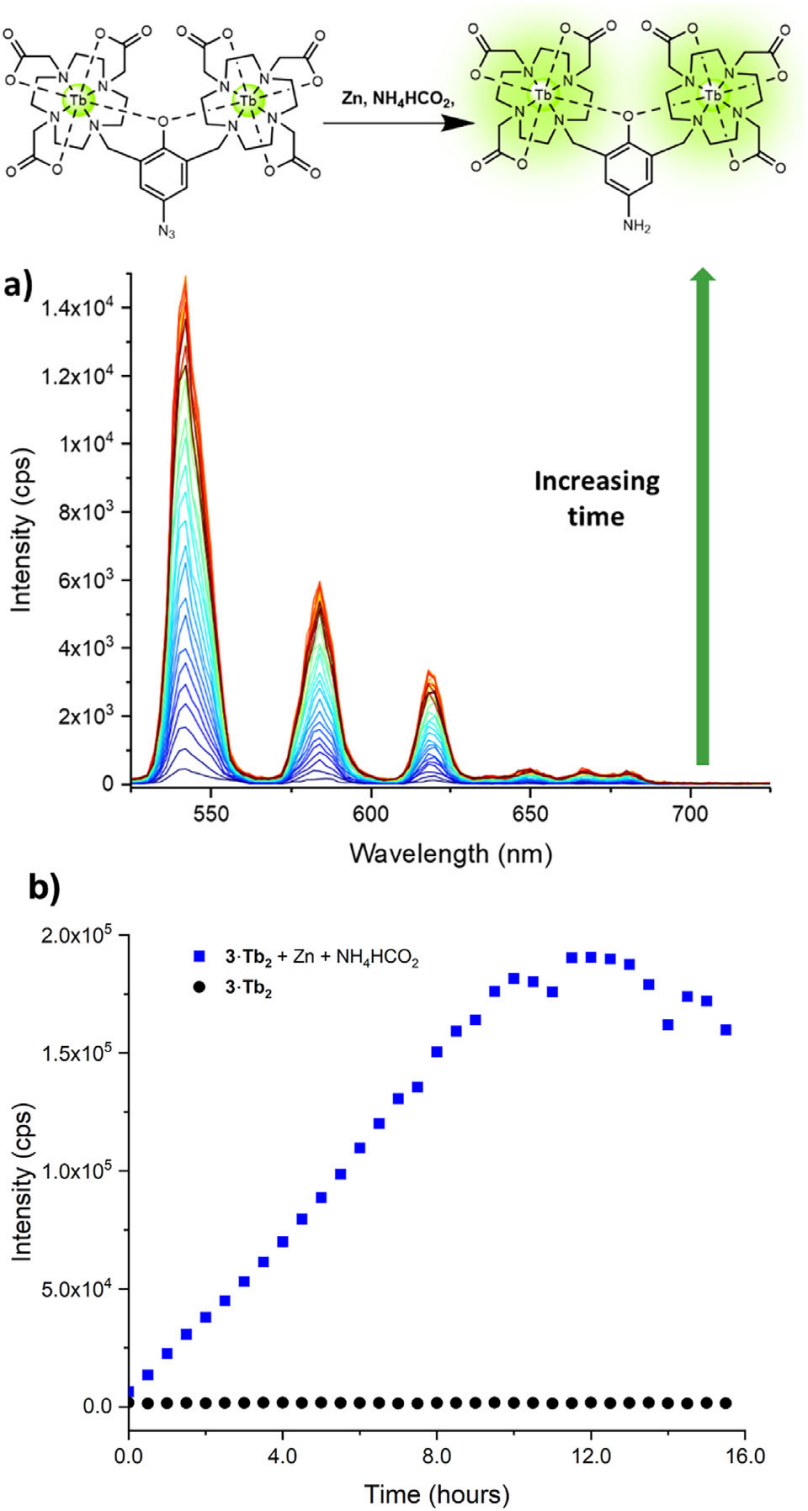
Chemical reduction of **3·Tb_2_** (0.01 M in MES (0.1 M)) to **4·Tb_2_** using Zinc/NH_4_HCO_2_ a) Emission spectra showing the increase in Tb(III) emission over 16 hours b) Total intensity of emission as a function of time (measurement taken every 30 minutes for 16 hours) of **3·Tb_2_** (0.01 M in MES (0.1 M)), without (black) and with Zinc/NH_4_HCO_2_ (blue). *λ*_ex_ = 280 nm, emission slit = 2.5 nm, excitation slit = 2.5 nm, gain = 165, integration time 0.2 ms.

To investigate the “turn‐on” abilities of the Tb(III) probes, complexes **1–3·Tb_2_** were chemically reduced by exposure to zinc and ammonium formate at varying concentrations. The emission intensity was monitored over time following excitation via the ligand at 240 nm. In the presence of ammonium formate and zinc, the Tb(III) emission increased rapidly over time (representative data for the reduction of **3·Tb_2_** is shown in Figures 4a and 4b; data for compounds **1·Tb_2_** and **2·Tb_2_** are given in the appendix (Figures S36–46). In the absence of ammonium formate and zinc, negligible Tb(III) emission was observed for the duration of the experiment with probes **1–3·Tb_2_** (Figures S36, S42, and S46). The reduction of probes **1–3·Tb_2_** all yielded the common aniline species **4·Tb_2_** as the product, as confirmed by ^1^H NMR (Figures S27–29) and HR‐ESI mass spectrometry experiments (See Supporting information). All masked probes **1–3·Tb_2_** were fully reduced to **4·Tb_2_** upon exposure to Zn/NH_4_HCO_2_ within 16 hours.

**Figure 5:**
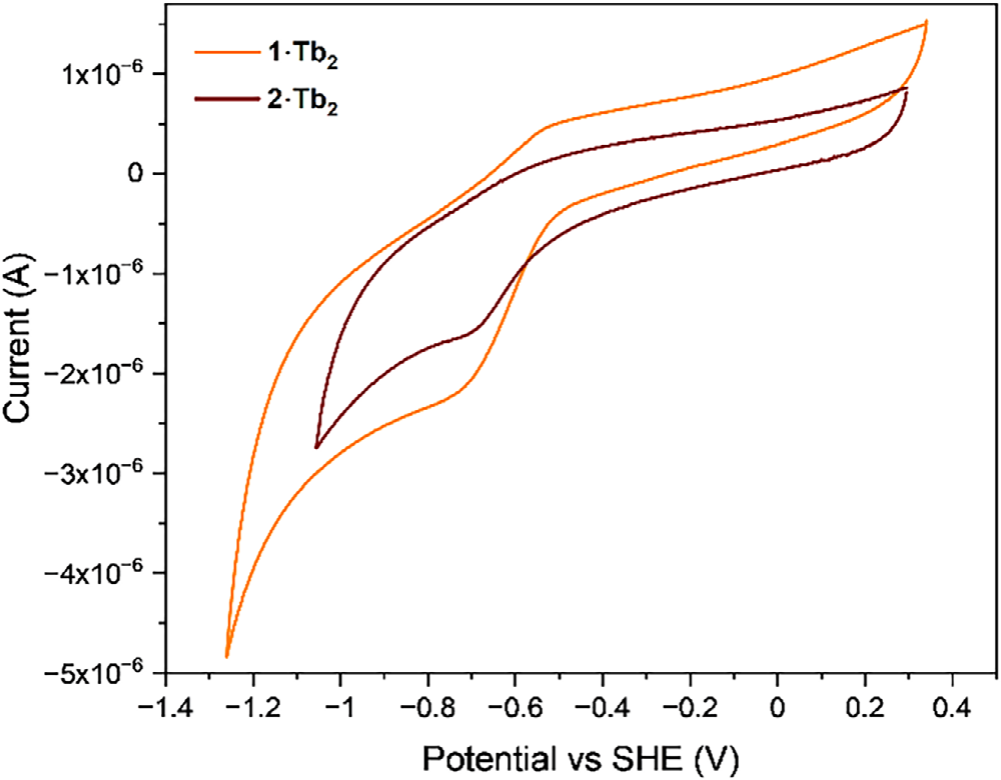
Cyclic voltammograms for **1·Tb_2_** and **2·Tb_2_** at a glassy carbon electrode (1 mM in Tris (50 mM with 50 mM NaCl)) at pH 7.4, scan rate = 0.02 V/s.

The electrochemical reduction of probes **1–3·Tb_2_** was investigated by cyclic voltammetry. The cathodic onset potentials of **1·Tb_2_** and **2·Tb_2_** at pH 7.4 were determined as ‐0.41 and ‐0.39 V (versus SHE), respectively, (Figure 5), consistent with the redox potential of related nitrophenyl and azobenzene compounds.^[^ ^33^ ^]^ The onset potential of **3·Tb_2_** could not be determined (Figure S25). The subtle difference in onset potential of **1·Tb_2_** and **2·Tb_2_** and their pH dependence (Table S2) is promising for the utility of the compounds in investigating a range of redox potentials in solution, given that the redox potential of hypoxic tissues has been approximated to between ‐0.33 and ‐0.44 V (versus SHE).^[^ ^34^ ^]^

### 2.5 Detecting Hydrogen Sulfide in Solution

**Figure 6:**
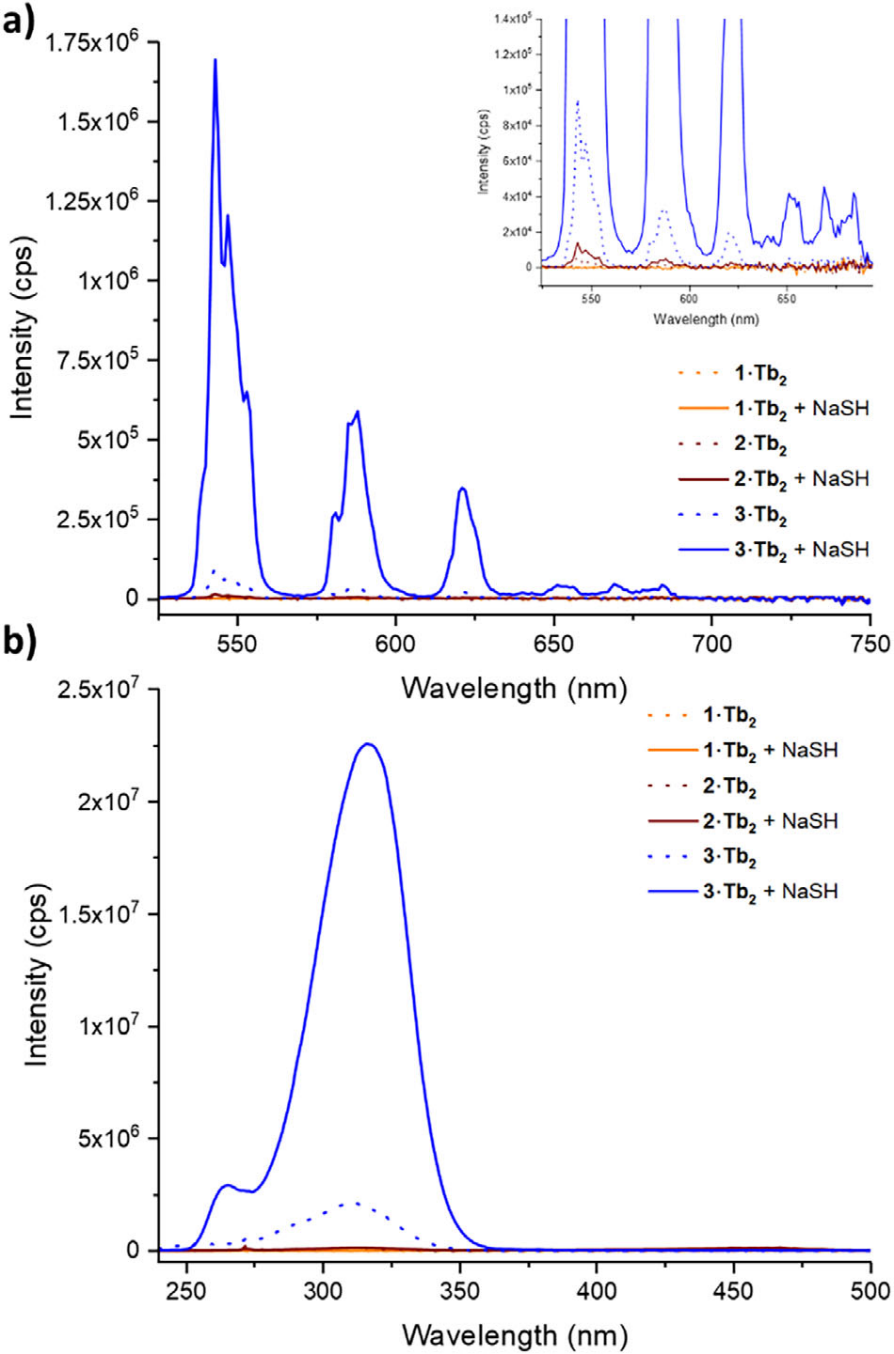
Emission and excitation spectra of redox‐activatable probes **1–3·Tb_2_** (90 µM in PBS at pH 7.4) upon exposure to NaSH (250 µM) a) Emission spectra of **1–3·Tb_2_** before and after exposure to NaSH (2.7 eq.) (*λ*_ex_ = 318 nm, emission slits: 1 nm, excitation slits: 1 nm, integration time 0.5 seconds). b) Excitation spectra of **1–3·Tb_2_** before and after exposure to NaSH (*λ*_em_ = 545 nm, emission slits: 5 nm, excitation slits: 1 nm, integration time 0.5 seconds).

To demonstrate the utility of the probes to detect hydrogen sulfide, a known biomarker of hypoxia, masked probes **1–3·Tb_2_** were exposed to 250 µM NaSH for 10 minutes, in PBS buffer at pH 7.4, and the emission spectra (λ_ex_ = 318 nm) were measured before and after the addition of hydrogen sulfide (Figure 6a). For probes **1·Tb_2_** and **2·Tb_2_** there was no significant change in intensity of Tb(III) emission in the presence of NaSH. In contrast, reduction and turn‐on emission of the analogous aryl‐azide probe **3·Tb_2_** was observed, with a 20‐fold increase in emission intensity in the presence of hydrogen sulfide. Similarly, the excitation spectra (*λ*_em_ = 545 nm, Figure 6b) for **1·Tb_2_** and **2·Tb_2_** were unaffected by addition of hydrogen sulfide, whilst that of **3·Tb_2_** in the presence of NaSH mirrored that of the amino compound **4·Tb_2_** (Figure S53). These results demonstrate that **3·Tb_2_** can be utilized as a visible light probe for the sensing of hydrogen sulfide in solution.

### 2.6 Biocatalytic Reduction

**Figure 7:**
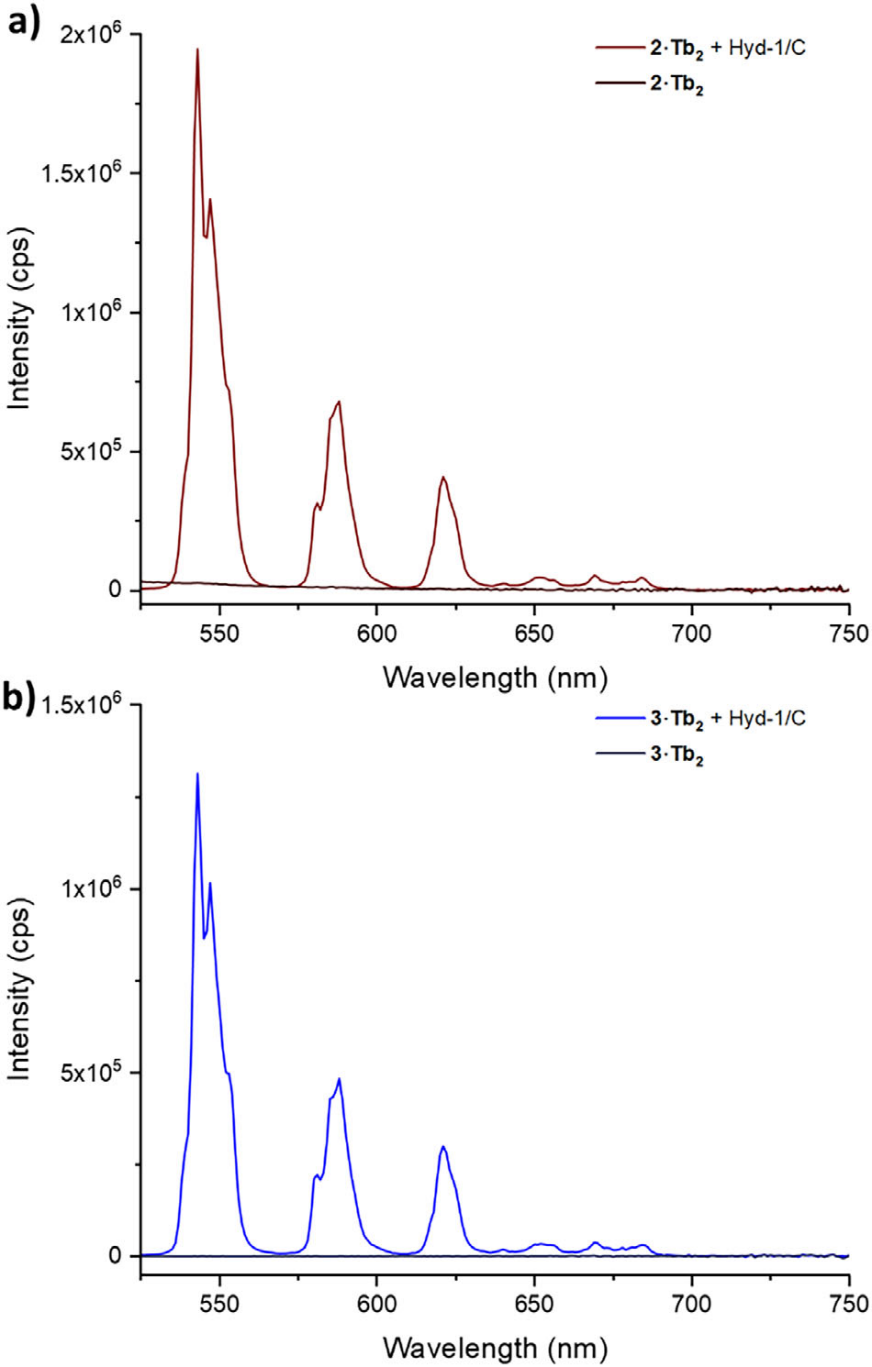
Emission properties of redox‐activatable probes **1–3·Tb_2_** (90 µM in PBS at pH 6.0) upon exposure to Hydrogenase ‐1 on carbon support a) **2·Tb_2_** before and after exposure to Hyd‐1/C b) 3·Tb_2_ before and after exposure to Hyd‐1/C (*λ*_ex_ = 318 nm, emission slits: 3 nm, excitation slits: 1 nm, integration time 0.5 seconds).

To explore the possibility of biocatalytic reduction of the three probes, we exploited a carbon‐immobilised hydrogenase enzyme.^[^ ^35^ ^]^ Nickel‐iron hydrogenase Hyd‐1, adsorbed onto a carbon‐black support (Hyd‐1/C), have previously been shown to facilitate the reduction of a wide range of substrates containing nitro‐aryl groups.^[^ ^33^, ^36^ ^]^ Accordingly, **1–3·Tb_2_** (10 mM) in PBS (50 mM, pH 6.0) were treated with Hyd‐1/C (C:Hyd‑1 = 40:1 mass ratio, 0.53 mg of C, 13 µg of Hyd‐1 per reaction) at pH 6.0 in water (10% DMSO) under 30 mL/minute flow of H_2_ for 24 hours at room temperature (for full experimental details see Supporting Information). Under these conditions only the reduction of **2·Tb_2_** was observed by emission spectroscopy (Figure 7a). Upon increased catalyst loading (1.06 mg of C, 26 µg of Hyd‐1 per reaction) and reaction time (72 hours) the reduction of **3·Tb_2_** could be achieved (Figure 7b). Notably, **1·Tb_2_** was not reduced, even under extended reaction times (72 hours) and increased enzyme loading (1.06 mg of C, 26 µg of Hyd‐1 per reaction) (Figure S55). Previous work has found that similar reductions of aryl‐nitro derivatives with a *para*‐phenolate moiety using the Hyd‐1/C reduction system are also inhibited, which may account for the lack of reduction of **1·Tb_2_** observed here. Work to understand this mechanism that underpins this inhibition is ongoing.

The orthogonality in the reduction of the three probes, namely the selective turn‐on behavior of only complex **3·Tb_2_** in the presence of hydrogen sulfide, and selective turn‐on behavior of **2·Tb_2_** to biocatalytic reduction under low enzyme loadings and short reaction times, points to the possibility of selective imaging of various biological environments characterized by different reducing conditions. Preliminary cellular uptake studies with **1–4·Tb_2_** into colorectal cell line HCT116 (Figure S54), suggested poor uptake of these systems, likely due to the neutrality and polarity of the lanthanide compounds.^[^ ^37^ ^]^ Work is currently ongoing to improve the cell permeability of the lanthanide complexes.

### 2.7 MR and Relaxivity

**Figure 8:**
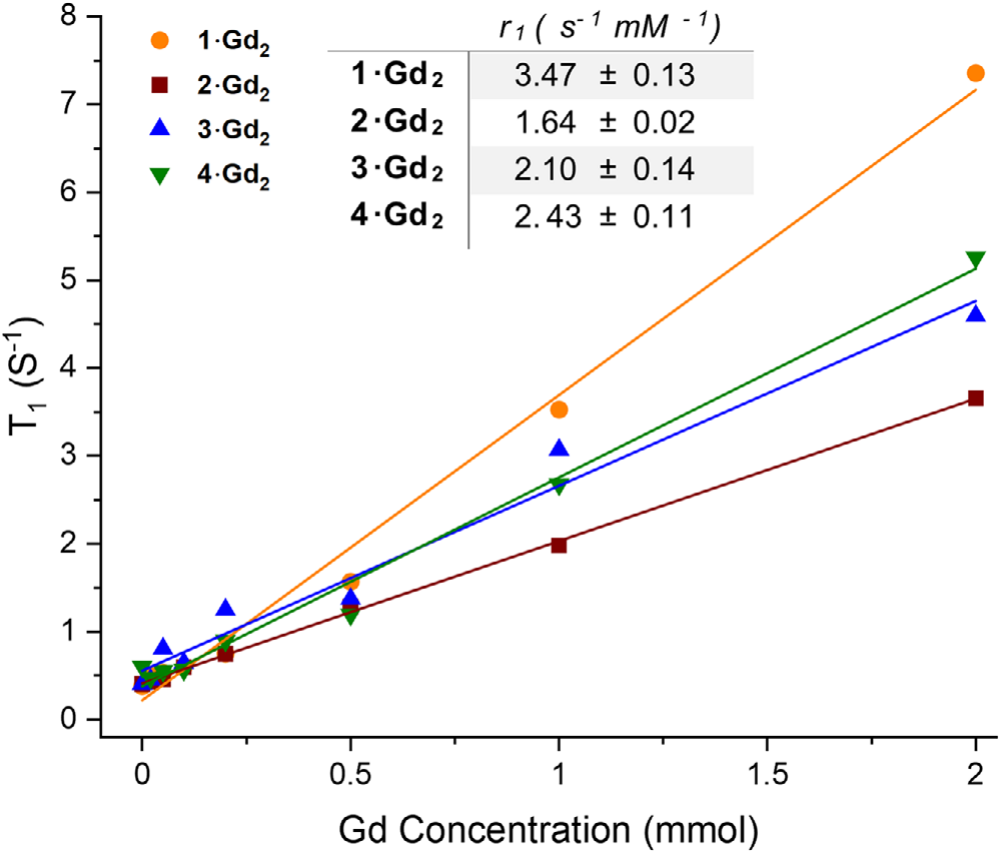
T_1_ of Gd(III) analogues **1·Gd_2_**, **2·Gd_2_**, **3·Gd_2_**, and **4·Gd_2_** as a function of Gd(III) concentration.

To explore their potential as redox‐sensitive MR probes, we investigated the longitudinal relaxivity (*r*_1_) of complexes **1–4·Gd_2_** in phosphate buffered saline (PBS, pH 7.4). This is quantified by the variation of the water‐proton relaxation rate (*T*_1_^−1^) normalised to the concentration of the paramagnetic complex in solution.^[^ ^38^ ^]^ A linear response was observed for all four probes at 7 T (Figure 8), indicating that each complex is stable in solution, and that *r*_1_ is not influenced by the local environment. Furthermore, longitudinal relaxivity of the nitro derivative **1·Gd_2_** (3.47 s^−1^) is comparable to the clinically available Gd‐DOTA which has a *r*_1_ of 2.8 s^−1^ at 7 T.^[^ ^39^ ^]^

The amino compound, **4·Gd_2_** (2.43 s^−1^), exhibits a decrease in relaxivity compared to **1·Gd_2_**: this system therefore, acts as a turn‐off MRI sensor upon reduction of **1·Gd_2_** to **4·Gd_2_**. In contrast, the reduction of the azo complex **2·Gd_2_** (1.64 s^−1^) to **4·Gd_2_** leads to an increase in relaxivity, and hence acts as a turn‐on MR probe.

## 3 Conclusion

In summary, we have developed a family of redox‐activatable probes **1–3·Ln_2_**, in which a selective response to various reductive environments is transduced into either an off‐on, or on‐off, MR response for the Gd(III) complexes, or an off‐on luminescence resonance with high emission quantum yield for the analogous Tb(III) complexes. The reduced complex **4·Tb_2_** displays particularly favourable properties for imaging: it can be excited with visible light (488 nm), emits strongly in the green region, and possesses long‐lived luminescence lifetimes (ms). In contrast, **1–3·Tb_2_** are essentially dark under the same conditions. This allows for a significant turn‐on luminescent response upon reduction with high contrast, using wavelengths of light that are biocompatible. Electrochemical reduction was successful for **1–2·Tb_2_,** revealing that the complexes possess reduction potentials within the range of previously measured for hypoxic cells. The complexes are also responsive to enzymatic reduction, and importantly, **3·Tb_2_** optically detect hydrogen sulfide in solution, a known biomarker for hypoxia. Overall, these results demonstrate the unique fundamental properties of lanthanide complexes for redox imaging applications. The reductive orthogonality of the complexes suggests that such systems could allow for the development of probes to establish the precise nature of reductive stress, and we anticipate that such probes will be key to understanding the mechanisms, diagnosis and treatment of hypoxia and reductive biological environments more broadly.

## 4 Experimental Section

### General procedure for the synthesis of the bimetallic lanthanide complexes

*Tert*‐butyl ester derivative of 1,4,7,10‐tetraazacyclododecane‐1,4,7‐triacetic acid (DO3A, 1.75 eq., ∼2 mmol) was added to the TBS‐protected 2,6‐dibromomethyl derivative **1–3c** or **5c** (1 eq.) in MeCN in the presence of sodium carbonate (3 eq.) and stirred at 60 °C for 48 hours. The resulting suspension was filtered, and the filtrate was concentrated under reduced pressure. The residue was redissolved in a minimum amount of DCM:acetone (1:1) mixture and then purified by silica gel flash‐column chromatography and eluted with DCM:acetone:iPrOH:MeOH. The product was redissolved in chloroform and was filtered through a membrane filter (Nylon, 0.45 µm pore size) and evaporated to dryness to afford the pure product. Silane deprotection of **3d** and **5d** was conducted by stirring the ligand in THF with 2 eq. tetrabutylammonium fluoride for 16 hours, prior to purification by silica gel flash‐column chromatography (DCM/MeOH eluent). Deprotection of the *t*‐Bu ester derivatives **1**–**3e** and **5e** was achieved by dissolving the ligand in DCM, followed by the dropwise addition of 1:1 trifluoroacetic acid / DCM (v/v) and stirred for 16 hours. The crude was evaporated to dryness and precipitated from a methanol solution with ether. Metal complexation was achieved by stirring the appropriate deprotected ligand **1**–**5e** (0.05 M) with 2.4 eq. of the Ln‐trifalte salt in water at 50 °C for 24 hours. The resulting solution was purified by dialysis using Float‐A‐Lyzer G2 dialysis tubes (500, 1000 MWCO) equipped with regenerated cellulose for 3 days. Full synthetic procedures and characterization data are available in the supporting information.

### Characterization, spectroscopy, and cyclic voltammetry

Steady‐state excitation and emission spectra were recorded on a Horiba Jobin Yvon Fluorolog 3–12 Fluorometer equipped with a Hamamatsu R928 detector and a double‐grating emission monochromator. S1/R1 response was used throughout as luminescence output. Emission spectra were recorded by exciting samples **1–4·Tb_2_** (90 µM in 1X PBS buffered MilliQ Water) at 488 nm, with a slit width of 23 nm, and recording the emission between 520–800 nm with a band pass of 2 nm and 0.5 seconds integration time. A 2″ square unmounted longpass 400 nm filter (FGL400S) fabricated using a 2 mm thick Schott colored glass from Thor labs was used while recording steady‐state emission. Time‐resolved lifetime measurements were made on Fluorolog 3–12 for **1–4·Tb_2_** (90 µM). UV‐Vis spectra were recorded on a Jasco V‐770 UV‐Visible/NIR Spectrophotometer equipped with Peltier temperature controller and stirrer using quartz cuvettes of 1 cm path length. Mass spectra were carried out on a Waters BioAccord LC‐MS system; flow injection analysis was performed on an ACQUITY I‐Class PLUS UPLC System (Waters, Millford, MA, USA) coupled to an AQUITY RDa mass spectrometer (Waters, Milford, MA, USA) equipped with an ESI probe, in positive ion mode. NMR spectra were obtained using a Bruker Avance III HD nanobay NMR equipped with a 9.4 T magnet (^1^H 400.2 MHz, ^19^F 376.5 MHz, 13C 100.6 MHz), Bruker Avance NMR equipped with a 11.75 T magnet and a ^13^C detect cryoprobe (^1^H 500.3 MHz, 13C 125.8 MHz) and Bruker NEO 600 with broadband helium cryoprobe (1H 600.4 MHz, 13C 151.0 MHz). HPLC analysis was conducted on a DiscoveryCyano column [5 µm, 4.6 × 250 mm] with guard column, [98:2 H_2_O: MeCN to 0:100 H_2_O: MeCN at 1 mL/minute].

Cyclic voltammetry was conducted using a small‐volume electrochemical cell consisting of a polyether ether ketone (PEEK) cylinder containing a working electrode of glassy carbon (1 mm diameter), a counter electrode of graphite, and a leak‐free Ag/AgCl reference electrode (LF‐2–45 model from Alvatek Ltd). The cell was set up under a N2 atmosphere in a glove box (O_2_ < 2 ppm), using 400 µL of a 1 mM solution of complex dissolved in degassed H_2_O containing 50 mM Tris and 50 mM NaCl at pH 7.4 or 100 mM MES and 50 mM NaCl at pH 6.0. The air‐tight electrochemical cell was then removed from the glove box and connected to an AutoLab 128 N potentiostat (Metrohm) controlled by Nova 2.1.7 software. Cyclic voltammograms were run with step potential = ‐0.00244 V and scan rate = 0.02 V/s, with 3 cycles recorded for each. The Ag/AgCl reference electrode was calibrated to versus SHE by measuring the cyclic voltammogram of FcMeOH (0.1 mM in 4:1 buffer:EtOH) and comparing to an E_1/2_ value of + 420 mV versus SHE for FcMeOH.

Relaxivity determination by MRI was conducted using an Agilent 7 T scanner equipped with a DirectDrive console and 400 mT/m imaging gradients (Varian, UK). The RF coil employed was a 72 mm i.d. birdcage resonator (Rapid Biomedical). T1 measurements were carried out using an inversion recovery spin‐echo sequence with the following parameters: slice thickness of 1 mm, field of view (FoV) of 72 × 72 mm, matrix size of 128 × 128, four averages, TR/TE of 10 s/8 ms, and 12 inversion times (Ti) ranging from 0.01 to 6.0 seconds (exponentially spaced). T2 measurements were performed using a spin‐echo sequence with the same single‐slice sequence as the T1 measurements: slice thickness of 1 mm, FoV of 72 × 72 mm, matrix size of 128 × 128, four averages, TR of 10 s, and 12 echo times (TE) ranging from 8 to 300 ms (exponentially spaced). All samples were placed in 1 ml syringes (Terumo) in a custom‐made 3D‐printed phantom holder enabling reproducible measurements. Acquired data was analysed using Matlab (version R2022a).

## Conflict of Interests

The authors declare no conflict of interest.

## Supporting information



Supporting Information

## Data Availability

The data that support the findings of this study are available in the supplementary material of this article.
