# Supplementary material for: Binuclear Lanthanide Complexes as Magnetic Resonance and Optical Imaging Probes for Redox Sensing
Source: Chemistry. 2025 May 3;31(30):e202404748. doi: 10.1002/chem.202404748 (PMC12117176; doi:10.1002/chem.202404748)
Supplement: Supplementary file 1 — Supporting Information [file CHEM-31-e202404748-s001.pdf]

Supporting Information for

Binuclear lanthanide complexes as magnetic resonance and optical imaging probes for redox sensing

Charlie H. Simms,[a†] Daniel Kovacs,[a†] Lina Hacker,[b] Euan T. Sarson,[a] Daria Sokolova,[c] Kirsten E. Christensen,[a] Alexandr Khrapichev,[b] Louise A. W. Martin[b], Kylie Vincent,[a] Stuart J Conway,[d] Ester M Hammond,*[b] Matthew J Langton*[a] and Stephen Faulkner*[a]

[a] Department of Chemistry, Chemistry Research Laboratory, University of Oxford, Mansfield Road, Oxford, OX1 3TA, United Kingdom;

[b] Department of Oncology, University of Oxford, Oxford, OX3 7DQ, United Kingdom

[c] Department of Chemistry, Inorganic Chemistry Laboratory, University of Oxford, South Parks Road, Oxford, OX1 3QR, United Kingdom

[d] Department of Chemistry and Biochemistry, UCLA, 607 Charles E. Young Drive East, Box 951569, Los Angeles, CA 90095-1569, United States

[†]both authors contributed equally

Contents

Abbreviations: 5
Synthesis and Characterisation of compounds: 6
- **General procedure for TBS protection** 7
- **General procedure for radical dibromination** 9
- **Procedures for the protected macrocycles** 11
- **General procedure for tert-butyl ester deprotection** 15
- **General procedure for obtaining the binuclear complexes** 17
- **^{1}H NMR spectra of Ln(III) complexes:** 20
- **HPLC spectra of Ln(III) complexes:** 24
- **Optical Properties** 27
  - **Steady State fluorescence** 27
  - **Lifetimes** 28
  - **Quantum Yields** 28
  - **Triplet Energies** 30
- **X-ray Crystal Structures** 31

Relaxivity 34
Cyclic Voltammetry (CV) 35
Chemical reduction of the binuclear Ln(III) probes 41
- **Reduction procedure for analysis by ^{1}H NMR:** 41
- **Reduction procedure for analysis by luminescence (plate reader Asssay):** 44
  - **1·Tb_2** 46
  - **2·Tb_2** 48
  - **3·Tb_2** 49

Biological reduction of the binuclear Ln(III) probes 52
  - **NaHS Reduction** 52

Cell Studies 54
Biocatalytic Reduction 55
^{1}H and ^{13}C spectra of compounds 57

General Methods and Reagents:

Unless stated otherwise, experiments were performed at 25 °C using reagents and solvents purchased commercially and used without further purification. Anhydrous solvents were acquired by passing them through an MBraun MPSP-800 column followed by degassing with nitrogen. Triethylamine was distilled from and stored over potassium hydroxide. Deionised, microfiltered water was obtained from a Milli-Q™ Millipore machine. Merck silica gel 60 under nitrogen pressure was used for silica gel flash column chromatography. Thin layer chromatography was performed on silica-coated (60G F254) aluminium plates from Merck and aluminium oxide coated with 254 nm fluorescent indicator aluminium plates from Merck. Samples were visualized by UV-light (254 and 365 nm) and/or using permanganate stain. Solvent systems containing a mixture of solvents are reported as a ratio by volume of each solvent.

Float-A-Lyzer® G2 dialysis tubes (500, 1000 MWCO) equipped with regenerated cellulose were purchased from Spectrum and used to purify the lanthanide complexes. The dialysis tube was activated by 10 % ethanol or isopropanol solution followed by MilliQ type 1 deionised water before being used. The corresponding complexes were dissolved in water and transferred into a dialysis tube. The dialysis tube was placed in a 2.5 L-beaker filled with MilliQ type 1 deionised water. The dialysis lasts for at least two days under stirring and the deionised water was replaced with fresh deionised water more than three times during dialysis.

Characterisation Information:

Mass spectra were carried out on a Waters BioAccord LC-MS system; flow injection analysis was performed on an ACQUITY I-Class PLUS UPLC System (Waters, Millford, MA, USA) coupled to an AQUITY RDa mass spectrometer (Waters, Milford, MA, USA) equipped with an ESI probe, in positive ion mode. The flow rate was set to 0.300 mL/min using 50 % methanol (aq) + 0.1 % formic acid eluent. Scan parameters were set as follows: analyzer mode, full scan; scan range 50-2000 m/z; scan rate, 2 Hz; cone voltage, 40 V; capillary voltage, 0.8 kV; desolvation temperature, 550 °C; and intelligent data capture, on.

NMR spectra were obtained using a Bruker Avance III HD nanobay NMR equipped with a 9.4 T magnet (^{1}H 400.2 MHz, ^{19}F 376.5 MHz, ^{13}C 100.6 MHz), Bruker Avance NMR equipped with a 11.75 T magnet and a ^{13}C detect cryoprobe (^{1}H 500.3 MHz, ^{13}C 125.8 MHz) and Bruker NEO 600 with broadband helium cryoprobe (^{1}H 600.4 MHz, ^{13}C 151.0 MHz). Chemical shifts were referenced to residual solvent peaks and are given as follows: chemical shift (δ, ppm), multiplicity (s, singlet; br, broad; d, doublet, t, triplet; q, quartet; m, multiplet), coupling constant (*J*, Hz), integration. All NMR spectra were recorded at 298K, unless stated otherwise.

LCMS (ESI) results were recorded on a Waters LCT Premier bench-top orthogonal acceleration time-of-flight LC-MS system, connected to a CTC Analytics HTS PAL Sample Manager, Acquity PDA Detector, Acquity Column Heater/Cooler, Acquity Binary Solvent Manager. Samples (5 µL; 10 µL sample loop) were injected onto an ACE equivalence C18 (50 ´ 2.1 mm) column according to the following gradient profile:

Time [min]	Flow rate [mL/min]	A MilliQ water + 0.1% HCOOH [%]	B Acetonitrile + 0.1% HCOOH [%]
**0**	0.3	95	5
**1**	0.3	95	5
**8.5**	0.3	5	95
**9**	0.3	5	95
**9.1**	0.3	95	5
**10**	0.3	95	5

HPLC analysis: Discovery®Cyano column [5 µm, 4.6 × 250 mm] fitted with a Discovery®Cyano guard column [5 µm, 2 × 40 mm]; [98:2 H_2O: MeCN; 5 min hold → 0:100 H_2O: MeCN: 20 min; 2 min hold; → 98:2 H_2O: MeCN: 2 min; 5 min hold; 1 mL/min].

UV-Vis spectra were recorded on a Jasco V-770 UV-Visible/NIR Spectrophotometer equipped with Peltier temperature controller and stirrer using quartz cuvettes of 1 cm path length. Experiments were conducted at 25°C unless otherwise stated.

Steady-state excitation and emission spectra were recorded on a Horiba Jobin Yvon Fluorolog® 3-12 Fluorometer equipped with a Hamamatsu R928 detector and a double-grating emission monochromator. S1/R1 response was used throughout as luminescence output. Emission spectra were recorded by exciting samples **1-4·Tb$_2$** (90 µM in 1X PBS buffered MilliQ® Water) at 488 nm, with a slit width of 23 nm and recording the emission between 520-800 nm with a band pass of 2 nm and 0.5 s integration time. A 2” square unmounted longpass 400 nm filter (FGL400S) fabricated using a 2 mm thick Schott® coloured glass from Thor labs was used while recording steady-state emission. Time-resolved lifetime measurements were made on Fluorolog® 3-12 for **1-4·Tb$_2$**. The complex concentration was at 90 µM both in protiated and deuterated conditions. Phosphate buffered saline was used as medium in both cases and the pH and pD were set using a calibrated pH meter (Hannah

Instruments pH 210 Microprocessor pH meter with a HI 1131B electrode). The pD of the heavy water solution was determined using the following equation: pD = 'pH meter reading' + 0.45.[1],[2] Luminescence lifetimes were obtained using exponential decay functions. S1 response was used throughout for obtaining the time-resolved spectra. Luminescence lifetimes were calculated using the modified Horrock's equation, $q = A (k_H – k_D – B)$ where A = 5.0 and B = 0.06 in water, k_H and k (in ms^{-1}) correspond to the rate constant of the lifetime decay in the given solvent and the corresponding deuterated solvent.

Abbreviations:

THF: tetrahydrofuran, **DCM**: dichloromethane, **NBS:** N-bromosuccinimide, **DMF**: Dimethylformamide, **Et_2O**; Diethyl ether, **EtOAc**: Ethyl acetate, **TBS**: tert-Butyldimethylsilyl **MeCN:** acetonitrile, **TFA:** trifluoroacetic acid, **EtOH:** ethanol, **MeOH:** methanol, ***ⁱ*PrOH**: Isopropanol, **DIPEA**: N,N-Diisopropylethylamine **rt:** room temperature, **HR-ESI-MS:** high resolution electro-spray ionisation mass spectrometry, **HPLC:** high performance liquid chromatography, **λ_{ex}:** excitation wavelength, **λ_{em}:** emission wavelength.

Synthesis and Characterisation of compounds:

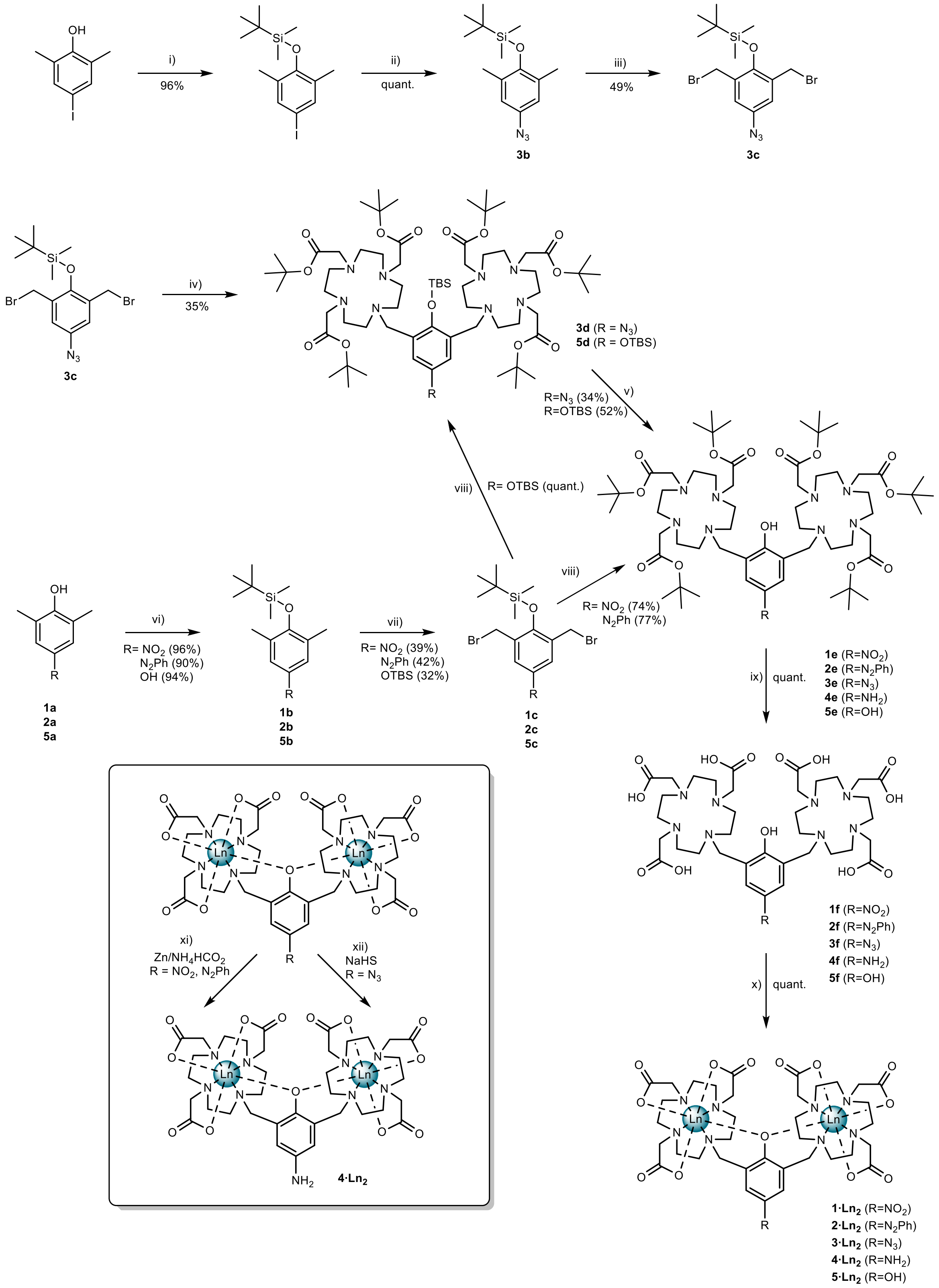

Scheme S1: Synthesis of all compounds i.) TBS-Cl, Imidazole, DMF, r.t., 16h ii.) NaN_3, CuI, NaAsc, DMEDA, EtOH:H_2O:DMSO (12:5:2), Δ, 40 min iii.) NBS, AIBN, CCl_4, Δ, 3h, iv) DO3A^{t}Bu, Na_2CO_3, MeCN, 60 °C, 48 h, v) TASF, DMF, r.t., 24 hrs, vi) TBS-Cl, Imidazole, DMF, r.t., 16h. vii.) NBS, $(BzO)_2$, CCl_4, Δ, 3h viii.) DO3A^{t}Bu, Na_2CO_3, MeCN, 60°C, 48 h ix.) TFA:DCM (1:1), r.t., 18h x.) Ln(OTf)$_3$, $NaOH_{(aq)}$, H_2O, 50°C, 24 h xi) Zn/NH_4HCO_2, H_2O, 37 °C, 48 h xii) NaHS, H_2O, 37 °C, 2 h.

General procedure for TBS protection

The appropriate phenol (4-iodo-2,6-dimethylphenol, **2a** or **3a**) (1.00 eqv.) and imidazole (4.00 eqv.) were dissolved in DMF (c_{phenol} = 0.7 M), to this TBS-Cl (2.00 eqv.) was added and the reaction mixture was left to stir for 24 hours. The reaction mixture was diluted with Et_2O and washed with brine: H_2O (1:1 mixture, minimum 5 times the volume of DMF used in the reaction) and extracted with Et_2O. After the first extraction the organic phase was further washed with brine. The organic phase was dried over $MgSO_4$, filtered and concentrated under reduced pressure. The crude product was then purified by silica gel flash column chromatography and eluted with petroleum ether: Et_2O (100:0 → 95:5) to obtain the pure product as a crystalline white (**1b**) solid, yellowish white (**2b**), deep orange/red oil (**3c**) or a off white oil (**5b**).

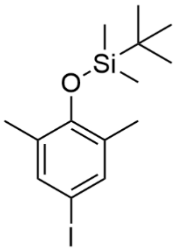

tert-butyl(4-iodo-2,6-dimethylphenoxy)dimethylsilane. Known compound,[3] Yield: 7.01 g (96%); R_f = 0.50 (Pentane, isocratic); ^{1}H NMR (400 MHz, $CDCl_3$) δ ppm 7.28 (s, 2H), 2.16 (s, 6H), 1.02 (s, 9H), 0.18 (s, 6H); ^{13}C NMR (101 MHz, $CDCl_3$) δ ppm 152.4, 137.4, 131.5, 84.3, 26.2, 18.9, 17.6, -2.8;

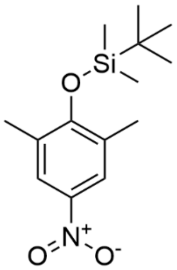

1b. Yield: 6.45 g (96%); R_f = 0.60 (95:5 Pentane/Et_2O); ^{1}H NMR (400 MHz, $CDCl_3$) δ 7.90 (s, 2H), 2.28 (s, 6H), 1.03 (s, 9H), 0.24 (s, 6H); ^{13}C NMR (101 MHz, $CDCl_3$) δ 158.4, 141.6, 129.8, 124.5, 26.1, 19.0, 18.1, -2.6; HR-ESI-MS obsd 282.1521, calcd 282.1520 [$(M + H)^+$, M = $C_{14}H_{23}NO_3Si$].

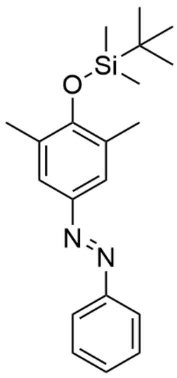

2b. Yield: 5.21 g (90%); column conditions: Hexane/EtOAc (98:2); ^{1}H NMR (600 MHz, $CDCl_3$) δ 7.88 – 7.84 (m, 2H), 7.61 (s, 2H), 7.52 – 7.41 (m, 3H), 2.31 (s, 6H), 1.05 (s, 9H), 0.24 (s, 6H). ^{13}C NMR (151 MHz, $CDCl_3$) δ 155.5, 153.0, 147.1, 130.4, 129.5, 129.2, 123.8, 122.7, 26.2, 19.0, 18.10, -2.7. HR-ESI-MS obsd 341.2044, calcd 341.2044 [$(M + H)^+$, M = $C_{20}H_{28}N_2OSi$].

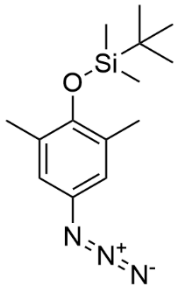

3b. This compound was prepared by adapting and modifying a literature procedure.[4] *tert*-butyl(4-iodo-2,6-dimethylphenoxy)dimethylsilane (1.50 g, 4.14 mmol), sodium azide (0.54 g, 8.27 mmol) and DMEDA (134 µL, 1.24 mmol) were dissolved/suspended in a mixture of EtOH:H_2O:DMSO (6:2.5:1 mL, respectively). After degassing for 10 mins with Argon, copper(I) iodide (0.08 g, 0.41 mmol) and sodium ascorbate (0.04 g, 0.21 mmol) were added. The reaction mixture was heated to reflux and left to stir for 40 minutes. Upon completion, the reaction mixture was cooled to room temperature, washed with brine (100 mL) and extracted with EtOAc (150 mL), and Et_2O (150 mL). The combined organic phase was dried over $MgSO_4$, filtered, and concentrated under reduced pressure. The resulting oil was then purified by silica gel flash column chromatography and eluted with petroleum ether. The pure product was obtained as a yellow oil. Yield: 1.13 g (quant.); R_f = 0.30 (Pentane, isocratic); ^{1}H NMR (400 MHz, DMSO-d_6) δ ppm 6.65 (s, 2H), 2.20 (s, 6H), 1.03 (m, 1.03), 0.18 (s, 6H); ^{13}C NMR (101 MHz, DMSO-d_6) δ ppm 149.6, 132.4, 130.3, 119.1, 26.2, 18.9, 18.0, -2.9.

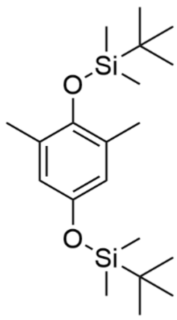

5b. Known compound.[5] 2,6-Dimethylhydroquinone (2.00 g, 14.47 mmol) and imidazole (5.91 g, 86.79 mmol) were dissolved in 20 mL DMF followed by the addition of TBS-Cl (6.54 g, 43.40 mmol). The resulting mixture was stirred for 2 days at room temp. After TLC analysis showed full conversion and the formation of the product, the reaction mixture was diluted with approx. 10 mL Et_2O and was poured into a separation funnel which already contained 250 mL brine and 300 mL Et_2O. After the first extraction the organic layer was separated and was further extracted with dist. water (2×250 mL). The organic layer was then dried over $MgSO_4$, filtered and concentrated under vacuo. The resulting crude product was purified by column chromatography (Silica, Petroleum ether:Et_2O, 100:0 → 95:5 → 90:10) resulting a white solid. Yield: 5.01 g (94%); ^{1}H NMR (400 MHz, $CDCl_3$) δ ppm 6.45 (s, 2H), 2.15 (s, 6H), 1.02 (s, 9H), 0.97 (s, 9H), 0.16 (s, 12H); ^{13}C NMR (101 MHz, $CDCl_3$) δ ppm 149.3, 146.4, 129.3, 119.9, 26.3, 25.9, 18.9, 18.3, 18.1, -2.9, -4.3; HR-ESI-MS obsd 367.2473, calcd 367.2483 [$(M + H)^+$, M = $C_{20}H_{38}O_2Si_2$].

General procedure for radical dibromination

The appropriate TBS-protected dimethyl phenol (1.00 eqv.) was dissolved in CCl_4 ($c_{s.m.}$ = 0.1 M) followed by the addition of NBS (2.10 eqv.)). To this benzoyl peroxide was added (0.05 eqv.). The reaction mixture was heated to reflux and left to stir for a minimum of 3 hours. The resulting suspension was left to cool to room temperature and filtered. The filtrate was concentrated under reduced pressure and the crude mixture was then purified by silica gel flash column chromatography and eluted with pentane: Et_2O (100:0 → 99:1 → 98:2 → 97:3 → 96:4 → 95:5 → 90:10 for **1c**) or pentane (isocratic method for **2c** and **3c**) resulted the pure product as a white (**1c**), orange (**2c**), yellow (**3c**) or a white (**5c**) crystalline solid.

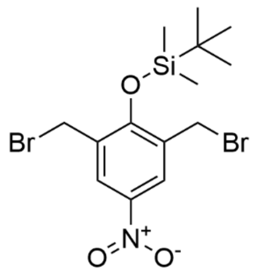

1c. Yield: 1.51 g (39%); R_f = 0.30 (95:5 Pentane/Et_2O); ^1H NMR (400 MHz, $CDCl_3$) δ 8.28 (s, 2H), 4.49 (s, 4H), 1.10 (s, 9H), 0.35 (s, 6H); ^{13}C NMR (101 MHz, $CDCl_3$) δ 156.4, 142.2, 130.8, 127.4, 27.3, 26.1, 19.1, -3.1; ESI-MS obsd 502.98, calcd 502.98 $(M + MeCN + Na)^+$.

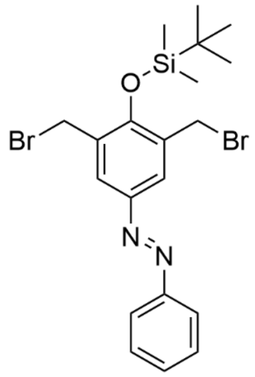

2c. Yield: 1.43 g (42%); R_f = 0.25 (pentane, isocratic); ^1H NMR (600 MHz, $CDCl_3$) δ 7.99 (2H) 7.90 – 7.89 (d, 2H), 7.53 – 7.48 (m, 2H), 7.47 – 7.46 (m, 1H), 4.58 (s, 4H), 1.11 (s, 9H), 0.34 (s, 6H). ^{13}C NMR (151 MHz, $CDCl_3$) δ 153.5, 152.7, 147.4, 131.1, 130.2, 129.3, 126.8, 122.9, 34.3, 28.8, 26.2, 22.5, 19.1, 14.2, -3.1. HR-ESI-MS obsd 497.0253, calcd 497.0254 [$(M + H)^+$, M = $C_{20}H_{27}Br_2N_2OSi$].

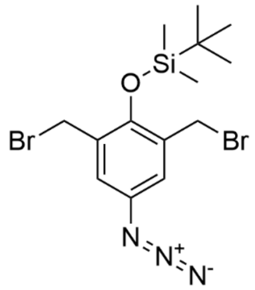

3c. Yield: 2.98 g (49%); R_f = 0.20 (pentane, isocratic); ^1H NMR (400 MHz, $CDCl_3$) δ ppm 7.03 (s, 2H), 4.46 (s, 4H), 1.08 (s, 9H), 0.28 (s, 6H); ^{13}C NMR (101 MHz, $CDCl_3$) δ ppm 148.1, 134.1, 130.9, 122.5, 28.4, 26.2, 19.0, -3.30;

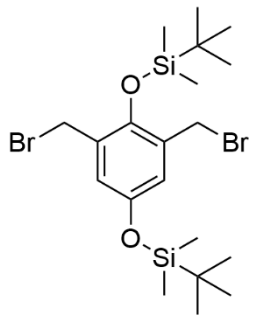

5c. Yield: 1.60 g (32%); Column on Silica gel: (Pent: Et_2O = 100:0 → 90:10); ^{1}H NMR (400 MHz, $CDCl_3$) δ ppm 6.85 (s, 2H), 4.45 (s, 4H), 1.08 (s, 9H), 0.98 (s, 9H), 0.26 (s, 6H), 0.20 (s, 6H); ^{13}C NMR (101 MHz, $CDCl_3$) δ ppm 150.0, 145.1, 129.9, 123.5, 29.2, 26.2, 25.8, 19.0, 18.3, -3.4, -4.3; HR-ESI-MS obsd 525.0650, calcd 525.0673 [$(M + H)^+$, M = $C_{20}H_{36}Br_2O_2Si_2$].

Procedures for the protected macrocycles

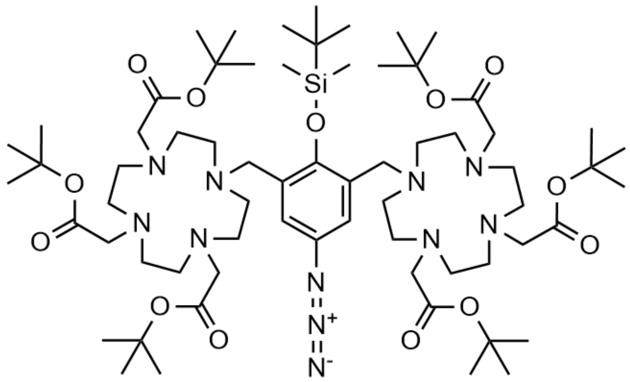

3d. DO3A^{t}Bu.HBr (1.16 g, 1.95 mmol, 1.75 eqv) was dissolved in MeCN (27 mL) followed by the addition of Na_2CO_3 (365 mg, 3.45 mmol, 3.00 eqv), to this **3c** (500 mg, 1.15 mmol, 1.00 eqv) was added and the reaction mixture was let to stir for 2.5 days at 55°C. The resulting suspension was filtered and the filtrate concentrated under reduced pressure. The resulting crude mixture was redissolved in a small amount of DCM then purified by silica gel flash column chromatography and eluted with DCM: MeCN (100:0 → 80:20 → 60:40 → 40:60 → 20:80) to obtain the pure product as a light mustard-coloured solid. Yield: 370 mg (35%); R_f = 0.23 (80:10:10 DCM/MeCN/MeOH); ^{1}H NMR (400 MHz, $CDCl_3$) δ ppm 7.02 (s, 2H), 4.03–1.68 (m, 48H), 1.61–1.24 (m, 54H), 1.10–0.80 (m, 9H), 0.25–(–0.21)(m, 6H); ^{13}C NMR (151 MHz, $CDCl_3$) δ ppm 173.8, 172.6, 151.0, 134.0, 129.6, 120.6, 83.1, 82.6, 56.5, 55.8, 52.1, 49.2, 54.5–47.0, 28.1, 28.0, 27.9, 26.4, 18.6, -3.3; HR-ESI-MS obsd 1302.8832, calcd 1302.8831 [$(M + H)^+$, M = $C_{66}H_{119}N_{11}O_{13}Si$].

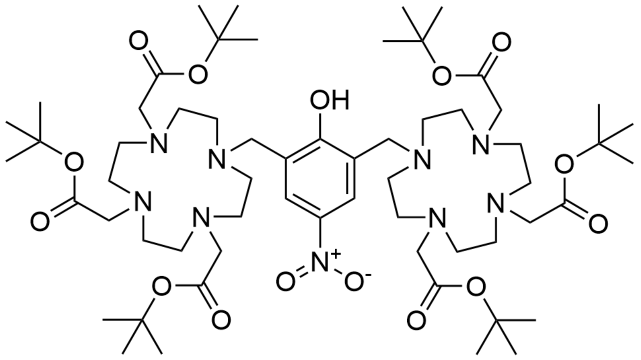

1e. This known compound was prepared via a novel procedure.[6] DO3A^{t}Bu.HBr (2.37 g, 3.98 mmol, 1.75 eqv) was dissolved in 53 mL MeCN followed by the addition of Na_2CO_3 (0.72 g, 6.83 mmol, 3.00 eqv). To this **1c** (1.00 g, 2.28 mmol, 1.00 eqv) was added and the reaction mixture was let to stir at 50°C for 2 days. The resulting suspension was filtered, and the filtrate was concentrated under reduced pressure. The residue was redissolved in a minimum amount of DCM:Acetone (1:1) mixture and then purified by silica gel flash column chromatography and eluted with DCM:Acetone:^{i}PrOH:MeOH (50:50:0:0 → 50:45:5:0 → 50:40:10:0 → 50:40:5:5). The resulting product was redissolved in $CHCl_3$ and was filtered through a membrane filter (Nylon, 0.45 µm pore size) and evaporated to dryness to afford the pure product as a bright-yellow solid. Yield: 1.51 g (74%); R_f = 0.37 (90:10 CH_2Cl_2/MeOH); ^{1}H NMR (400 MHz, $CDCl_3$) δ ppm 7.99 (s, 2H), 4.24–1.77 (m, 48H), 1.76–0.97 (m, 54H); ^{13}C NMR (101 MHz, $CDCl_3$) δ ppm 179.0, 172.6, 129.3, 128.6, 126.9, 82.4, 82.1, 55.9, 55.2, 54.4–50.4, 50.4–47.5, 46.6–43.9, 28.3, 28.1; HR-ESI-MS obsd 1192.7802, calcd 1192.7803 [(M + H)$^+$, M = $C_{60}H_{105}N_9O_{15}$].

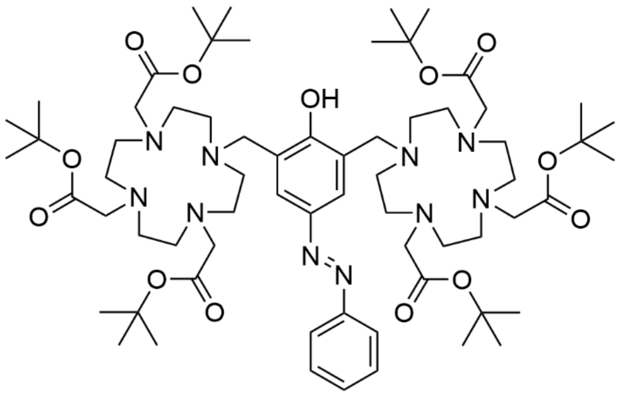

2e. DO3A^{t}Bu.HBr (602 mg, 1.01 mmol, 1.80 eqv) was dissolved in MeCN (14 mL) followed by the addition of Na_2CO_3 (215 mg, 2.02 mmol, 3.60 eqv). To this **3c** (280 mg, 0.56 mmol, 1.00 eqv) was added and the reaction mixture was heated at 60°C for 2 days. The resulting suspension was filtered, and the filtrate concentrated under reduced pressure. The dark red coloured residue was redissolved in a minimum amount of DCM:MeCN (8:2) mixture, then purified by silica gel flash column chromatography and eluted with CH_2Cl_2:MeCN:^{i}PrOH:MeOH

(80:20:0:0 → 60:40:0:0 → 60:38:2:0 → 60:30:10:0 → 60:20:20:0 → 60:20:15:5 → 60:20:10:10 → 60:20:0:20 → 60:0:0:40) to obtain the product. The product was redissolved in $CHCl_3$ and was filtered through a membrane filter (Nylon, 0.45 μm pore size) and evaporated to dryness to afford the pure product as a dark orange/red solid. Yield: 435 mg (77%); R_f = 0.20 & 0.30 (60:28:8:4 DCM/MeCN/^{i}PrOH/MeOH); ^{1}H NMR (400 MHz, $CDCl_3$) δ ppm 8.99 (br, 1H), 8.12 (s, 2H), 7.78 (m, 2H), 7.39 (m, 3H), 4.75–1.67 (m, 48H), 1.66–0.87 (m, 54H); ^{13}C NMR (101 MHz, $CDCl_3$) δ ppm 173.3, 172.6, 172.1, 170.6, 157.2, 152.7, 147.4, 130.6, 128.8, 126.2, 122.6, 121.9, 82.7, 82.5, 82.4, 56.4, 56.0, 55.8, 55.4, 54.6, 52.5, 51.4, 50.4, 58.0–47.0, 28.3, 28.1, 28.0; HR-ESI-MS obsd 1251.8360, calcd 1251.8327 [$(M + H)^+$, M = $C_{66}H_{110}N_{10}O_{13}$].

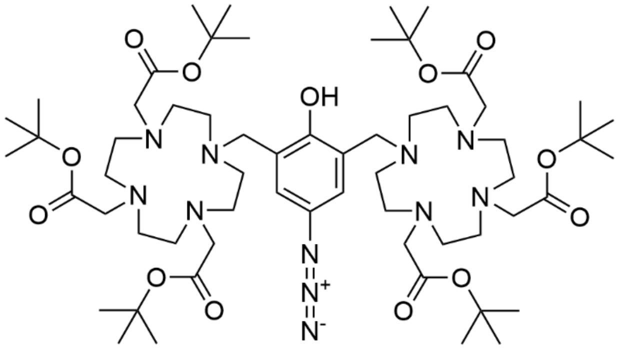

3e. In a plastic reaction vessel **3d** (1.29 g, 0.99 mmol, 1.00 eqv) was dissoved in DMF (10 mL). To this stirred solution TASF (600 mg (2.18 mmol, 2.20 eqv) in 3 mL DMF) was added and the resulting reaction mixture was allowed to stir at room temperature for 18 hours. After TLC and mass spectrometry analysis showed full conversion to the product the reaction was worked up. The reaction mixture was transferred to a separatory funnel which had already contained 80 mL brine:H_2O (1:1) mixture. The product was extracted into EtOAc (2 × 100 mL). The combined organic layer was washed/dried with 30 mL brine and separated. The organic layer was dried over $MgSO_4$, filtered and concentrated on the rotavap. The resulting dark green/brown coloured crude was purified by column chromatography on silica gel (eluted with DCM:Acetone:MeOH (100:0:0 → 80:20:0 → 80:18:2 → 80:16:4 → 80:12:8 → 80:8:12 → 80:0:20 → 70:0:30)) to afford the pure product as a green solid. Yield: 403 mg (34%); R_f = 0.21 ((90:10 CH_2Cl_2/MeOH); ^{1}H NMR (400 MHz, $CDCl_3$) δ ppm 9.63 (s, 1H), 7.07 (m, 2H), 4.83–1.66 (m, 48H), 1.64–1.12 (m, 54H); ^{13}C NMR (126 MHz, DMSO-d_6) δ ppm 173.3, 172.6, 171.9, 171.1, 170.7, 170.6, 169.8, 154.6, 151.8, 133.7, 131.9, 130.5, 125.8, 124.6, 121.2, 119.9, 119.4, 82.9, 82.5, 82.4, 81.1, 58.2, 57.9, 56.3, 56.1, 56.0, 55.8, 55.4, 54.5, 52.6, 51.9, 51.6, 51.4, 49.7, 49.3, 47.6, 54.0–47.0, 28.3, 28.2, 28.0, 27.9. HR-ESI-MS obsd 1188.7931, calcd 1188.7966 [$(M + H)^+$, M = $C_{60}H_{105}N_{11}O_{13}$].

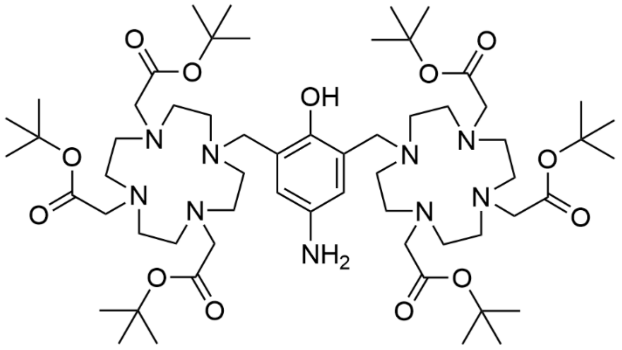

5e. 3e (365 mg, 0.31 mmol) was dissolved in EtOH (5 mL) to this 10% Pd on carbon (55-65% wet, 109 mg, 30 m/m%) was added. The mixture was placed under hydrogen atmosphere and the reaction was left to run at room temperature for 5 days, balloons replaced after 2 days. The resulting suspension was filtered through a pad of Celite and concentrated under reduced pressure. The crude mixture was then purified by silica gel flash column chromatography and eluted with DCM:MeOH (100:0 → 98:2 → 96:4 → 94:6 → 92:8 → 90:10 → 85:15 → 80:20 → 75:25) to obtain the pure product as a brown solid. Yield: 264 mg (74%); R_f = 0.24 (90:10 CH_2Cl_2/MeOH); ^{1}H NMR (400 MHz, DMSO-d_6) δ ppm 10.24 (s, 1H), 6.55–6.15 (m, 2H), 4.38 (s, 2H), 3.77–1.84 (m, 48H), 1.52–1.34 (m, 54H); ^{13}C NMR (151 MHz, $CDCl_3$) δ ppm 173.4, 171.9, 107.6, 169.7, 145.1, 141.7, 131.0, 117.9, 82.3, 82.2, 58.2, 57.7–55.8, 55.3, 53.9–47.0, 31.0, 28.6–27.8; HR-ESI-MS obsd 1162.8081, calcd 1162.8061 [$(M + H)^+$, M = $C_{60}H_{107}N_9O_{13}$].

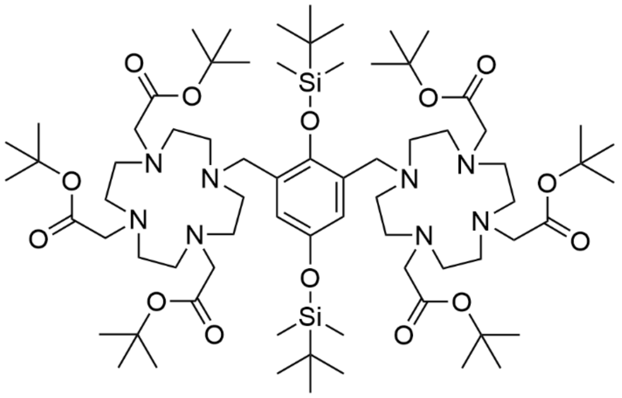

5d. DO3A^{t}Bu (2.18 g, 3.66 mmol) and Na_2CO_3 (0.65 g, 6.10 mmol) were measured into a 100 mL rbf and dissolved in MeCN (50 mL). **5c** (1.28 g, 2.44 mmol) was added and the flask was equipped with a condenser and was brought to 70°C with the use of an oil bath. This mixture was let to stir for 2 days. After TLC analysis showed full consumption of the dibromo starting material, the reaction mixture was cooled down to room temp. and the solid was filtered off. The filtrate was concentrated under reduced pressure and the resulting crude residue was loaded onto a silica gel column. Elution with DCM:Acetone:^{i}PrOH:MeOH (60:40:0:0 → 50:50:0:0 → 50:49:1:0 → 50:47:3:0 → 50:45:5:0 → 50:40:10:0 → 50:40:5:5 → → 50:40:0:10) resulted **5d** as a yellowish white solid. (Note: the eluent mixture used for col.

chromatography dissolves silica gel reasonably well. After the pure fractions are combined and dried, the product is redissolved in $CHCl_3$, and silica gel can be removed/separated by centrifugation or membrane filtration (nylon, 0.45 μm). Yield: 1.59 g (quant.); ^1H NMR (400 MHz, $CDCl_3$) δ ppm 6.73 (s, 2H), 3.94-1.68 (m, 48H), 1.56-1.35 (m, 54H), 0.94 (s, 9H), 0.87 (s, 9H), 0.07 (s, 6H), 0.00 (s, 6H); ^{13}C NMR (151 MHz, $CDCl_3$) δ ppm 173.7, 172.8, 150.0, 148.5, 128.0, 122.8, 82.9, 82.5, 56.5, 55.9, 54.0-47.0, 28.2, 27.9, 26.6, 25.8, 18.6, 18.3; HR-ESI-MS obsd 1391.9629, calcd 1391.9631 [$(M + H)^+$, M = $C_{72}H_{134}N_8O_{14}Si_2$].

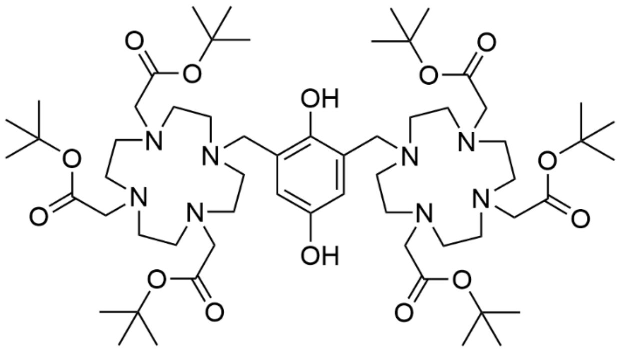

5e. **5d** (0.36 g, 0.28 mmol) was dissolved in 2.25 mL THF and while continuously stirred TBAF (0.56 mL, 1 M in THF, 0.56 mmol) was added. The reaction mixture was let to stir at room temperature for 16 hours. After TLC analysis showed full conversion, the reaction was worked up by transferring it to a separation funnel which had contained brine (40 mL, +2 drops of 1M HCl) and DCM (40 mL) and extracted. The aqueous layer was extracted with DCM once more (40 mL) and the combined organic phase was dried over $MgSO_4$, filtered, and concentrated under reduced pressure. The crude mixture was purified with column chromatography on silica gel. Elution with DCM:MeOH (100:0 → 98:2 → 95:5 → 90:10 → 85:5 → 80:20 → 75:25) resulted the pure product as a brown solid. Yield: 0.17 g (52%); ^1H NMR (400 MHz, $CDCl_3$) δ ppm 8.11 (s, 1H), 7.62 (s, 1H), 7.01 (s, 2H), 3.89-1.77 (m, 48H), 1.72-1.24 (m, 54H); ^{13}C NMR (151 MHz, $CDCl_3$) δ ppm 173.5, 171.7, 170.6, 169.7, 152.2, 145.2, 129.0, 119.5, 82.3, 82.1, 81.7, 58.3, 57.9-56.3, 55.0, 54.0-47.4, 28.7-27.8; HR-ESI-MS obsd 1163.7907, calcd 1163.7912 [$(M + H)^+$, M = $C_{60}H_{106}N_8O_{14}$].

General procedure for tert-butyl ester deprotection

The *tert*-butyl ester protected ligand (1 eqv.) was dissolved in DCM and then TFA was added dropwise (1:1, c = 0.045 M). The reaction mixture was left to stir at room temperature for 18 hours. The reaction mixture was concentrated under reduced pressure and the crude product redissolved in a minimum amount of MeOH (1 volume), this was added dropwise to Et_2O (12 volumes in a falcon tube) and a precipitate formed. The resulting suspension was centrifuged, and the supernatant was decanted. The remaining solid was redissolved in a minimal amount

of MeOH. The precipitation-centrifugation procedure was repeated twice more (overall 3 times). The resulting solids were dried to obtain the pure products as yellow (**1f**), red (**2f**), reddish brown (**3f**), brown (**4f**) and white (**5f**) solids.

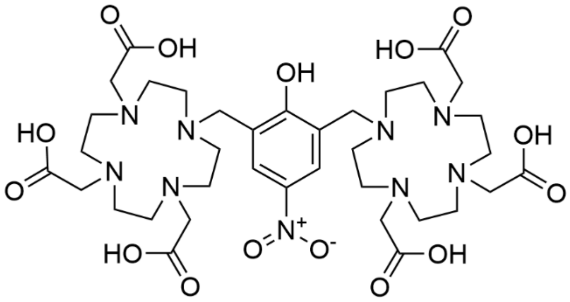

1f. Known compound.[6] Yield: 516 mg (quant.); ^{1}H NMR (400 MHz, D_2O) δ ppm 8.41 (s, 2H), 4.64–2.56 (m, 48H); ^{13}C NMR (151 MHz, D_2O) δ ppm 175.5–169.0, 160.4, 141.7, 130.3, 58.0–53.1, 53.1–46.2. HR-ESI-MS obsd 856.4057, calcd 856.4047 [(M + H)$^+$, M = $C_{36}H_{57}N_9O_{15}$].

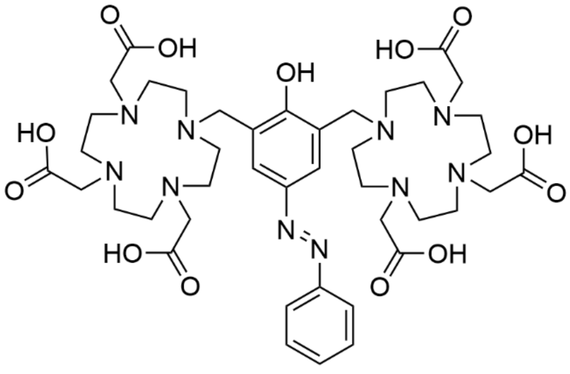

2f. Yield: 293 mg (96%); ^{1}H NMR (400 MHz, D_2O) δ ppm 8.09 (m, 2H), 7.89 (m, 2H), 7.59 (m, 3H), 4.58–2.48 (m, 48H); ^{13}C NMR (151 MHz, D_2O) δ ppm 176.8, 175.0, 170.4. 170.1, 169.1, 176.0–169.0, 157.9, 152.5, 146.4, 132.3, 130.2, 123.2, 57.5–47.0, 46.5, 42.9, 41.8. HR-ESI-MS obsd 915.4600, calcd 915.4571 [(M + H)$^+$, M = $C_{42}H_{62}N_{10}O_{13}$].

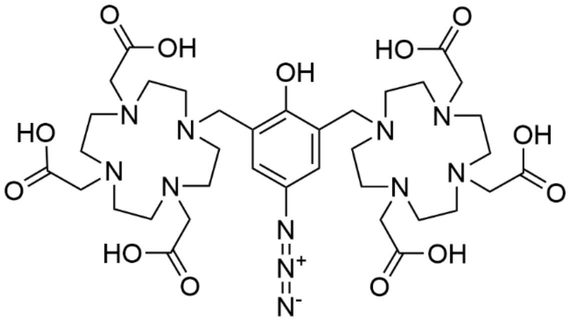

3f. Yield: 107 mg (quant.); ^{1}H NMR (400 MHz, D_2O) δ ppm 7.24 (s, 2H), 4.56–2.44 (m, 48H); ^{13}C NMR (151 MHz, D_2O) δ ppm 176.3–168.6, 154.4, 151.1, 134.9, 125.1, 58.0–46.0, 42.9. HR-ESI-MS obsd 852.4175, calcd 852.4210 [(M + H)$^+$, M = $C_{36}H_{57}N_{11}O_{13}$].

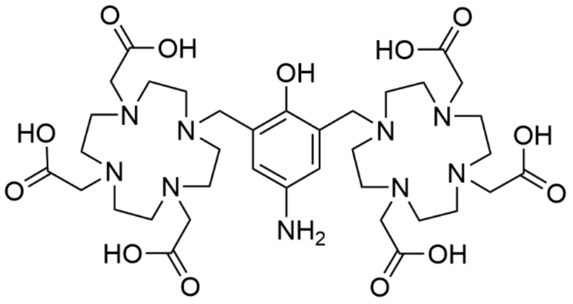

4f. Yield: 124 mg (quant.); ^{1}H NMR (400 MHz, D_2O) δ ppm 7.56 (s, 2H), 4.53–2.51 (m, 48H); ^{13}C NMR (151 MHz, D_2O) δ ppm 176.5–169.0, 154.3, 129.4–126.3, 124.1, 57.8–53.4, 53.4–46.1, 43.0. HR-ESI-MS obsd 826.4309, calcd 826.4305 [$(M + H)^+$, M = $C_{36}H_{59}N_9O_{13}$].

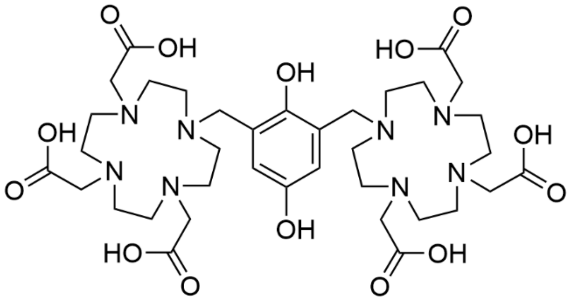

5f. Yield: 73 mg (quant.); ^{1}H NMR (400 MHz, D_2O) δ ppm 7.06 (s, 2H), 4.64-2.51 (m, 48H); ^{13}C NMR (151 MHz, D_2O) δ ppm 175.2-168.8, 150.7, 147.7, 121.6, 58.0-46.0, 43.0; ESI-MS obsd 827.32, calcd 827.42 [$(M + H)^+$, M = $C_{36}H_{58}N_8O_{14}$].

General procedure for obtaining the binuclear complexes

The appropriate ligand (1 eqv.) and the corresponding Ln-triflate (2.4 eqv.) were dissolved in H_2O (c_{ligand} = 0.05M). The reaction mixture was heated to 50 °C and left to stir for 1 day. To this NaOH (1M aqueous solution) was added in portions (3 + 3 + 1(or 2) eqv.) and left to stir at 50 °C for further 2 days. The resulting solution was purified by dialysis (FLOAT-A-LYZER G2 500-1000D CE) for 3 days to remove inorganic contaminants. After the dialysis was complete, the complex solution was concentrated and dried under high vacuum to give a yellow (**1·Ln₂**), bright orange (**2·Ln₂**) dark brown (**3·Ln₂**) deep purple (**4·Ln₂**) and an off-white (**5·Ln₂**) solid.

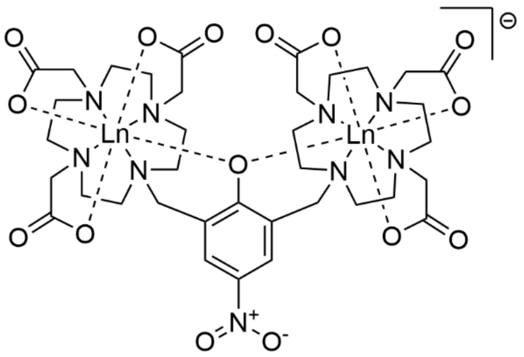

1·Eu₂.[6] Yield: 156 mg (93%); t_R = 12.99, 14.34 min (CN col., 0.1% CH_3COOH); HR-ESI-MS obsd 1154.1976, calcd 1154.1988 [$(M + H)^+$, M = $C_{36}H_{51}Eu_2N_9O_{15}$].

1·Tb₂.Yield: 151 mg (91%); t_R = 10.64 min (CN col., 0.1% HCOOH); HR-ESI-MS obsd 1168.2071, calcd 1168.2084 [$(M + H)^+$, M = $C_{36}H_{51}Tb_2N_9O_{15}$].

1·Gd₂.[6] Yield: 77 mg (84%); t_R = 11.15, 12.59 min (CN col., 0.1% HCOOH); HR-ESI-MS obsd 1166.2066, calcd 1166.2060 [$(M + H)^+$, M = $C_{36}H_{51}Gd_2N_9O_{15}$].

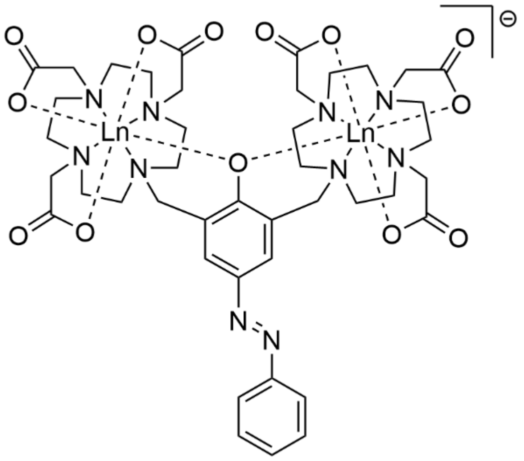

2·Eu₂. Yield: 35 mg (75%); t_R = 15.31, 15.54 min (CN col., 0.1% HCOOH); HR-ESI-MS obsd 1215.2495, calcd 1215.2526 [$(M + H)^+$, M = $C_{42}H_{57}N_{10}O_{13}Eu_2$].

2·Tb₂.Yield: 52 mg (81%); t_R = 15.21, 15.59 min (CN col., 0.1% HCOOH); HR-ESI-MS obsd 1227.2560, calcd 1227.2608 [$(M + H)^+$, M = $C_{42}H_{57}N_{10}O_{13}Tb_2$].

2·Gd₂.Yield: 31 mg (68%); t_R = 15.23, 15.50 min (CN col., 0.1% HCOOH); HR-ESI-MS obsd 1225.2581, calcd 1225.2583 [$(M + H)^+$, M = $C_{42}H_{57}N_{10}O_{13}Gd_2$].

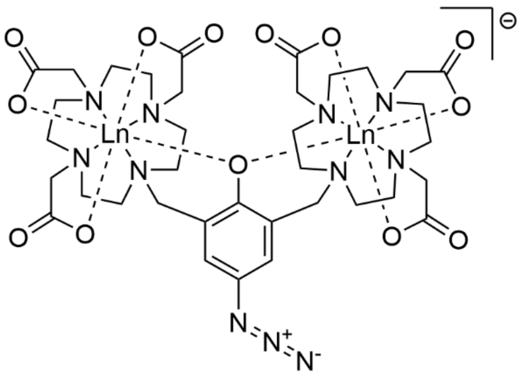

3·Eu₂. Yield: 67 mg (58%); t_R = 12.69 min (CN col., 0.1% HCOOH); HR-ESI-MS obsd 1150.2137, calcd 1150.2152 [$(M + H)^+$, M = $C_{36}H_{51}Eu_2N_{11}O_{13}$].

3·Tb₂. Yield: 65 mg (55%); t_R= 11.51 min (CN col., 0.1% HCOOH); HR-ESI-MS obsd 1164.2260, calcd 1164.2248 [$(M + H)^+$, M = $C_{36}H_{51}Tb_2N_{11}O_{13}$].

3·Gd₂. Yield: 44 mg (38%); t_R= 11.56 min (CN col., 0.1% HCOOH); HR-ESI-MS obsd 1162.2225, calcd 1162.2223 [$(M + H)^+$, M = $C_{36}H_{51}Gd_2N_{11}O_{13}$].

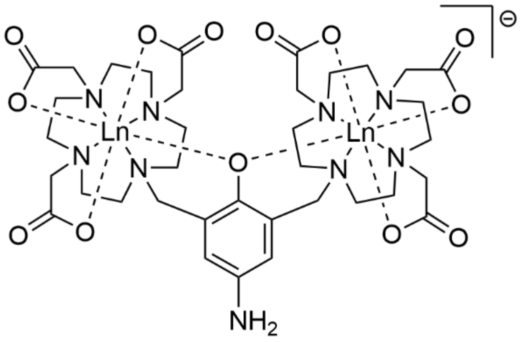

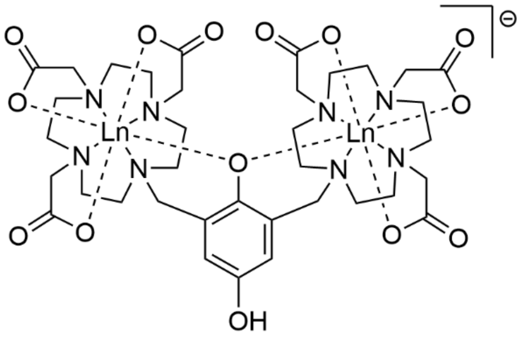

5·Eu₂. Yield: 20 mg (quant.); ESI-MS obsd 1125.2, calcd 1125.21 [$(M + H)^+$, M = $C_{36}H_{53}N_9O_{13}Gd_2$].

^{1}H NMR spectra of Ln(III) complexes:

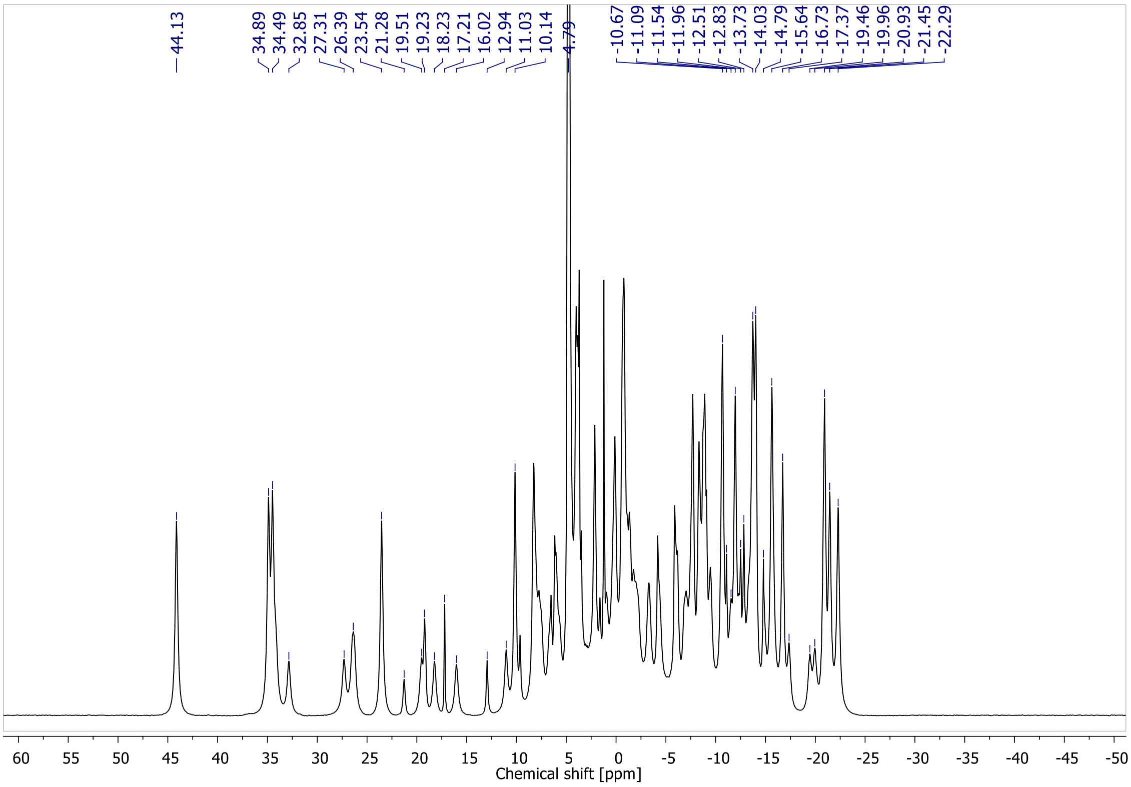

*Figure S1: ^{1}H NMR spectrum of **1·Eu$_2$**. (400 MHz, D_2O).*

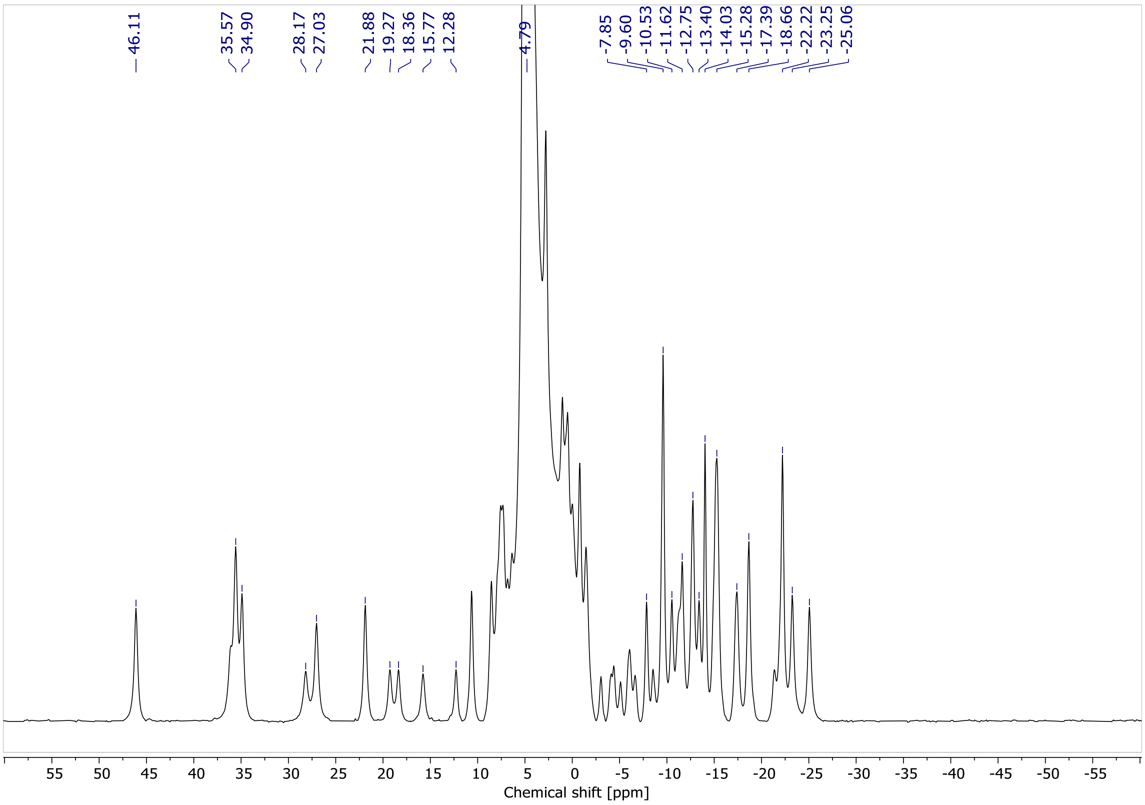

*Figure S2: ^{1}H NMR spectrum of **2·Eu$_2$**. (400 MHz, D_2O).*

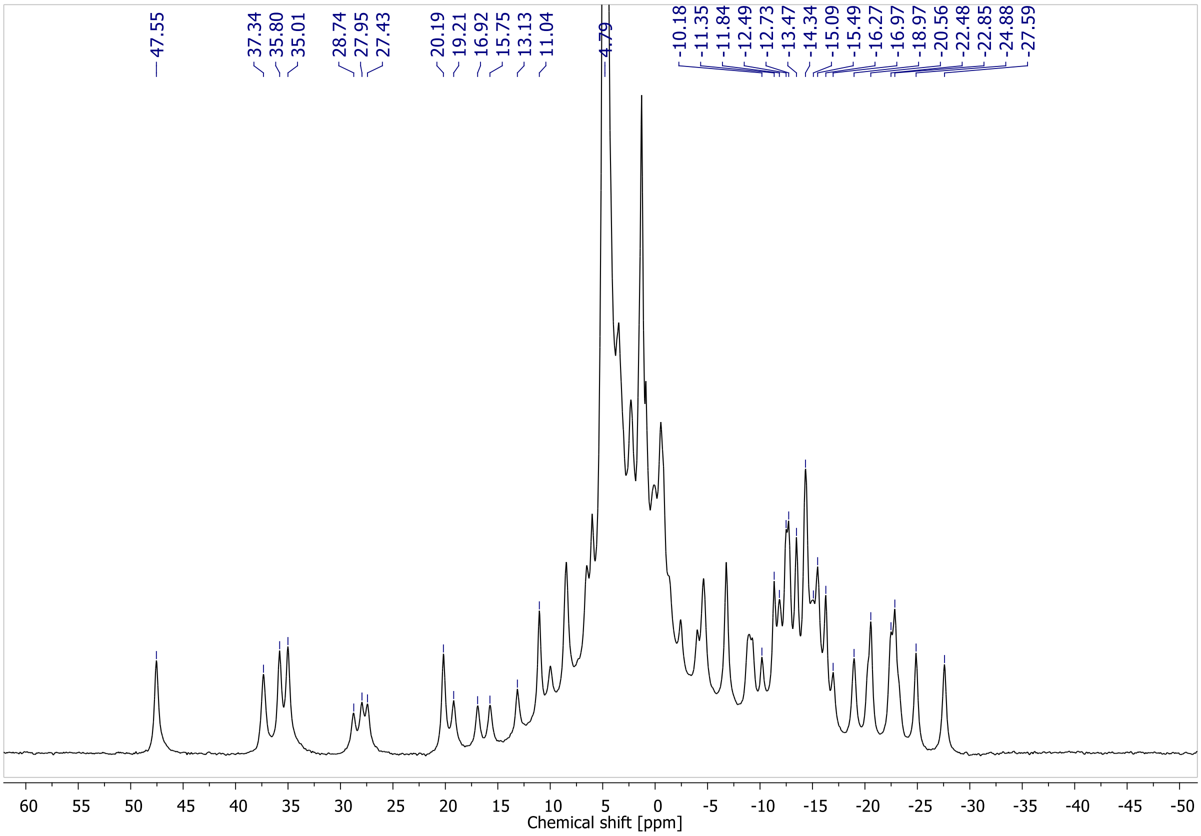

*Figure S3: ^{1}H NMR spectrum of **3·Eu₂**. (400 MHz, D₂O).*

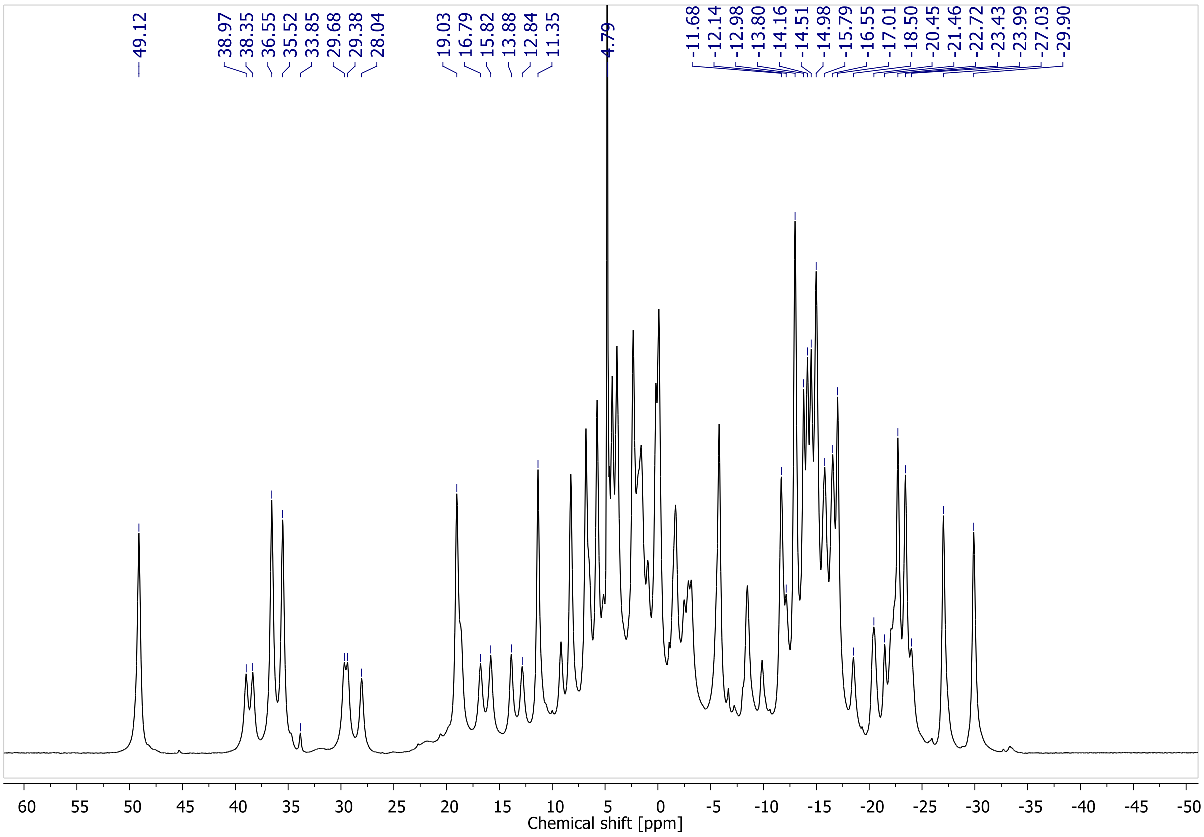

*Figure S4: ^{1}H NMR spectrum of **4·Eu₂** (400 MHz, D₂O).*

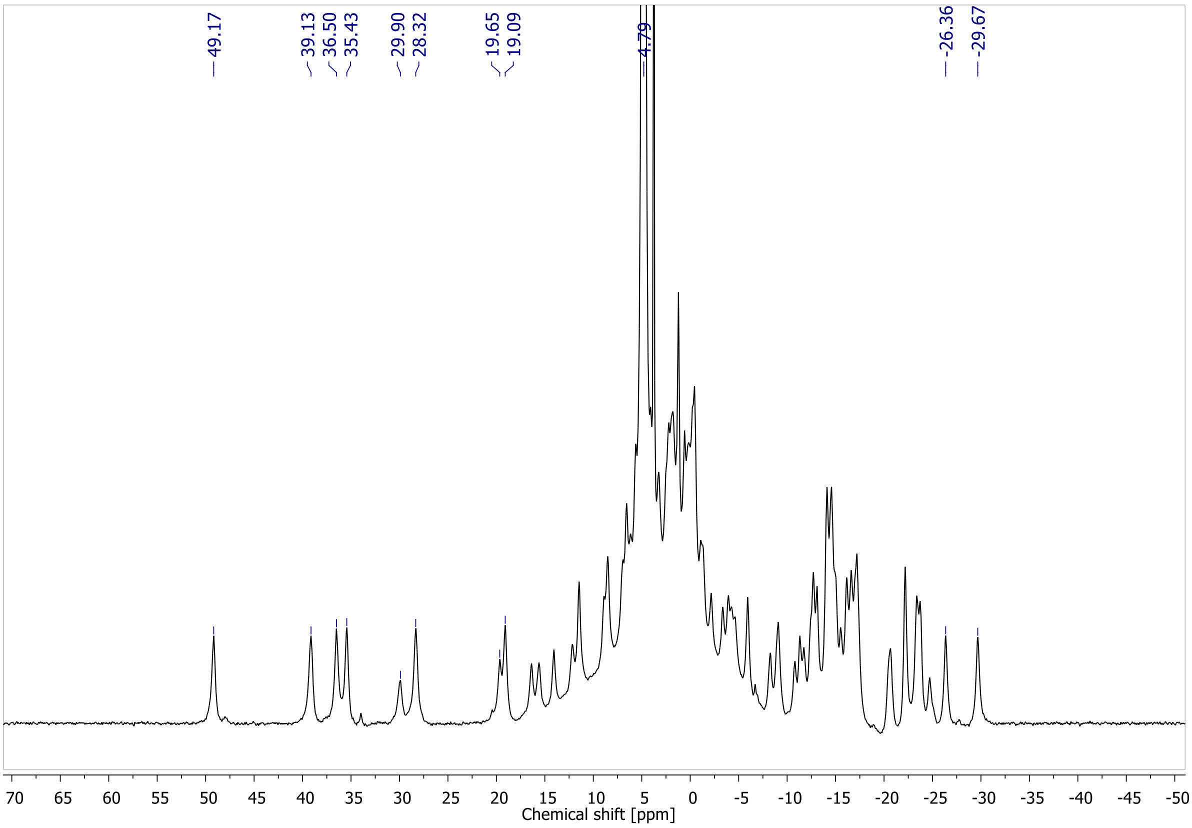

*Figure S5: ^{1}H NMR spectrum of **5·Eu₂** (400 MHz, D₂O).*

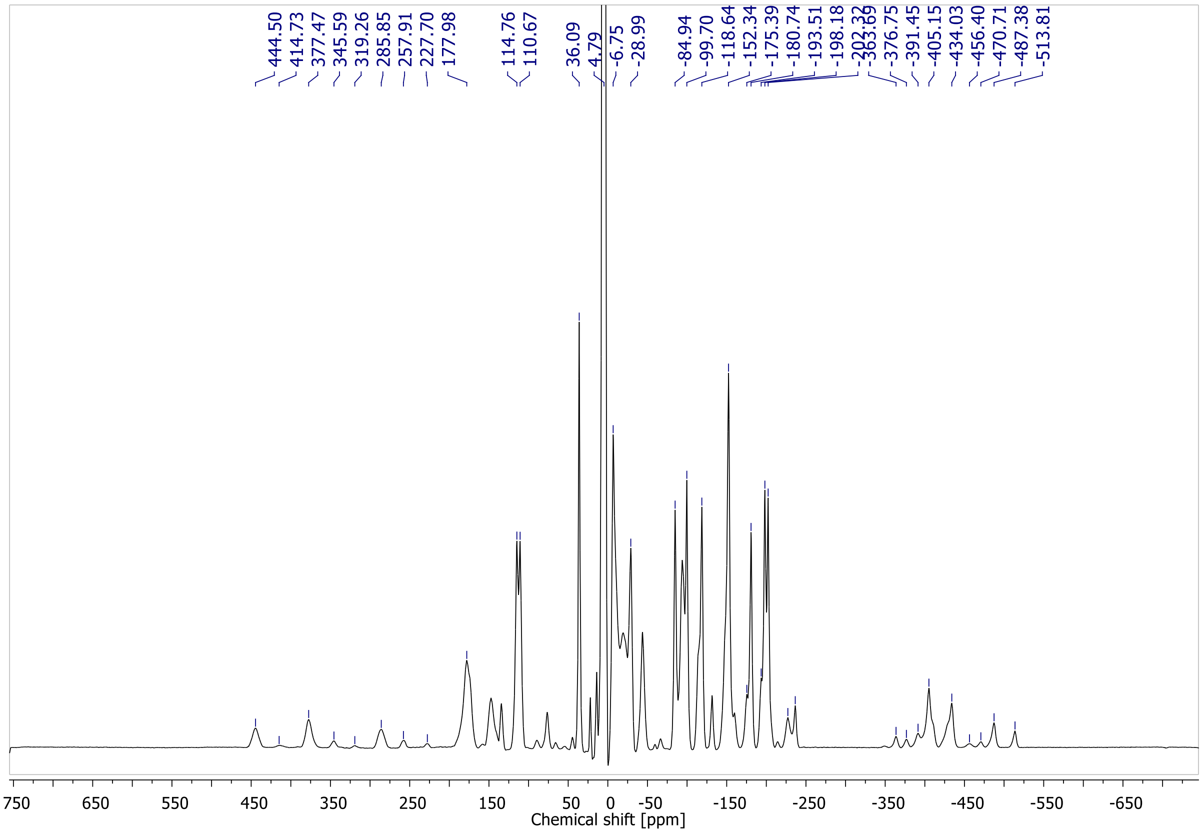

*Figure S6: ^{1}H NMR spectrum of **1·Tb₂** (500 MHz, D₂O).*

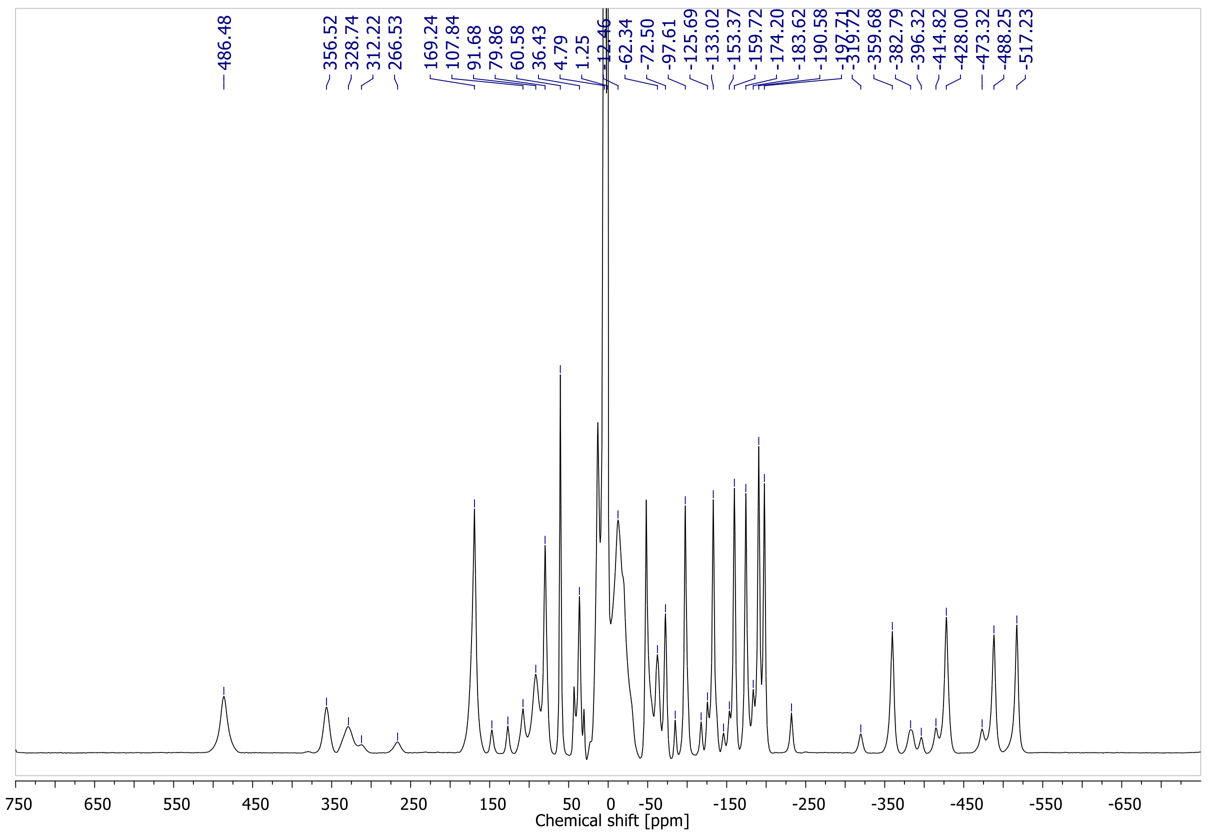

*Figure S7: ^{1}H NMR spectrum of **3·Tb₂** (500 MHz, D_2O).*

4·Eu$_2$. Yield: 35 mg (quant.); t_R= 4.33 min (CN col., 0.1% HCOOH); HR-ESI-MS obsd 1124.2194, calcd 1124.2247 [(M + H)$^+$, M = $C_{36}H_{53}N_9O_{13}Eu_2$].

4·Tb$_2$. Yield: 71 mg (quant.); t_R= 4.24 min (CN col., 0.1% HCOOH); HR-ESI-MS obsd 1138.2320, calcd 1138.2343 [(M + H)$^+$, M = $C_{36}H_{53}N_9O_{13}Tb_2$].

4·Gd$_2$. Yield: 29 mg (quant.); t_R= 4.28 min (CN col., 0.1% HCOOH); HR-ESI-MS obsd 1136.2304, calcd 1136.2318 [(M + H)$^+$, M = $C_{36}H_{53}N_9O_{13}Gd_2$].

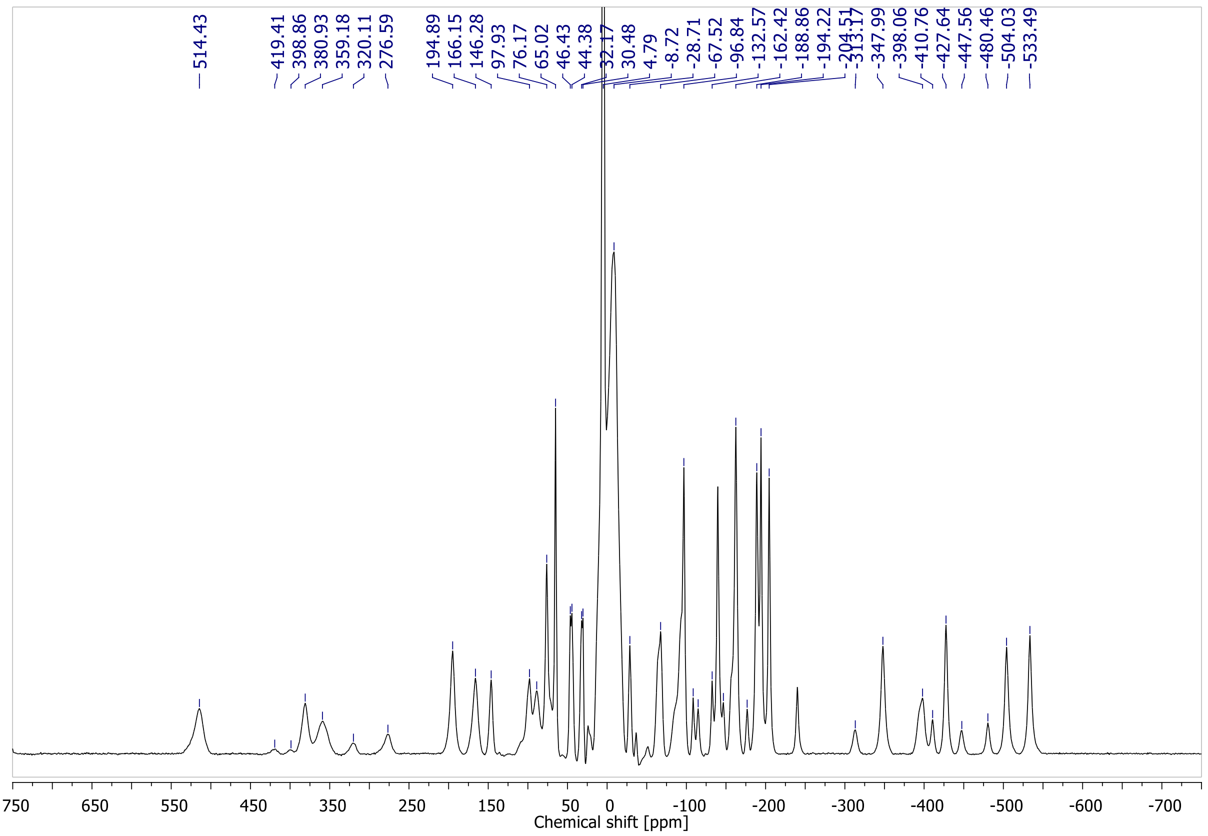

*Figure S8: ^{1}H NMR spectrum of **4·Tb₂** (500 MHz, D_2O).*

A suitable ^{1}H NMR spectrum of **2·Tb₂** could not be obtained due to solubility and phasing issues.

HPLC spectra of Ln(III) complexes:

All HPLC analysis were carried out on a Thermo Scientific Vanquish Core HPLC on an analytical Discovery® Cyano 25 cm × 4.6 mm, 5µm column fitted with a Discovery® Cyano 2 cm × 4.0 mm, 5µm guard column. All samples were filtered using a fisherbrand PTFE filter with 0.2um pore size. Unless otherwise stated HPLC traces were monitored at 272 nm. Methods are detailed below.

Flow rate: 1 mL/min

Method:

Time (min)	MeCN (0.1% Formic Acid) (%)	H_2O (0.1% Formic Acid) (%)
0.0	2.0	98.0
5.0	2.0	98.0
25.0	100	0
27.0	100	0
29.0	2.0	98.0
30.0	2.0	98.0

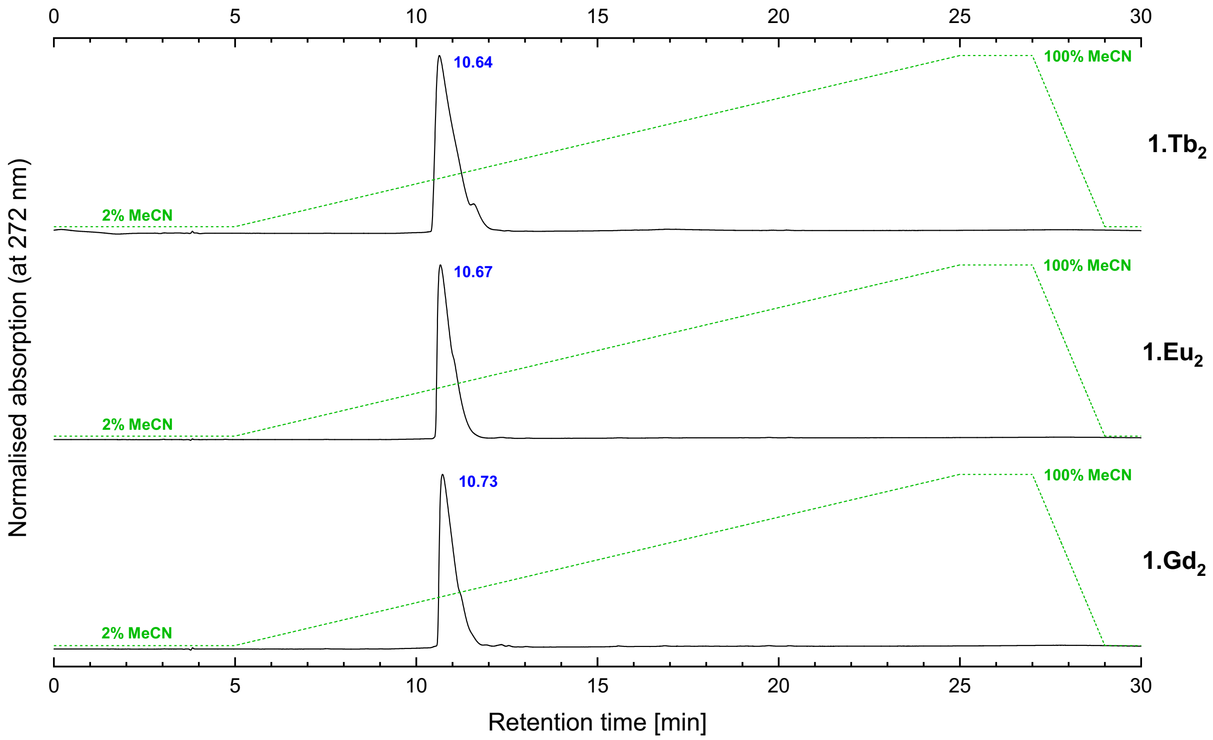

*Figure S9: HPLC traces of complexes **1.Tb$_2$**, **1.Eu$_2$** and **1.Gd$_2$** using method 1.*

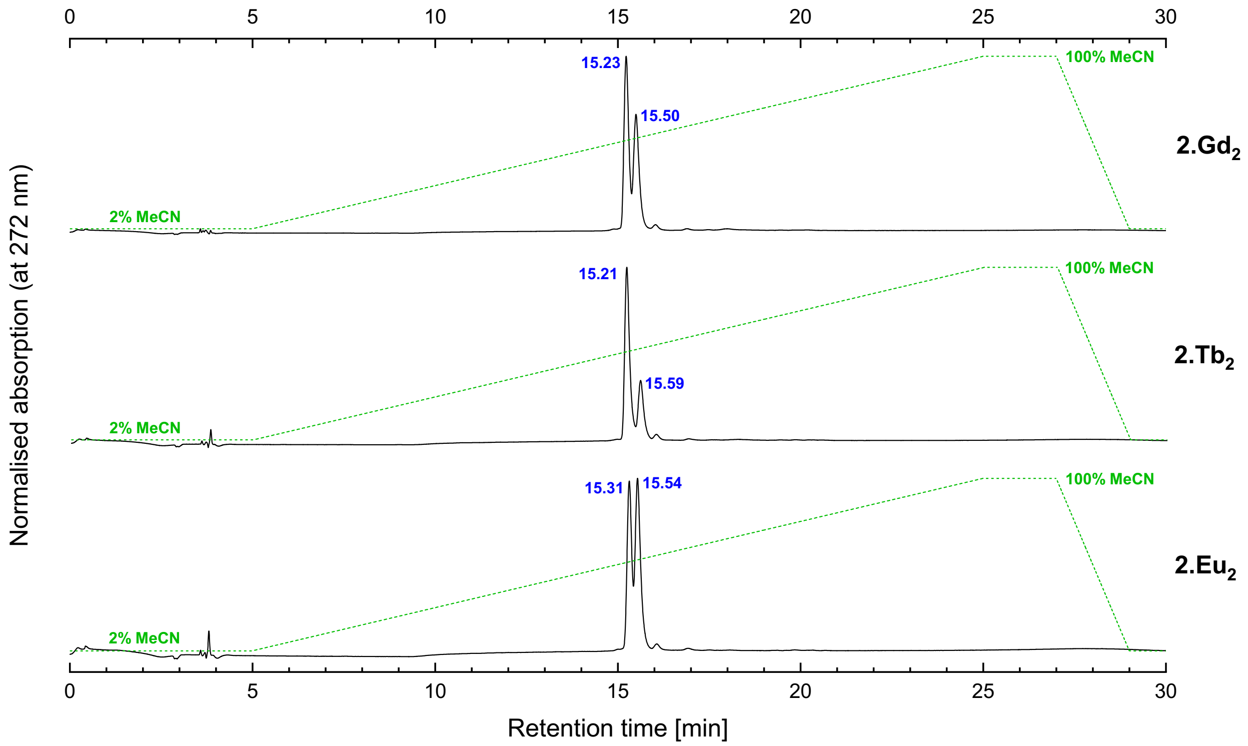

*Figure S10: HPLC traces of complexes **2.Tb₂**, **2.Eu₂** and **2.Gd₂** using method 1, the secondary peaks represent E/Z isomers.*

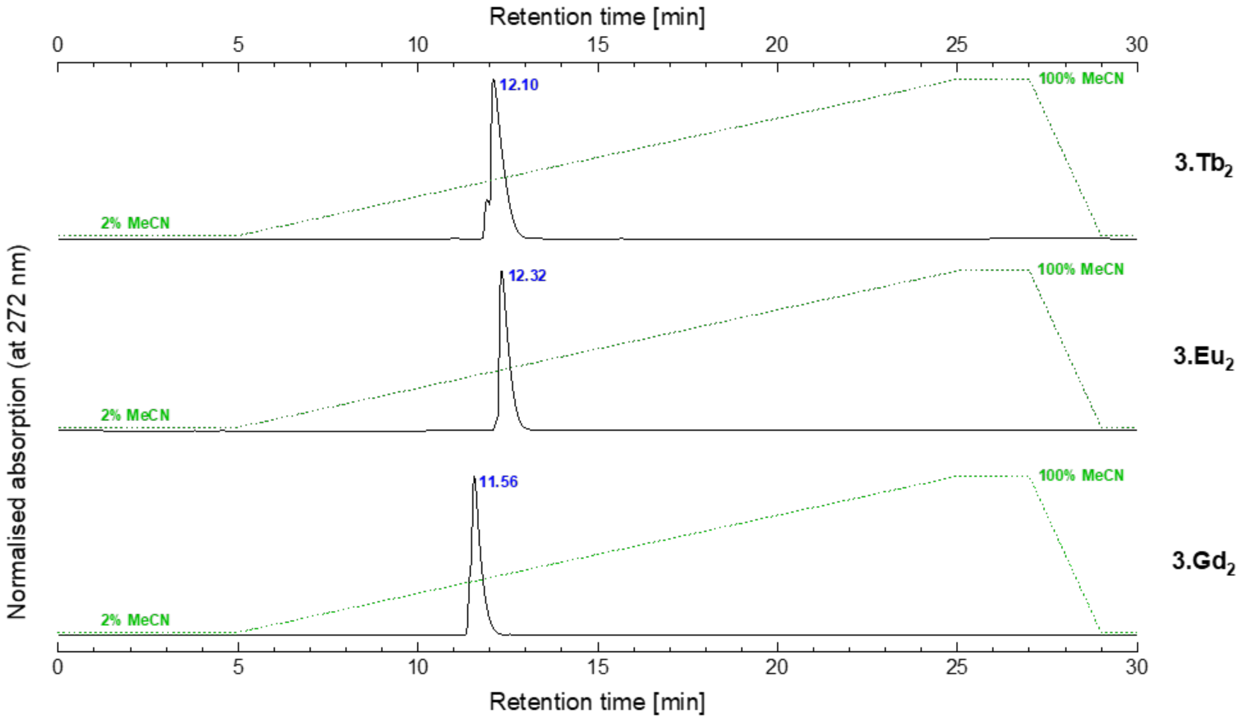

*Figure S11:HPLC traces of complexes **3.Tb₂**, **3.Eu₂** and **3.Gd₂** using method 1.*

Optical Properties

Steady State fluorescence

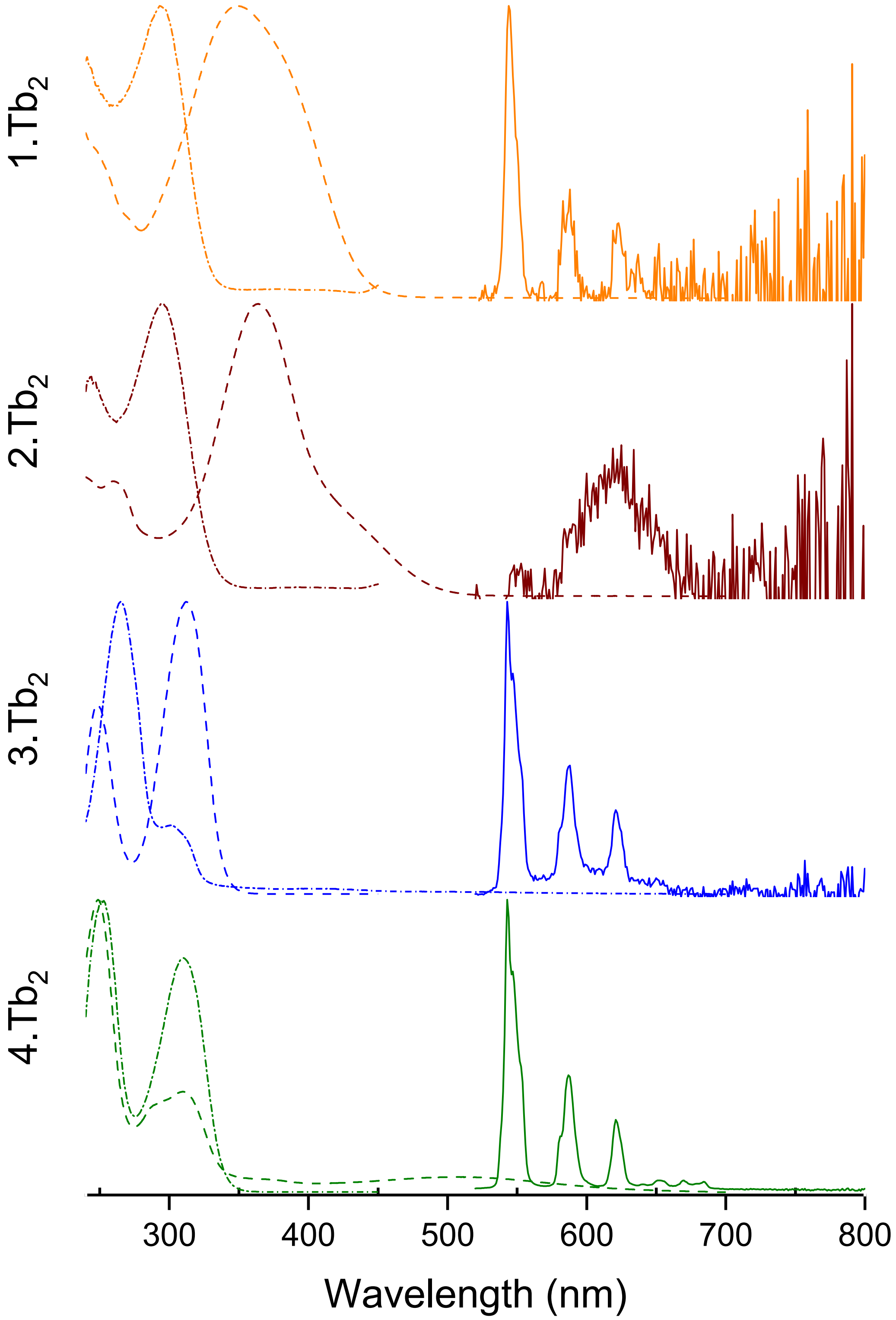

*Figure S12: Comparison of photophysical properties of compounds **1-4·Tb₂**. Complexes were measured at 90 μM in 10 mM PBS buffer at pH 7.4 at 90 μM in 10 mM PBS buffer at pH 7.4 where short dashed line represents the absorbance, dashed line represents the excitation (λ_{em} = 545 nm), and the solid line represents the emission (λ_{ex} = 488 nm).*

Lifetimes

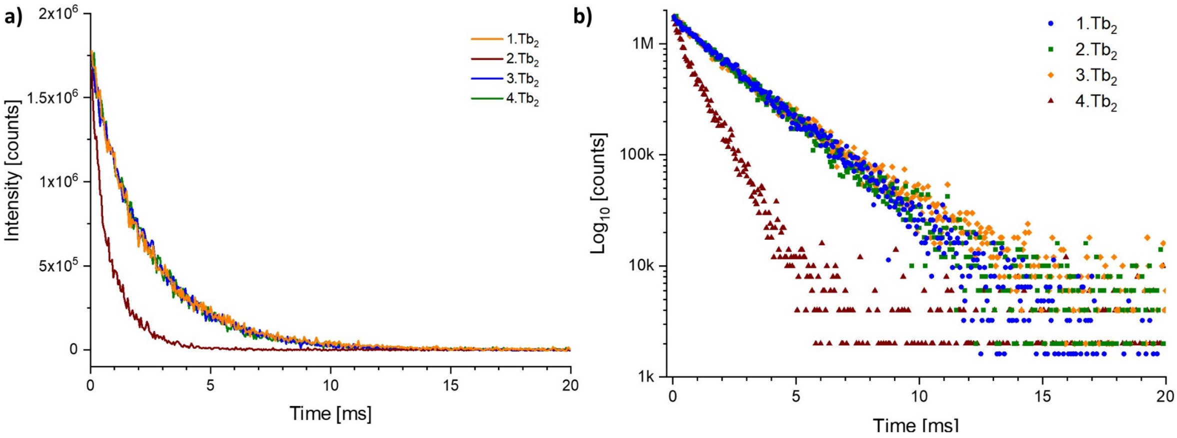

*Figure S13: a) Exponential decays of the complexes **1-4·Tb₂** b) Decay profile on log scale of the complexes **1-4·Tb₂**. Complexes were measured at 90 µM in 10 mM PBS buffer at pH 7.4 at 90 µM in 10 mM PBS buffer at pH 7.4 (orange – **1·Tb₂**, λ_{exc} = 294 nm, λ_{em} = 545 nm, Front slit: 29.00 nm, Exit slit: 14.50 nm, t_{int} = 0.1 s, Sample window = 0.50, Time per flash 61.00, FC = 12, time delay: 50 µs; maroon - **2·Tb₂**, λ_{exc} = 294 nm, λ_{em} = 545 nm, Front slit: 19.0 nm, Exit slit: 6.00 nm, t_{int} = 0.1 s, Sample window = 0.50, Time per flash 61.00, FC = 4, time delay: 50 µs; blue - **3·Tb₂**, λ_{exc} = 310 nm, λ_{em} = 545 nm, Front slit: 13.0 nm, Exit slit: 1.00 nm, t_{int} = 0.1 s, Sample window = 0.50, Time per flash 61.00, FC = 3, time delay: 50 µs; green - **4·Tb₂**, λ_{exc} = 294 nm, λ_{em} = 545 nm, Front slit: 29.00 nm, Exit slit: 2.00 nm, t_{int} = 0.1 s, Sample window = 0.50, Time per flash 61.00, FC = 4, time delay: 50 µs).*

The luminescence lifetime of each complex in D_2O (pD 7.4 (pH + 0.45)) was also measured and the corresponding *q* values (number of bound solvent) were calculated using the modified Horrocks equation (3.3). Here, A = 5.0 and B = 0.06 in water and k_H and k_D (in ms^{-1}) correspond to the rate constant of the lifetime decay in the given solvent and the corresponding deuterated solvent.[68]

$$q = A(k_H - k_D - B)$$

Quantum Yields

The quantum yield of **4.Tb₂** was measured upon excitation at 313 nm, in comparison to a standard Quinine Sulphate.

$$\Phi_s = \frac{I_s}{I_{ref}} \cdot \frac{f_{A_{ref}}}{f_{A_s}} \cdot \frac{(n_s)^2}{(n_{ref})^2} \Phi_{ref}$$

Where the second term is disregarded since the absorption of both complex and reference is identical and the third term removed as both the reference and complex are in dilute conditions and so the refractive index is assumed to be equal leaving:

$$\Phi_s = \frac{I_s}{I_{ref}} \cdot \Phi_{ref}$$

$$\Phi_{s(ligand)} = \frac{1}{107\ 168\ 976{,}10732} \cdot 0.59 \qquad \Phi_{s(fém)} = \frac{81\ 637\ 017{,}668209}{107\ 168\ 976{,}10732} \cdot 0.59$$

$$\Phi_{s(ligand)} = 0.0 \qquad \Phi_{s(fém)} = 0.45248$$

$$\Phi_{s(ligand)} = 0.0\ \% \qquad \Phi_{s(fém)} = 45.2\ \%$$

Triplet Energies

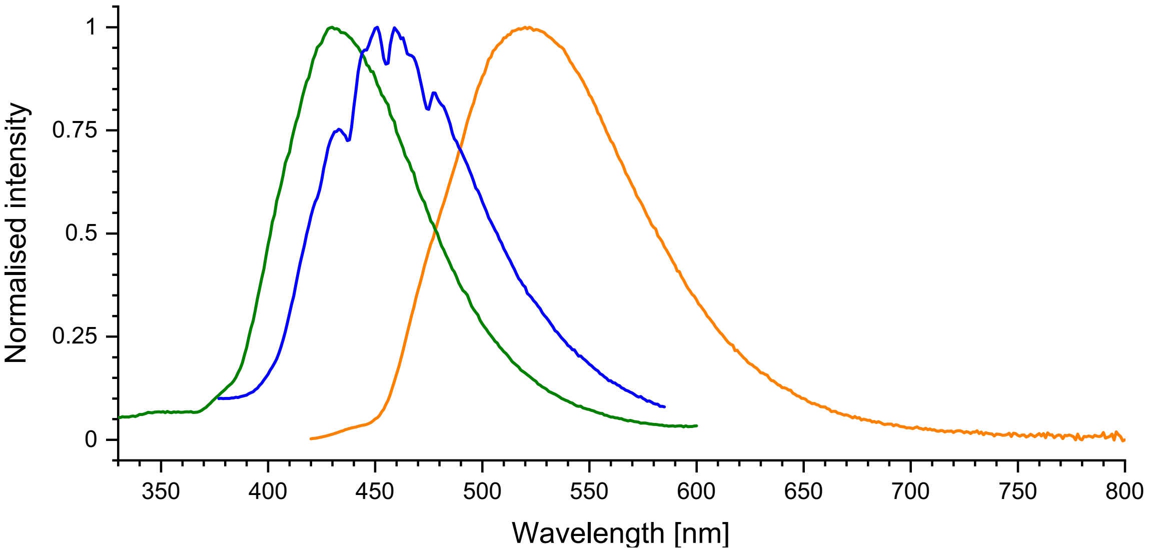

*Figure S14: Normalised steady-state phosphorescence emission of the binuclear Gd complexes (orange – **1·Gd₂**, λ_{exc} = 320 nm, Front slit: 5.00 nm, Exit slit: 0.50 nm, t_{int} = 0.5 s , green – **3·Gd₂**,, λ_{exc} = 310 nm, Front slit: 3.00 nm, Exit slit: 1.00 nm, t_{int} = 0.2 s, blue – **4·Gd₂**,, λ_{exc} = 310 nm, Front slit: 3.00 nm, Exit slit: 1.00 nm, t_{int} = 0.5 s) at 77K measured in 10 mM PBS solution at pH 7.4, with 10% glycerol added. Complex concentrations were at 90 uM. **2·Gd₂**, could not be measured due to very low lying excited states.*

X-ray Crystal Structure

Single crystal X-ray structures reported in this work were obtained by using the vapour diffusion method. The sample were dissolved in a minimal amount of water and acetone was used as an antisolvent.

For **5·Eu$_2$** low temperature single crystal X-ray diffraction data were collected using a (Rigaku) Oxford Diffraction SuperNova diffractometer. Raw frame data were reduced using CrysAlisPro and the structure was solved using 'Superflip' [L. Palatinus and G. Chapuis, J. Appl. Cryst., 2007, 40, 786-790.] before refinement with CRYSTALS [(a) P. Parois, R.I. Cooper and A.L. Thompson, Chem. Cent. J., 2015, 9:30. (b) R.I. Cooper, A.L. Thompson and D.J. Watkin, J. Appl. Cryst. 2010, 43, 1100-1107.] as per the SI (CIF). Crystallographic data have been deposited with the Cambridge Crystallographic Data Centre (CCDC 2370191) and copies of these data can be obtained free of charge via www.ccdc.cam.ac.uk/data_request/cif.

Crystal system / Space group	Monoclinic /	C 2/c
Unit cell dimensions	a = 36.7160(3) Å	α= 90°.
	b = 16.52100(10) Å	β= 101.0464(9)°.
	c = 17.7958(2) Å	γ = 90°.
Volume	10594.67(16) Å^3	
Z	4	
Crystal size	0.25 x 0.07 x 0.05 mm^3	
Temperature	150 K	
Wavelength	1.54184 Å	
Independent reflections	11102 [R(int) = 0.059]	
Data / restraints / parameters	11096 / 0 / 614	
Goodness-of-fit on F^2	0.9335	
Final R indices [I>2sigma(I)]	R1 = 0.0893, wR2 = 0.2262	
R indices (all data)	R1 = 0.0910, wR2 = 0.2317	

a)

b)

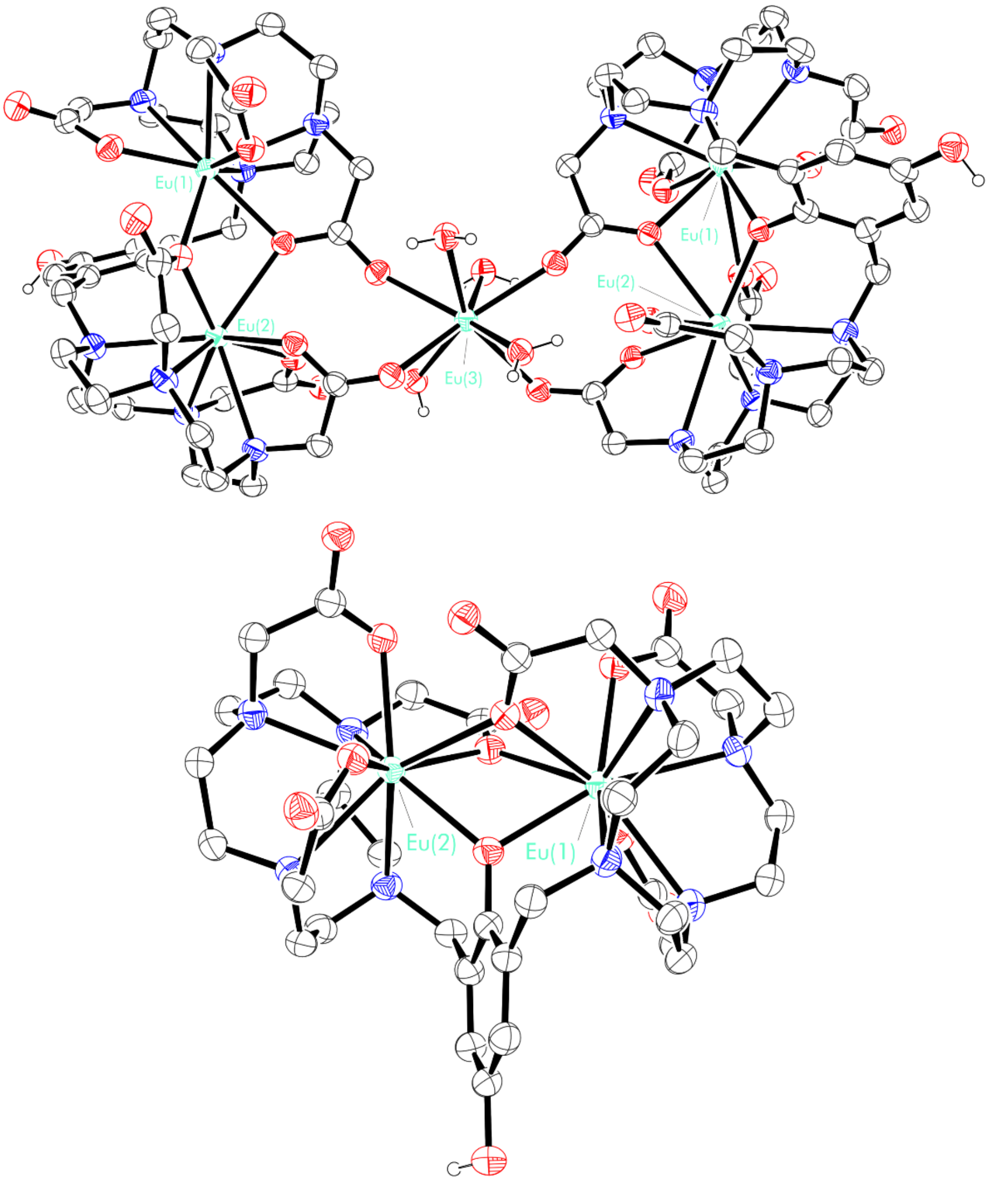

*Figure S15: Thermal displacement ellipsoid drawing (50% probability) of a) the full structure of the [[**5·Eu$_2$**]$_2$[Eu(H_2O)$_4$]]$^-$ anion, which co-crystallises with a hydrated Na^+ cation (not shown) and b) **5·Eu$_2$**. All H atoms (excluding OH protons) and non-coordinated solvent molecules have been omitted for clarity.*

Table S1. *Experimental metrical parameters (bond lengths in Å and angles in °) in* ***5·Eu₂***.

Eu(1)-Eu(31)	3.7698(5)
Eu(1)-O(22)	2.376(5)
Eu(1)-O(48)	2.629(5)
Eu(1)-O(56)	2.345(4)
Eu(1)-O(56)-Eu(31)	107.7(2)
Eu(1)-O(48)-Eu(31)	97.9(2)
Eu(1)-O(22)-Eu(31)	99.3(2)
Eu(1)-O(14)	2.394(5)
Eu(1)-O(18)	2.318(5)
Eu(1)-O(56)	1.7141(9)
Eu(1)-N(11)	2.785(5)
Eu(1)-N(5)	2.648(5)
Eu(1)-N(2)	2.650(6)
Eu(1)-N(8)	2.718(6)
Eu(31)-O(56)	2.324(5)
Eu(31)-O(48)	2.364(5)
Eu(31)-O(22)	2.570(5)
Eu(31)-O(52)	2.385(6)
Eu(31)-O(44)	2.376(5)
Eu(31)-N(38)	2.651(5)
Eu(31)-N(35)	2.699(5)
Eu(31)-N(41)	2.765(6)
Eu(31)-N(32)	2.618(6)

Relaxivity

Note: **2·Gd₂** was not fully soluble in PBS.

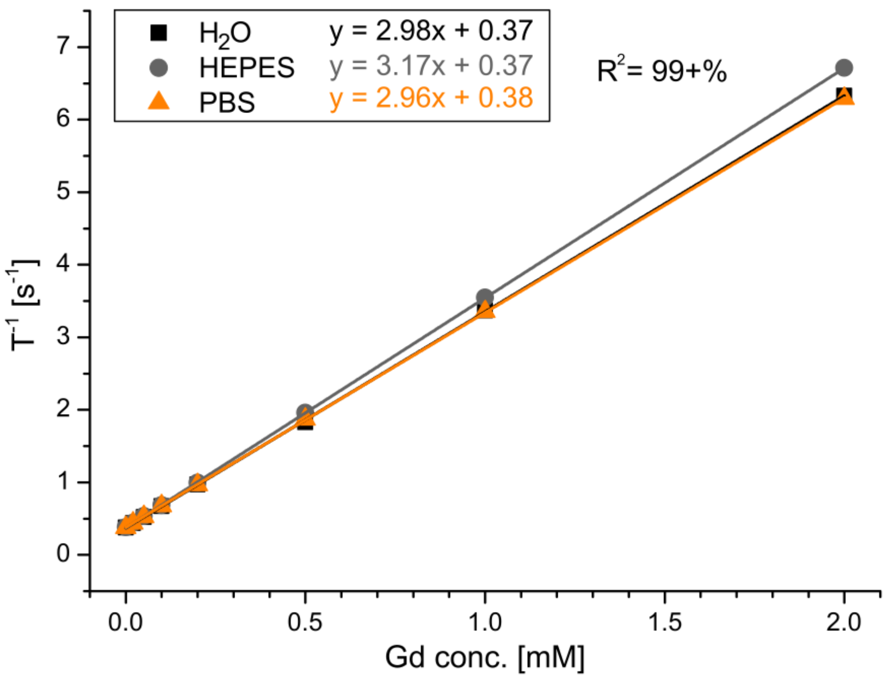

*Figure S16: Effect of buffer on T1 relaxivities. Variation of 1/T1 with concentration of **1·Gd$_2$** dissolved in different buffers (at 20 °C, 7 T).*

Cyclic Voltammetry (CV)

The small-volume electrochemical cell consisted of a polyether ether ketone (PEEK) cylinder containing a working electrode of glassy carbon (1 mm diameter), a counter electrode of graphite, and a leak-free Ag/AgCl reference electrode (LF-2-45 model from Alvatek Ltd). The cell was set up under a N_2 atmosphere in a glove box (O_2 < 2 ppm), using 400 µL of a 1 mM solution of complex dissolved in degassed H_2O containing 50 mM Tris and 50 mM NaCl at pH 7.4 or 100 mM MES and 50 mM NaCl at pH 6.0. The air-tight electrochemical cell was then removed from the glove box and connected to an AutoLab 128 N potentiostat (Metrohm) controlled by Nova 2.1.7 software. Cyclic voltammograms were run with step potential = -0.00244 V and scan rate = 0.02 V/s, with 3 cycles recorded for each. The Ag/AgCl reference electrode was calibrated to vs SHE by measuring the cyclic voltammogram of FcMeOH (0.1 mM in 4:1 buffer:EtOH) and comparing to a literature $E_{1/2}$ value of +420 mV vs SHE for FcMeOH.[7] Cathodic onset potentials were calculated as follows: the first linear sweep of the voltammogram of the blank (buffer only) was subtracted from the first linear sweep of the voltammogram of the substrate; a linear baseline in a region of no electrochemical activity was extrapolated across the entire current range; the cathodic onset potential is taken as the potential at which the current in the baseline-corrected voltammogram begins to increase exponentially and exceeds a threshold value of 10 nA (instrument current resolution 0.0003% of current range). This procedure is adapted from a method used to define onset potentials for a range of nitro-compounds from the current at a rotating disk electrode which is typically higher in magnitude, and hence the data from a stationary electrode obtained here are not fully comparable to the earlier data set.[8] A stationary electrode was used here due to the smaller sample requirement in the cell.

*Table S 2: Cathodic onset potentials vs SHE (V) of **1-3·Tb₂**.*

	**Cathodic Onset Potential vs SHE / V**	
	**pH 7.4** (Tris)	**pH 6.0** (MES)
**1·Tb_2**	-0.413	-0.240
**2·Tb_2**	-0.394	-0.258
**3·Tb_2**	/	/

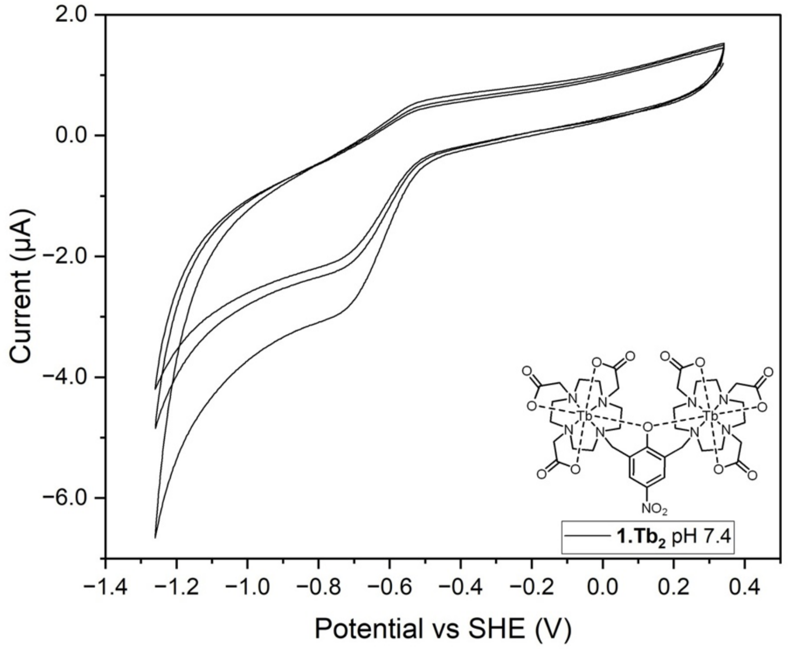

*Figure S17: CV of **1.Tb₂** in 50 mM Tris 50 mM NaCl H_2O at pH 7.4, showing 3 cycles. The reduction wave is assigned to the nitro functional group.*

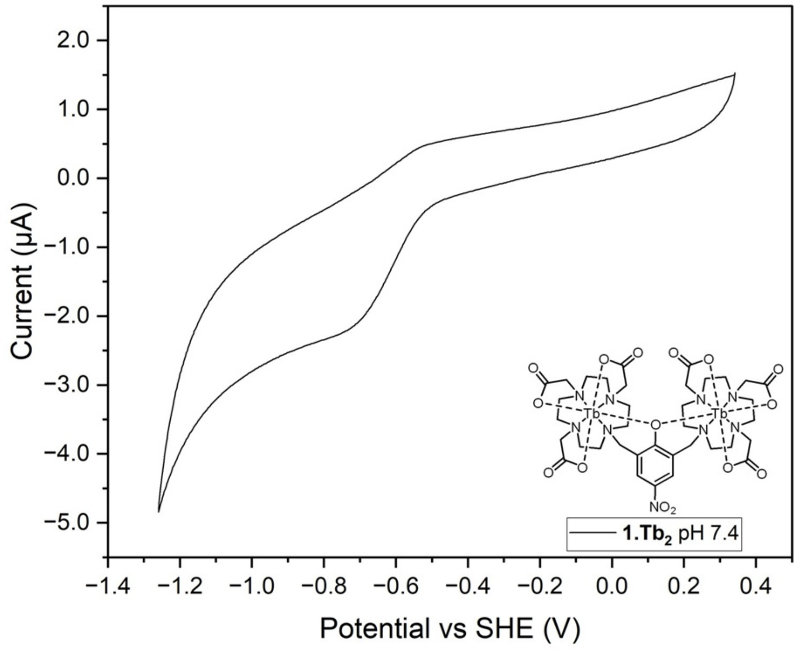

*Figure S18: CV of **1.Tb₂** in 50 mM Tris 50 mM NaCl H_2O at pH 7.4, showing the second of 3 cycles. The reduction wave is assigned to the nitro functional group.*

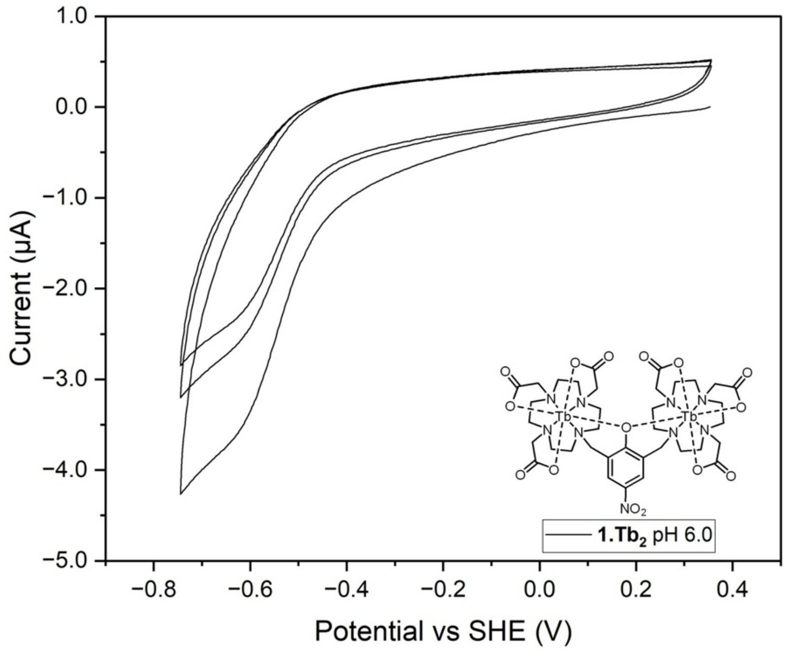

*Figure S19: CV of **1.Tb₂** in 100 mM MES 50 mM NaCl H_2O at pH 6.0, showing 3 cycles. The cathodic wave is assigned to reduction of the nitro functional group.*

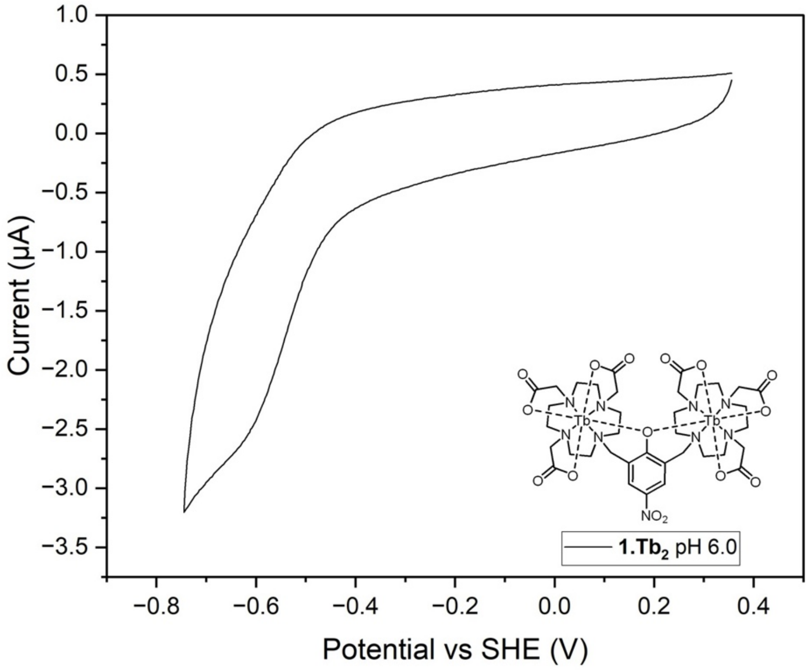

*Figure S20: CV of **1.Tb₂** in 100 mM MES 50 mM NaCl H_2O at pH 6.0, showing the second of 3 cycles. The cathodic wave is assigned to reduction of the nitro functional group.*

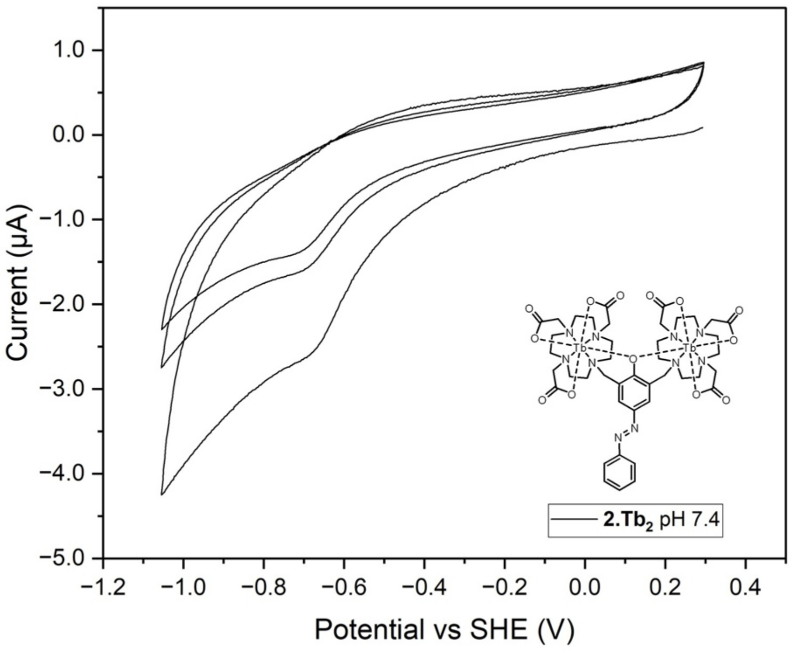

*Figure S21: CV of **2.Tb₂** in 50 mM Tris 50 mM NaCl H_2O at pH 7.4, showing 3 cycles. The cathodic wave is assigned to reduction of the azo functional group.*

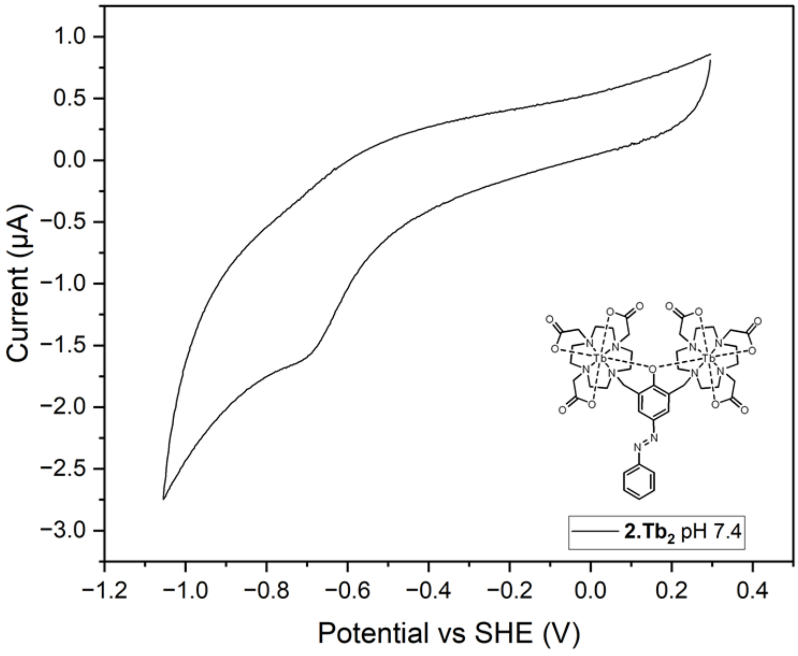

*Figure S22: CV of **2.Tb₂** in 50 mM Tris 50 mM NaCl H_2O at pH 7.4, showing the second of 3 cycles. The cathodic wave is assigned to reduction of the azo functional group.*

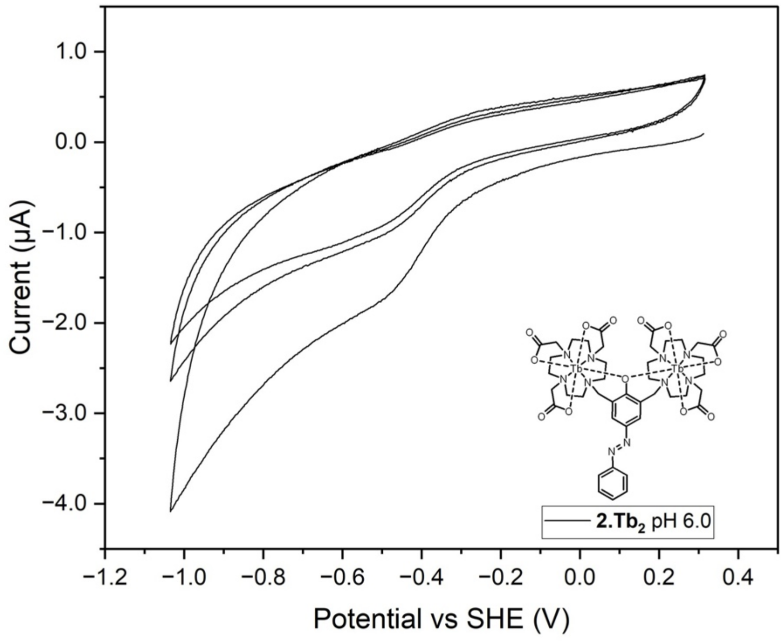

*Figure S23: CV of **2.Tb₂** in 100 mM MES 50 mM NaCl H_2O at pH 6.0, showing 3 cycles. The cathodic wave is assigned to reduction of the azo functional group.*

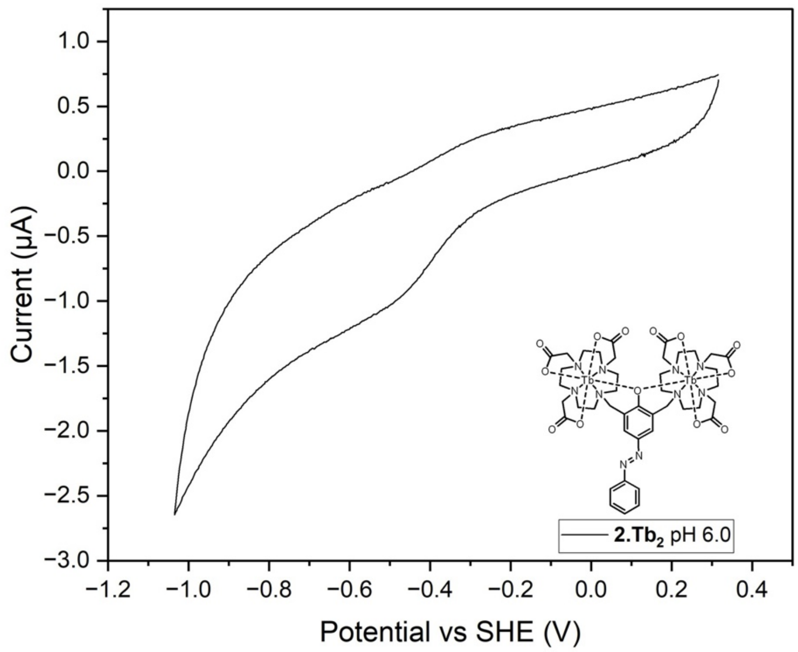

*Figure S24: CV of **2.Tb₂** in 100 mM MES 50 mM NaCl H_2O at pH 6.0, showing the second of 3 cycles. The cathodic wave is assigned to reduction of the azo functional group.*

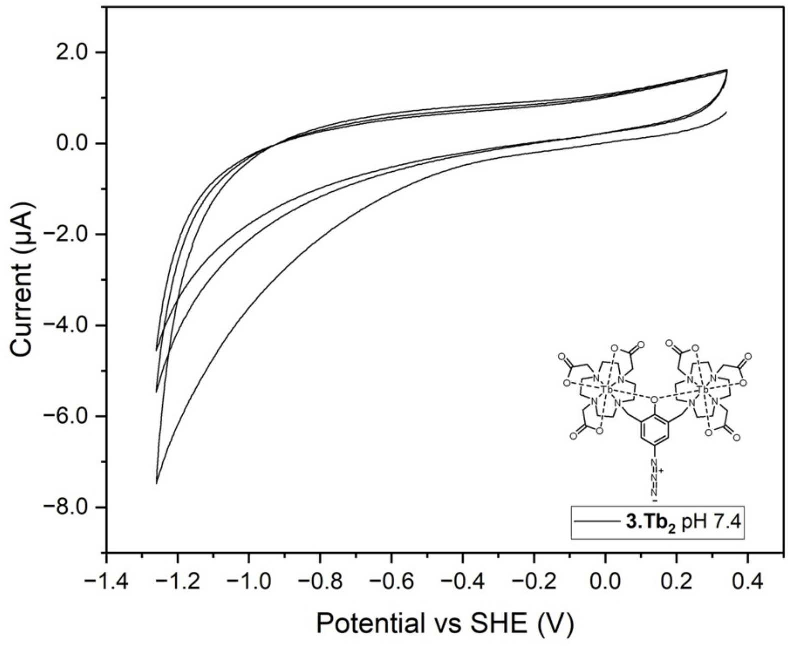

*Figure S25: CV of **3.Tb₂** in 50 mM Tris 50 mM NaCl H_2O at pH 7.4, showing 3 cycles with no obvious electrochemical activity on the substrate*

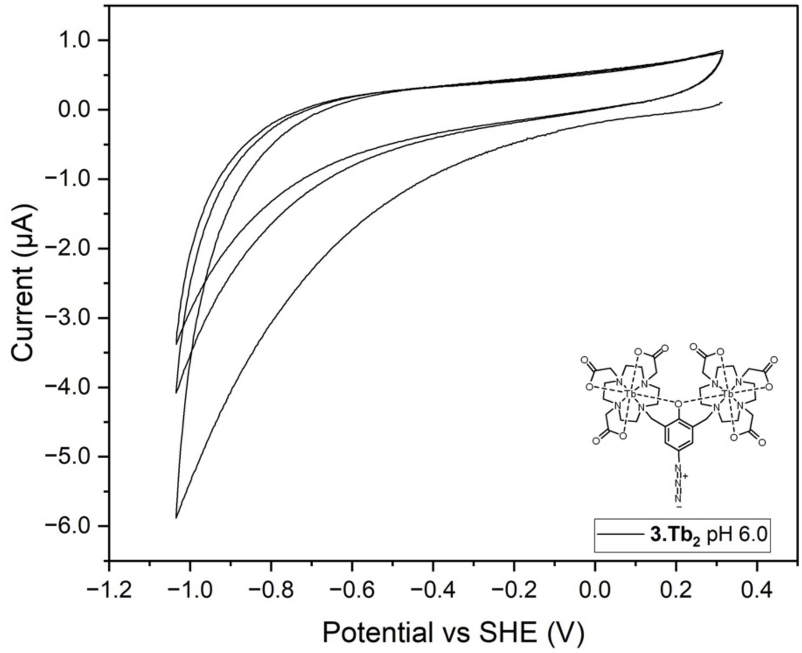

*Figure S26: CV of **3.Tb₂** in 100 mM MES 50 mM NaCl H_2O at pH 6.0, showing 3 cycles with no obvious electrochemical activity on the substrate.*

Chemical reduction of the binuclear Ln(III) probes

Reduction procedure for analysis by ^{1}H NMR:

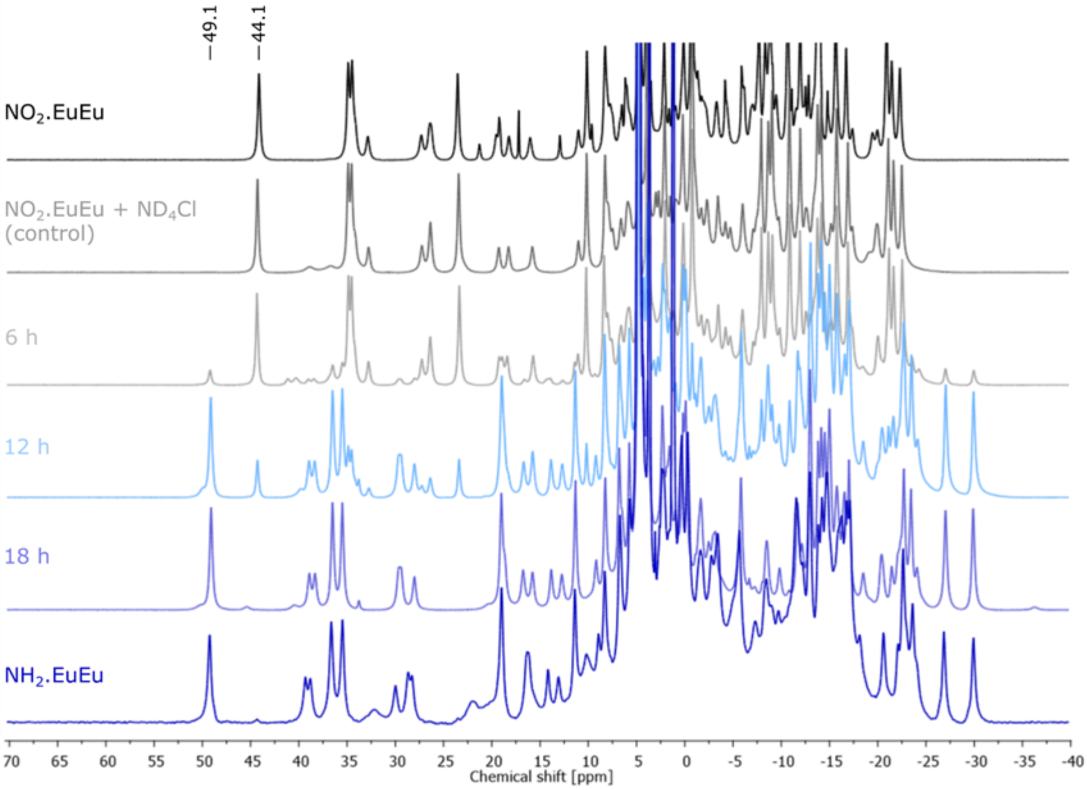

*Figure S27: Stacked ^{1}H NMR spectra of the chemical reduction of **1·Eu₂** with zinc powder and NH_4Cl. Top and bottom spectra are used for referencing and were recorded of the pure complexes in D_2O.*

1·Eu$_2$ (0.0138 mmol) was dissolved in 400 µL D_2O followed by the addition of Zn powder (0.248 mmol). To the stirred mixture NH_4Cl (4.5 µL, 0.0082 mmol (10 m/m% solution in D_2O)) was added and the reaction was let to stir at 37 °C for 24 hours. The reaction mixture was centrifuged and the supernatant was decanted and used directly for NMR experiments. The solvent was removed under continuous N_2 flow at 50 °C to afford the reduced complex as a dark purple solid. HR-ESI-MS obsd 1124.2069, calcd 1124.2115 [(M - H)$^-$, M = $C_{36}H_{53}N_9O_{13}Eu_2$]

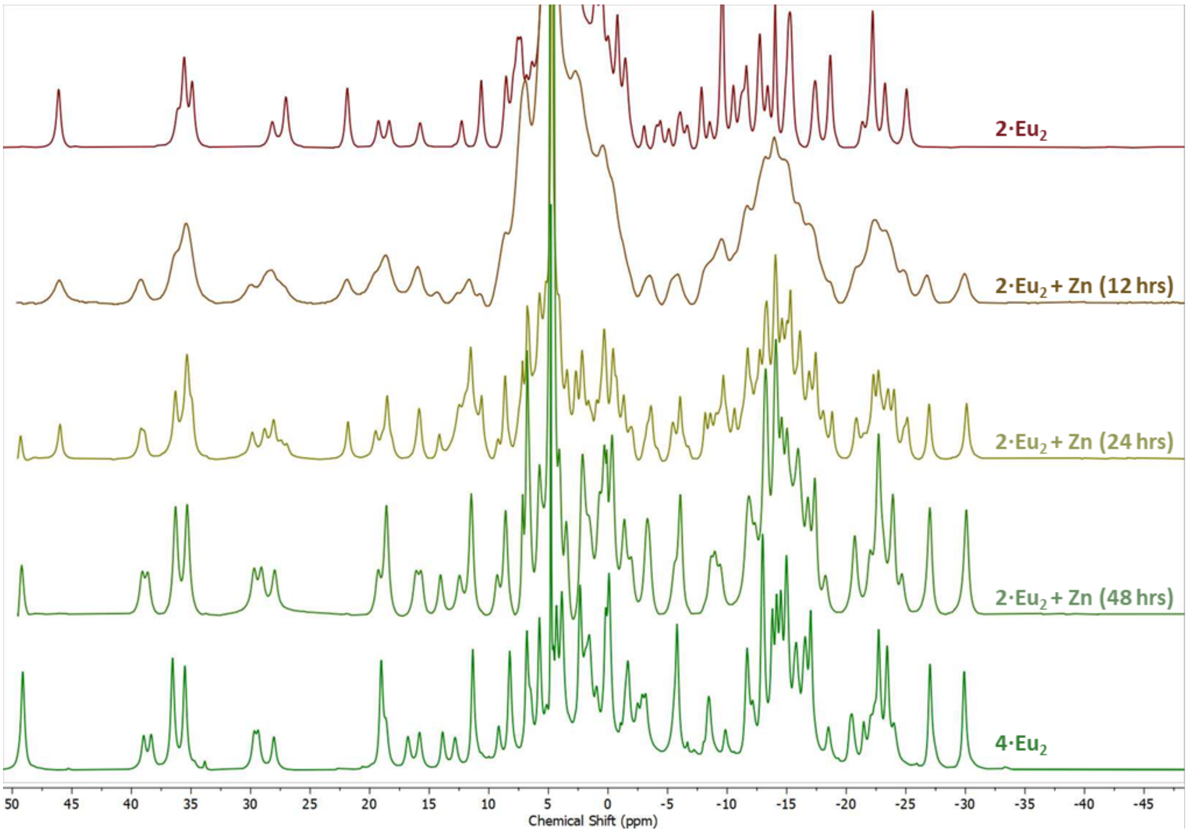

*Figure S28: Stacked ^1H NMR spectra of the chemical reduction of **2·Eu₂** with zinc powder and NH_4Cl. Top and bottom spectra are used for referencing and were recorded of the pure complexes in D_2O.*

$2{\cdot}Eu_2$ (0.0138 mmol) was dissolved in 400 µL D_2O followed by the addition of Zn powder (0.248 mmol). To the stirred mixture NH_4HCO_2 (4.5 µL, 0.0082 mmol (10 m/m% solution in D_2O)) was added with a and the reaction was let to stir at 37 °C for 24 hours. The reaction mixture was centrifuged and the supernatant was decanted and used directly for NMR experiments. The solvent was removed under continuous N_2 flow at 50 °C affording the reduced complex as a dark purple solid. HR-ESI-MS obsd 1126.2298, calcd 1126.2261 [$(M + D)^+$, M = $C_{36}H_{52}N_9O_{13}Eu_2$]

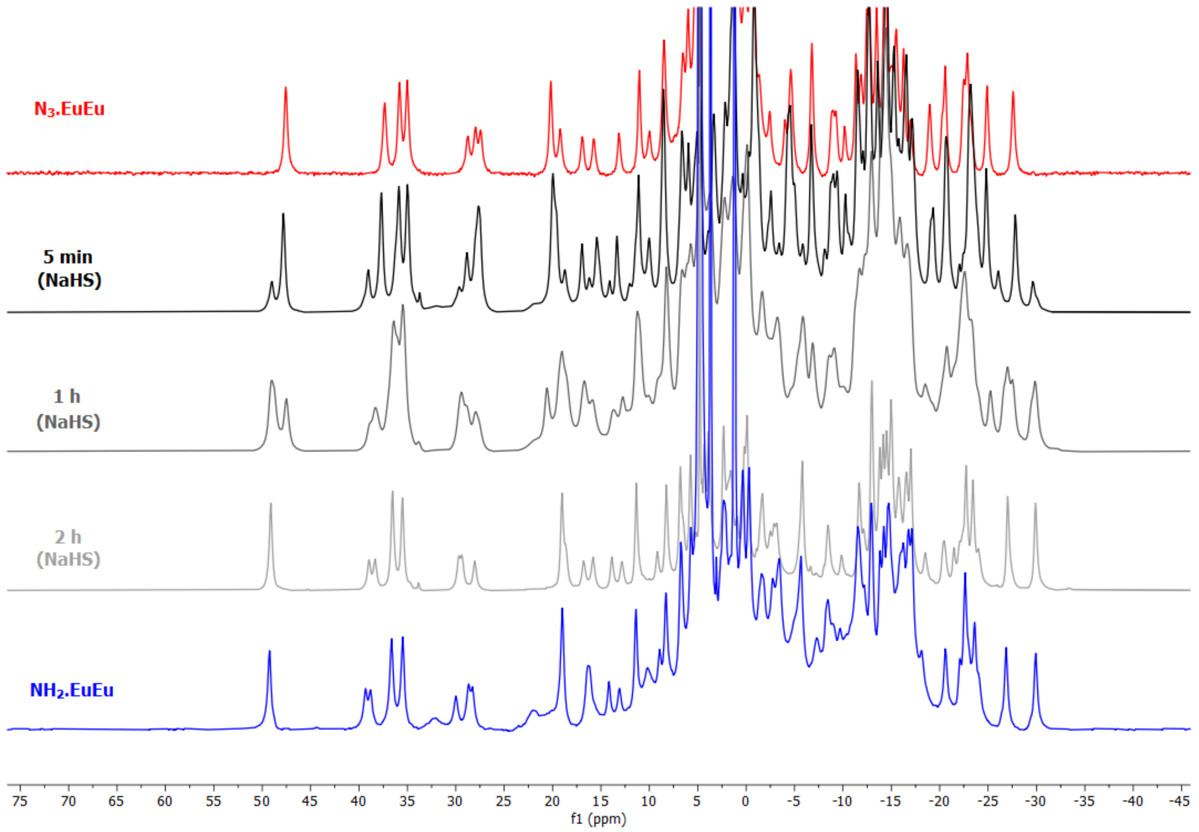

*Figure S29: Stacked ^{1}H NMR spectra of the chemical reduction of **3·Eu₂** with zinc powder and NH_4Cl. Top and bottom spectra are used for referencing and were recorded of the pure complexes in D_2O.*

3·Eu$_2$ (0.0236 mmol, 1.00 eqv) was dissolved in D_2O (280 µL) followed by the addition of NaHS·xH_2O (141 µL, 0.141 mmol (1 M solution in D_2O), 6.00 eqv) with a micropipette and the reaction was let to stir at 37 °C for 2 hours. In the first 30 mins intense effervescence was observed. The reaction mixture was centrifuged and the supernatant was decanted and used directly for NMR experiments. The solvent was removed under continuous N_2 flow at 50 °C to result the reduced complex as a dark purple solid. HR-ESI-MS obsd 1125.1194, calcd 1125.2182 [$(M + H)^+$, M = $C_{36}H_{52}N_9O_{13}Eu_2$]

Reduction procedure for analysis by luminescence (plate reader Asssay):

A Tecan infinite M1000 Pro Plate reader was used to investigate the chemical reduction of probes **1-4·Tb$_2$** in a 96 well plate. To each well 100 µl of sample was added. Varying concentrations of compound were added (0.01 M, 0.001 M and 0.0001 M) in MES buffer (0.1 M pH 6.0). To each sample 10 µl of 1 M Zinc and 20 µl of 1M ammonium formate was added. Control samples had 30 µl of MES buffer pH 6.0 added, a spectrum was recorded every 30 minutes for 16 hours, samples were shaken before each measurement. λ_{ex} = 280 nm, emission slit = 2.5 nm, excitation slit = 2.5 nm, gain = 165, integration time 0.2 ms.

4·Tb$_2$

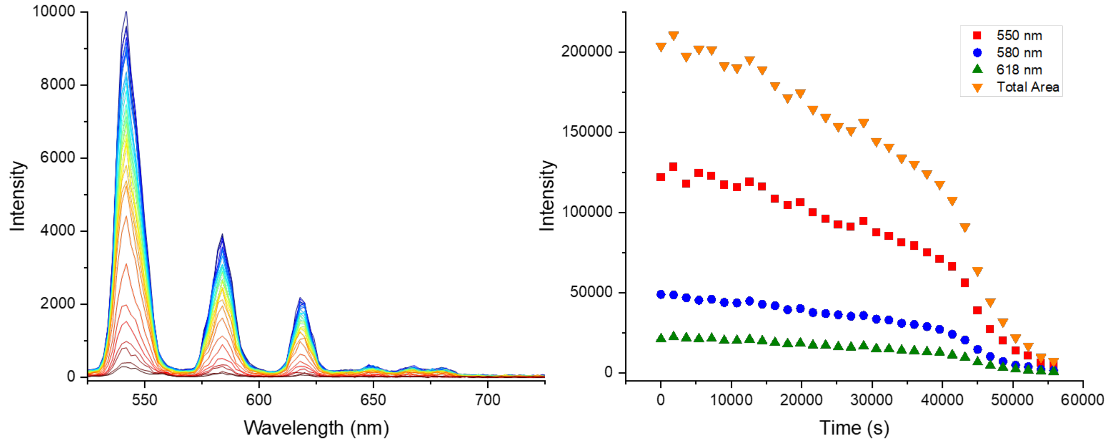

*Figure S30: **4·Tb$_2$** (0.01 M) in MES (0.1 M)*

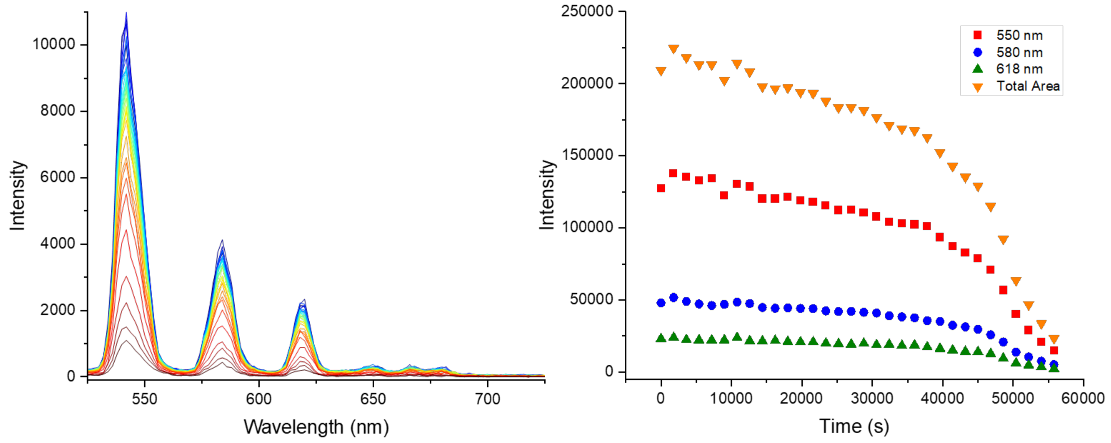

*Figure S31 **4·Tb$_2$** (0.01 M) in MES (0.1 M) with zinc and ammonium formate*

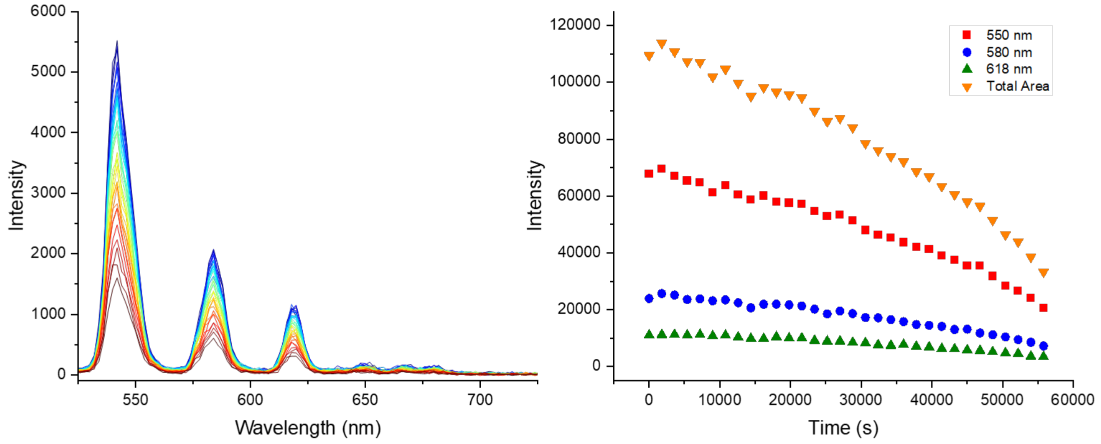

*Figure S32: **4·Tb₂** (0.001 M) in MES (0.1 M)*

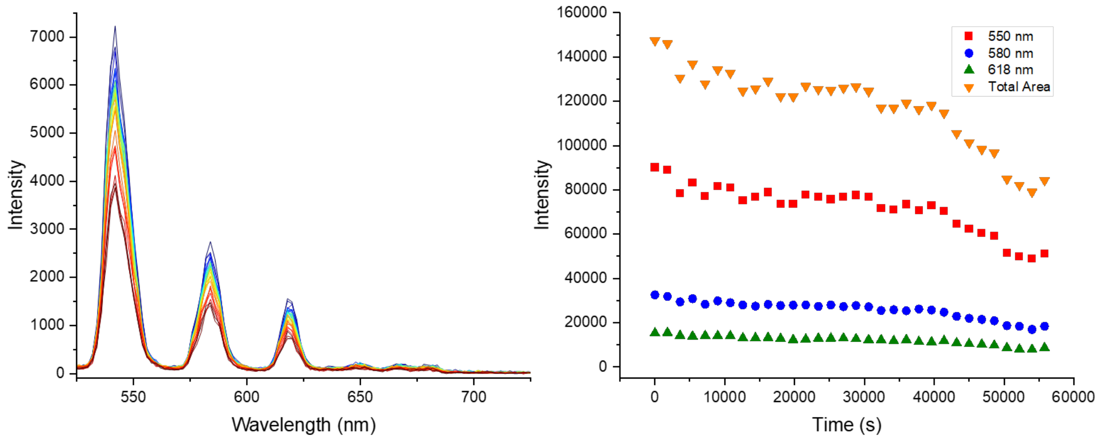

*Figure S33: **4·Tb₂** (0.001 M) in MES (0.1 M) with zinc and ammonium formate*

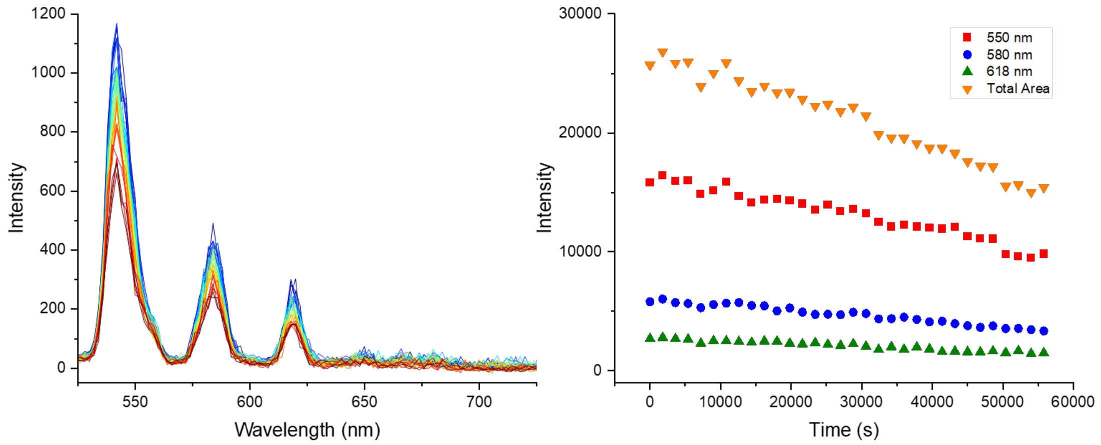

*Figure S34: **4·Tb₂** (0.0001 M) in MES (0.1 M)*

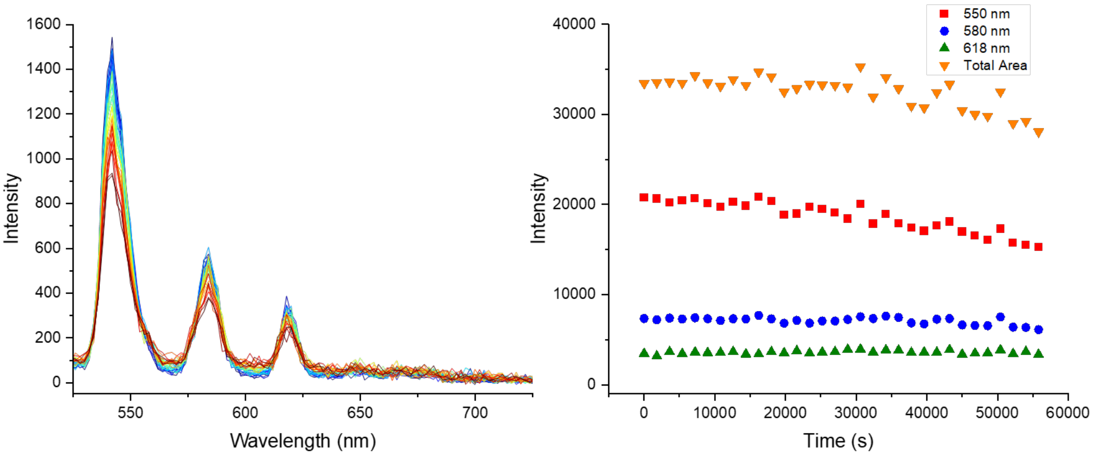

*Figure S35: **4·Tb₂** (0.0001 M) in MES (0.1 M) with zinc and ammonium formate*

1·Tb_2

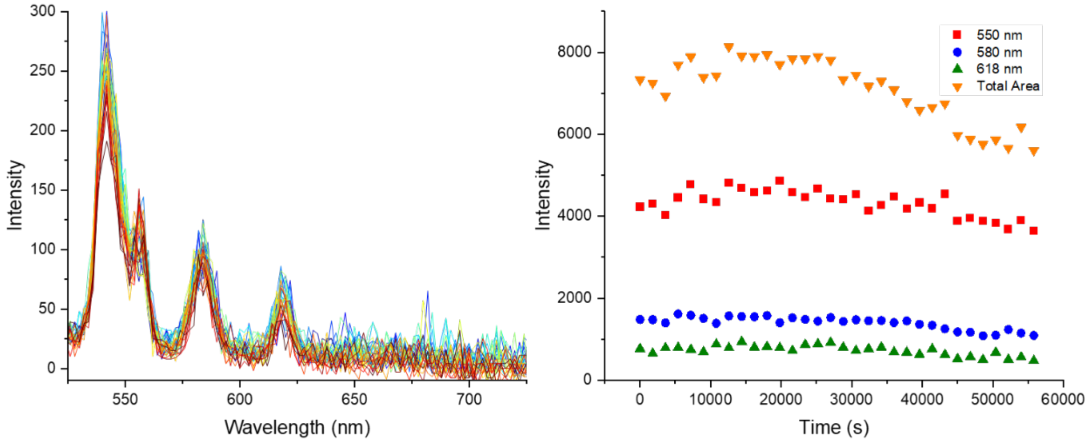

*Figure S36 **1·Tb₂** (0.01 M) in MES (0.1 M)*

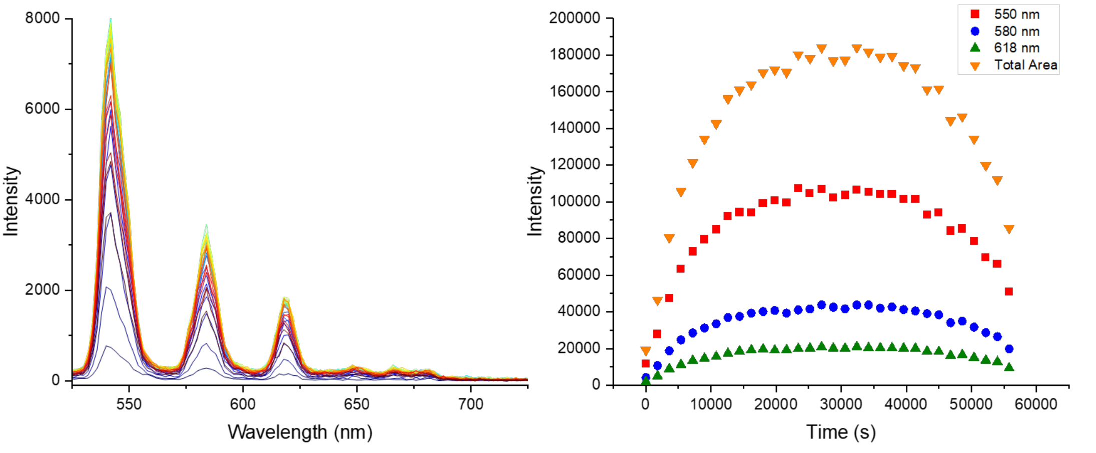

*Figure S37: **1·Tb₂** (0.01 M) in MES (0.1 M) with zinc and ammonium formate*

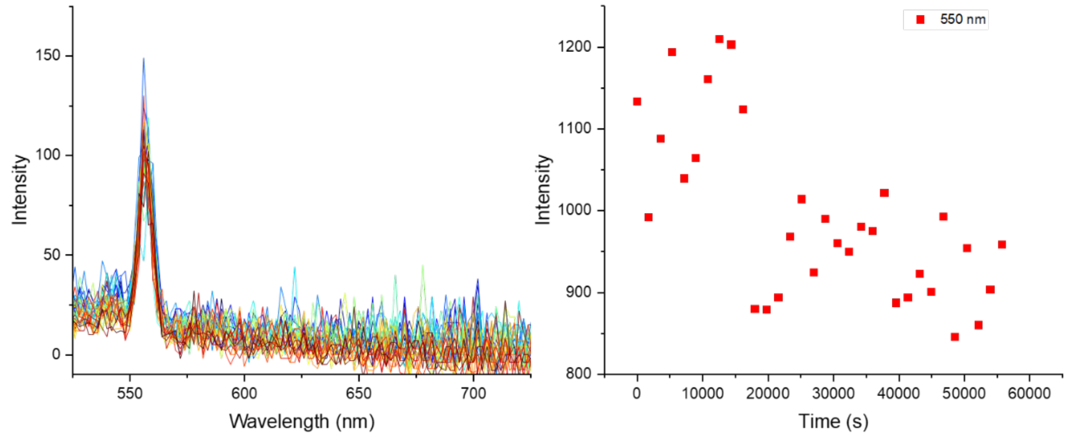

*Figure S38: **1·Tb₂** (0.001 M) in MES (0.1 M)*

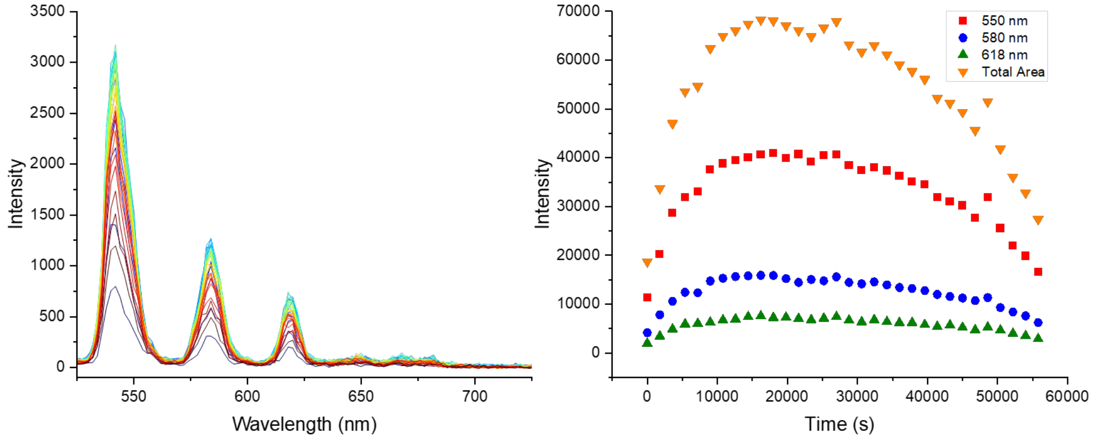

*Figure S39: **1·Tb₂** (0.001 M) in MES (0.1 M) with zinc and ammonium formate*

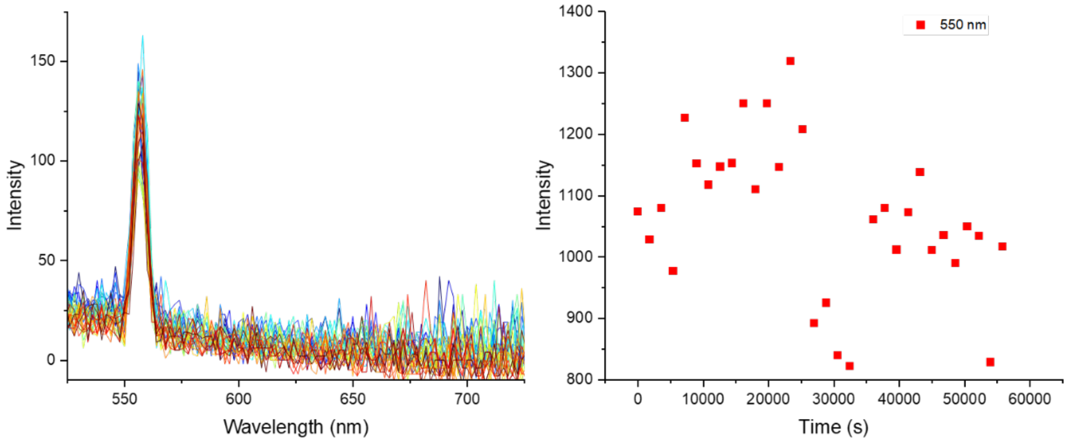

*Figure S40: **1·Tb₂** (0.0001 M) in MES (0.1 M)*

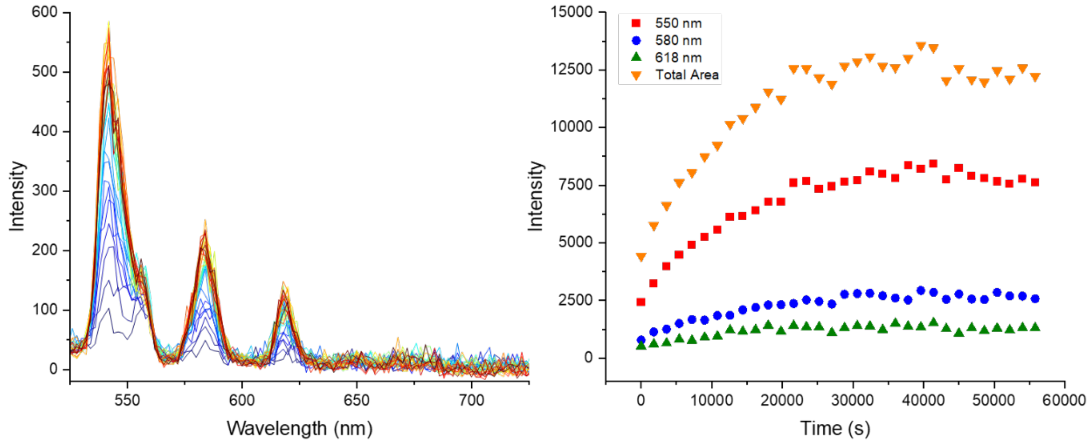

*Figure S41: **1·Tb$_2$** (0.0001 M) in MES (0.1 M) with zinc and ammonium formate*

2·Tb$_2$

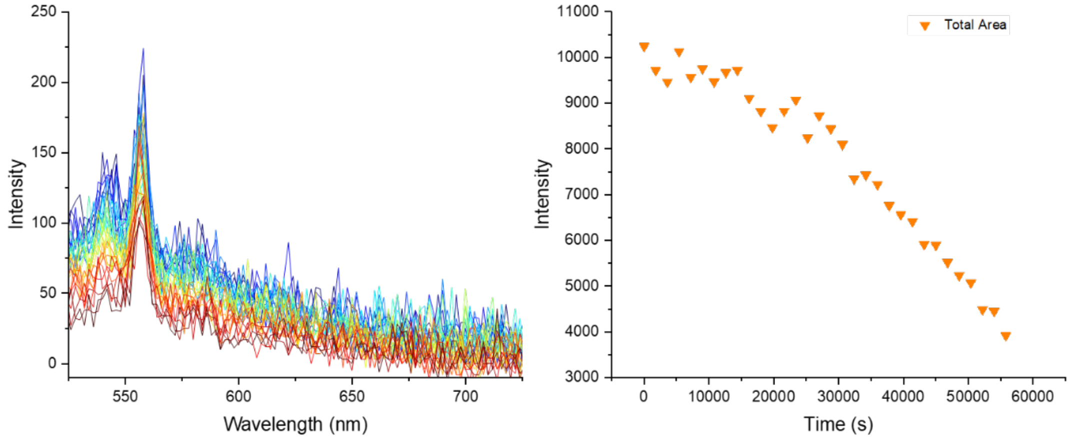

*Figure S42: **2·Tb$_2$** (0.001 M) in MES (0.1 M)*

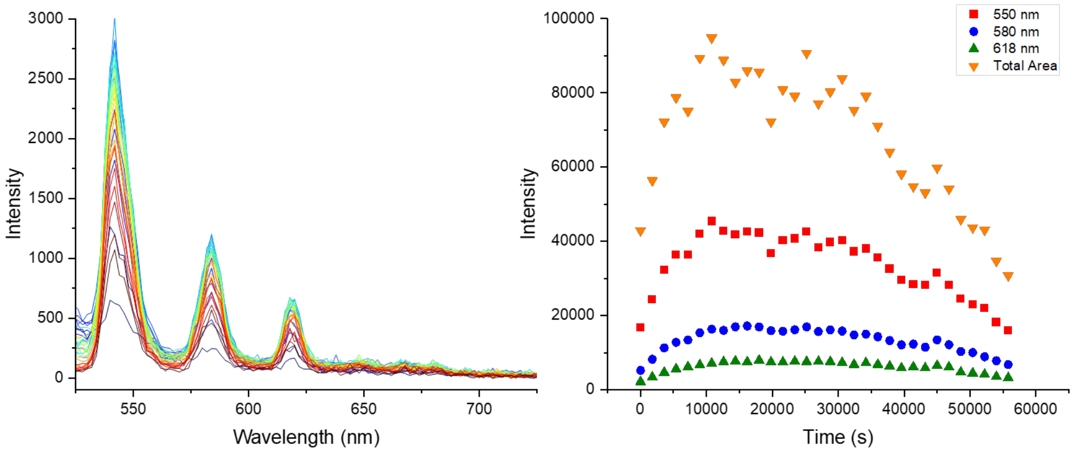

*Figure S43: **2·Tb$_2$** (0.001 M) in MES (0.1 M) with zinc and ammonium formate*

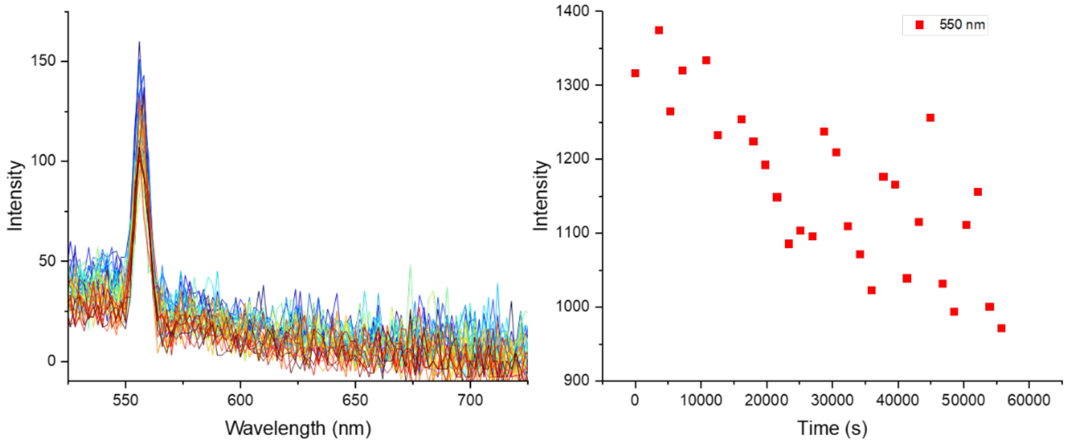

*Figure S44: **2·Tb₂** (0.0001 M) in MES (0.1 M)*

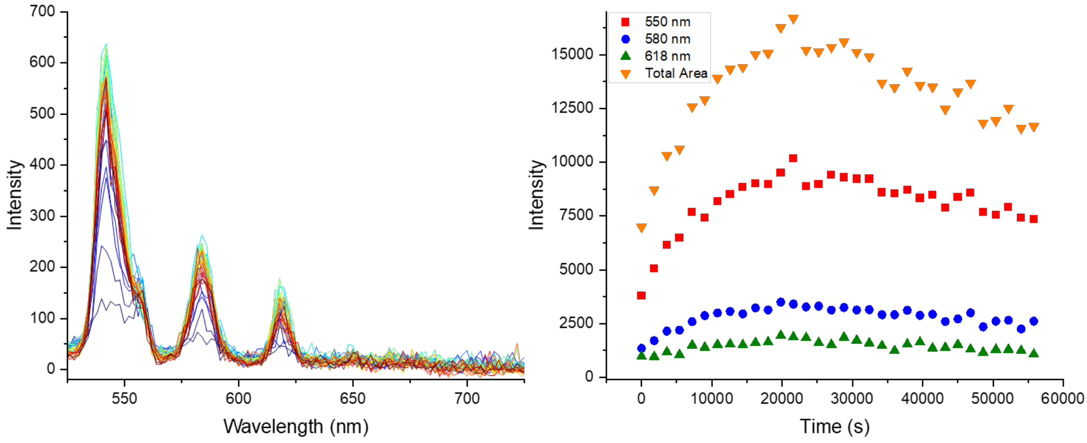

*Figure S45: **2·Tb₂** (0.0001 M) in MES (0.1 M) with zinc and ammonium formate*

3·Tb₂

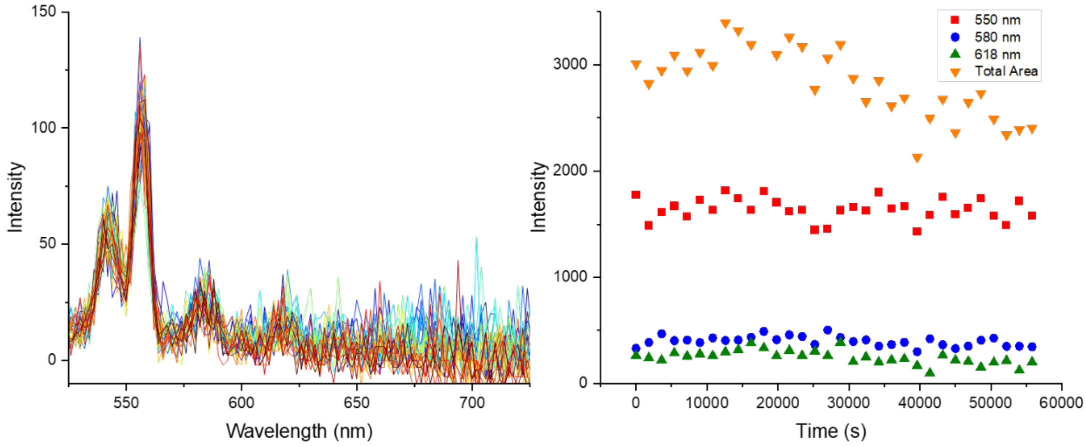

*Figure S46: **3·Tb₂** (0.01 M) in MES (0.1 M)*

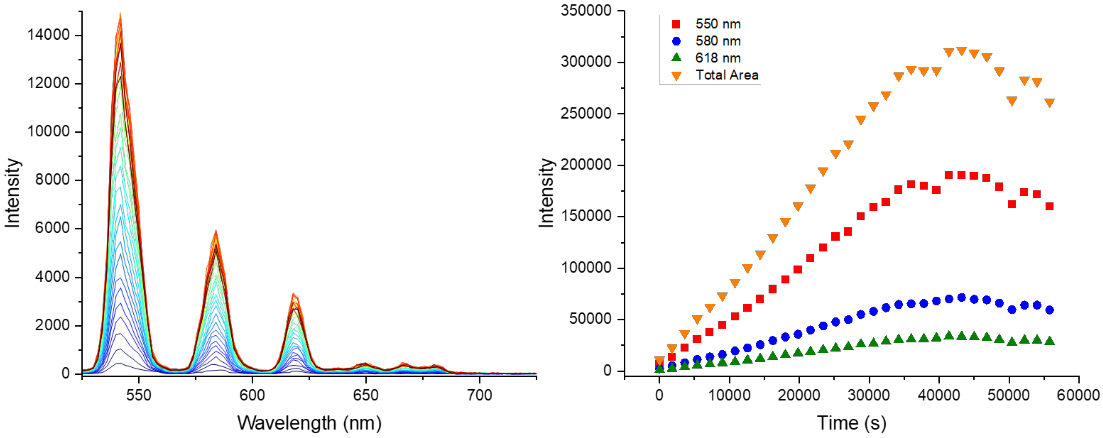

Figure S47: ***3·Tb₂*** *(0.01 M) in MES (0.1 M) with zinc and ammonium formate*

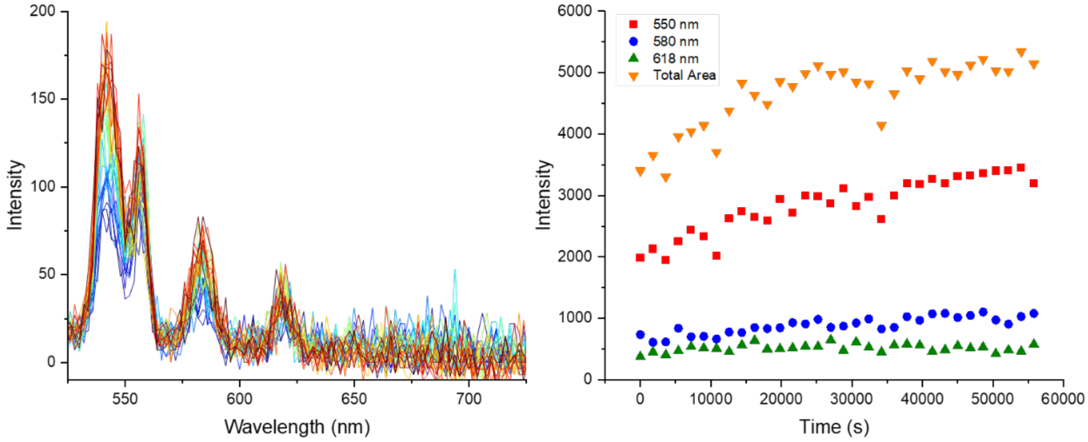

Figure S48: ***3·Tb₂*** *(0.001 M) in MES (0.1 M)*

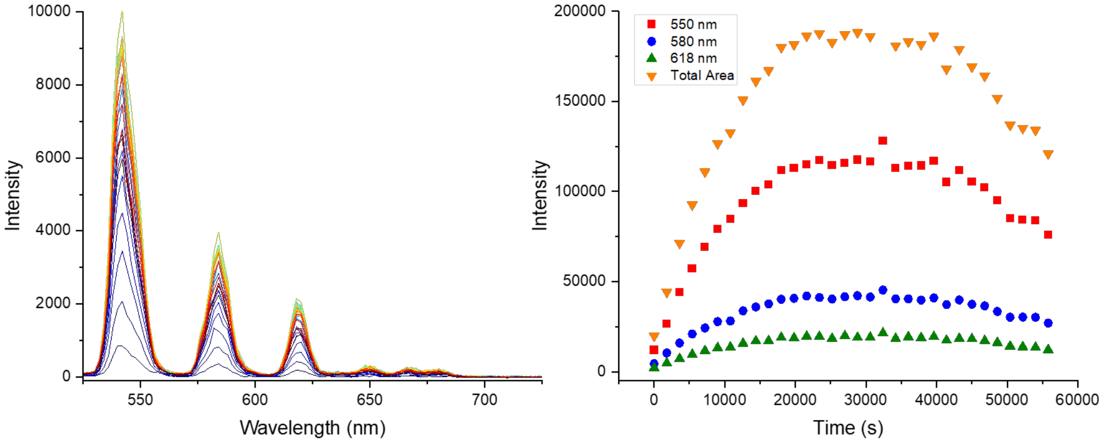

Figure S49: ***3·Tb₂*** *(0.001 M) in MES (0.1 M) with zinc and ammonium formate*

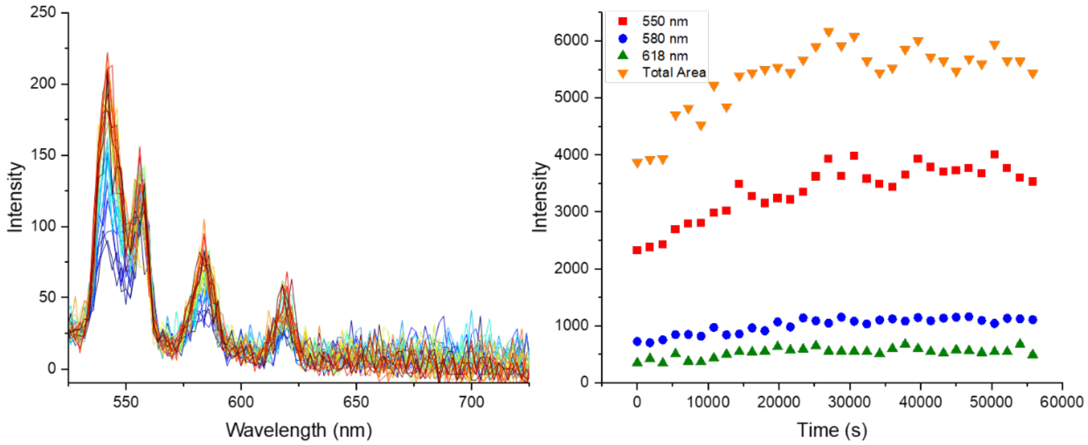

*Figure S50: **3·Tb₂** (0.0001 M) in MES (0.1 M)*

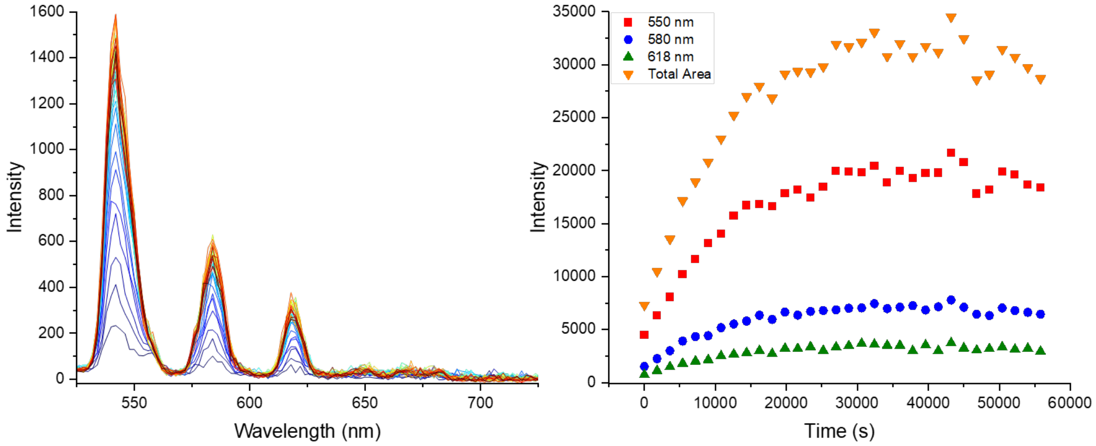

*Figure S51: **3·Tb₂** (0.0001 M) in MES (0.1 M) with zinc and ammonium formate*

Biological reduction of the binuclear Ln(III) probes

NaHS Reduction

A Horiba Jobin Yvon Fluorolog® 3-12 Fluorometer equipped with a Hamamatsu R928 detector and a double-grating emission monochromator was used to investigate the biological reduction of probes **1-4·Tb$_2$** with NaHS. The emission of **1-4·Tb$_2$** (90 µM, PBS pH 7.4) were recorded upon excitation with 318 nm light, emission and excitation slits were fixed at 1 nm each, the step size was 1 nm and the integration time set to 0.1 s. A pale yellow (455 nm) long pass filter was used. Excitation spectra were recorded by monitoring the emission wavelength at 545 nm where emission and excitation slits were fixed at 5 nm and 1 nm respectively, the step size was 1 nm and the integration time set to 0.1 s. To each sample 250 µM of NaHS was added and the spectra recorded again after 5 minutes.

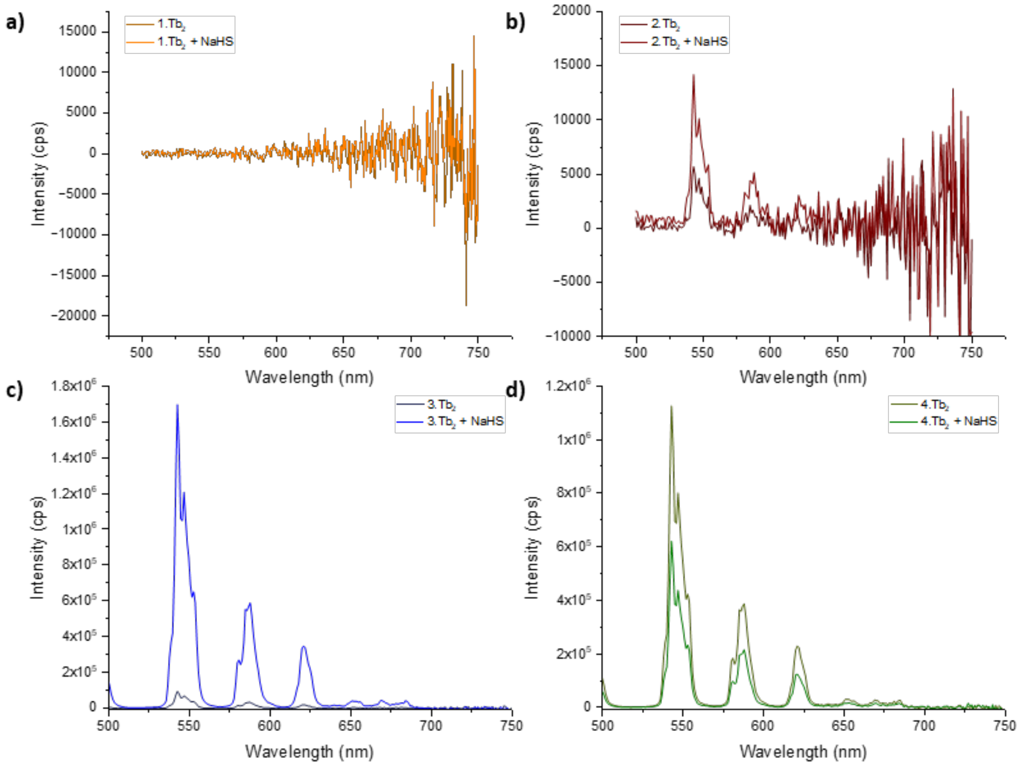

*Figure S52: Emission spectra of complexes **1-4·Tb$_2$** measured at 90 µM, in PBS at pH 7.4 (λ_{exc} = 318 nm, emission slit:1 nm, exit slit: 1 nm) before and after the addition of NaHS (250 µM).*

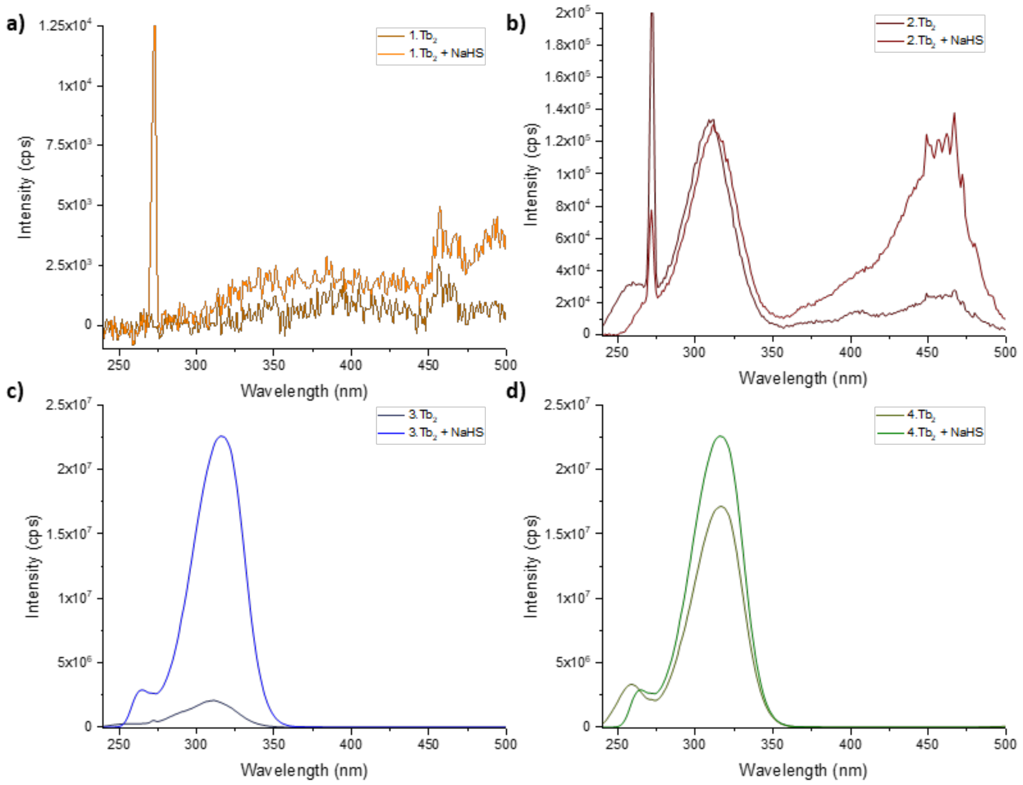

Figure S53: Excitation spectra of complexes ***1-4·Tb$_2$*** *measured at 90 µM, in PBS at pH 7.4 (λ_{em} = 545 nm, emission slit: 5 nm, exit slit: 1 nm) before and after the addition of NaHS (250 µM).*

Cell Studies

1.1 Cell Culture

HCT116 cells were cultured in DMEM medium supplemented with 10% FBS at 37°C, 5% CO_2 in a humidified incubator. All cell lines were routinely mycoplasma tested using a HEK-Blue™ detection kit (Invivogen) and found to be negative.

1.2 Hypoxia exposure

Hypoxia treatments at <0.1% O_2 were carried out in a Bactron II Chamber (Shel Laboratory). Oxygen concentrations were periodically validated using anaerobic oxygen indicator strips (ThermoFisher).

1.4 Fluorescent Microscopy

Cells were seeded onto autoclaved cover slips (Menzel-Glaser) before treatment and hypoxia exposure. Cells were fixed inside the hypoxia chamber in 4% PFA for 10 minutes. Cells were mounted onto microscopy slides (Menzel Glaser) with ProLong™ Gold Antifade Mountant with DAPI to visualise the nucleus (Invitrogen™). Cells were visualised with an LSM780 confocal microscope (Carl Zeiss Microscopy Ltd) at 63x magnification. Excitation and emission wavelengths used were: DAPI 370/470 nm, 488/545 nm.

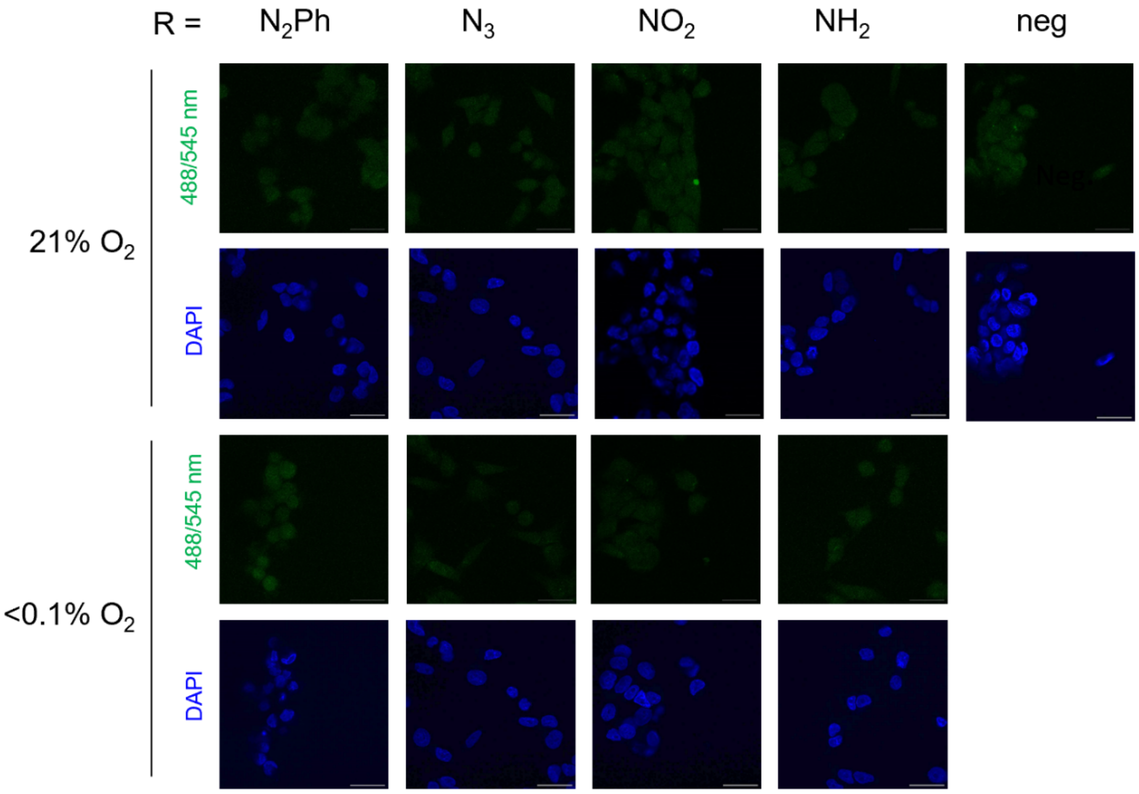

*Figure S54: Fluorescence microscopy of complexes. Representative fluorescence images of HCT116 cells treated with **1·Tb₂**(R= NO_2), **2·Tb₂**(R= N_2Ph), **3·Tb₂** (R= N_3), or **4·Tb₂** (R= NH_2) (500 μM) for 16 h in the oxygen conditions indicated (exc. 488 nm / em. 545 nm). Scale bar represents 12 μM; blue colour represents DAPI stain.*

Biocatalytic Reduction

Catalyst preparation was carried out in a glove box (Glove Box Technology Ltd.) under a protective N_2 atmosphere (O_2 < 3 ppm). A 20 mg/mL carbon black suspension in PB (50 mM, pH 6.0) was sonicated for 1 hour. For the preparation of the catalyst for four 0.5 mL scale reaction with 10 mM concentration of substrate, 79.2 µL of this suspension was transferred to an Eppendorf tube, 23.1 µL of Hyd-1 solution (1.71 mg/mL) was added (C:Hyd-1 = 40:1 mass ratio), the mixture was gently mixed and left in the fridge (4 °C) for 1 hour. After that, the suspension of the catalyst was centrifuged (3 min, 14500 rpm), the supernatant was removed by pipetting, and the catalyst was resuspended in 75 µL of PB (50 mM, pH 6.0 unless stated otherwise). Resuspension-centrifugation-pipetting steps were repeated 3 times, and then the catalyst was resuspended in 75 µL of PB and directly used for the reaction.

Reaction set-up was carried out in a glove box (Glove Box Technology Ltd.) under a protective N_2 atmosphere (O_2 < 3 ppm). Reactions were run on a 0.5 mL scale with a 10 mM concentration of substrate in PB (50 mM, pH 6.0) or with 10% v/v of DMSO at room temperature under a gentle H_2 flow in an Asynt Octo Mini reactor. A stock solution of substrate in buffer or DMSO was transferred to a reaction vessel, 25 µL of catalyst was added, and the volume was adjusted with the corresponding buffer to a total volume of 0.5 mL. The reactor was closed and removed from the glove box. The H_2 line was connected, and reactions were run at a 30-40 mL/min flow of H_2.

After 24 h of reaction time, the reaction mixture was centrifuged (3 min, 14500 rpm) and the supernatant was transferred, diluted to 90 µM concentration and the fluorescence spectrum was recorded for each sample. Reaction with **2·Tb$_2$** (N=N substrate) was complete after 24 hours. Reactions with **1·Tb$_2$** (NO_2 substrate) and **3·Tb$_2$** (N_3 substrate) were subjected to reaction conditions with freshly prepared catalyst (25 µL of catalyst for each reaction). After 72 h of reaction time **3·Tb$_2$** was fully converted to the corresponding amine, which was confirmed by luminescence emission and excitation spectroscopy.

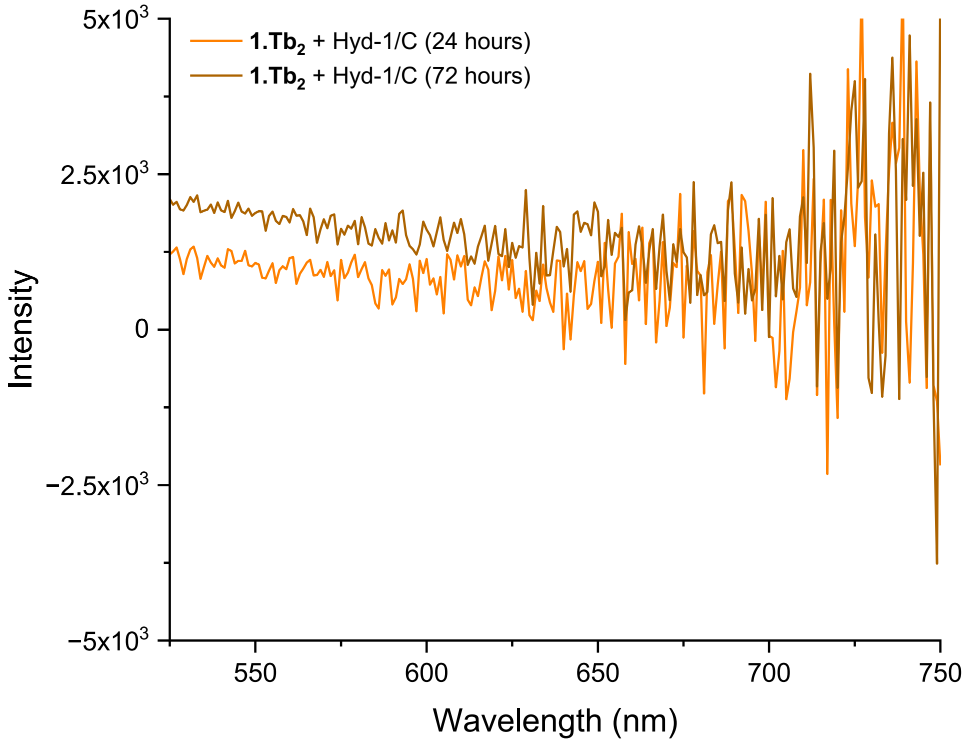

*Figure S55: Emission spectra of **1·Tb$_2$** upon exposure to Hyd-1/C under H_2 Fat pH 6.0 λ_{ex} = 318 nm, excitation slit 3 nm, emission slit 1 nm, integration time 0.5 s, with Thorlabs 400 nm bandpass filter.*

^{1}H and ^{13}C spectra of compounds

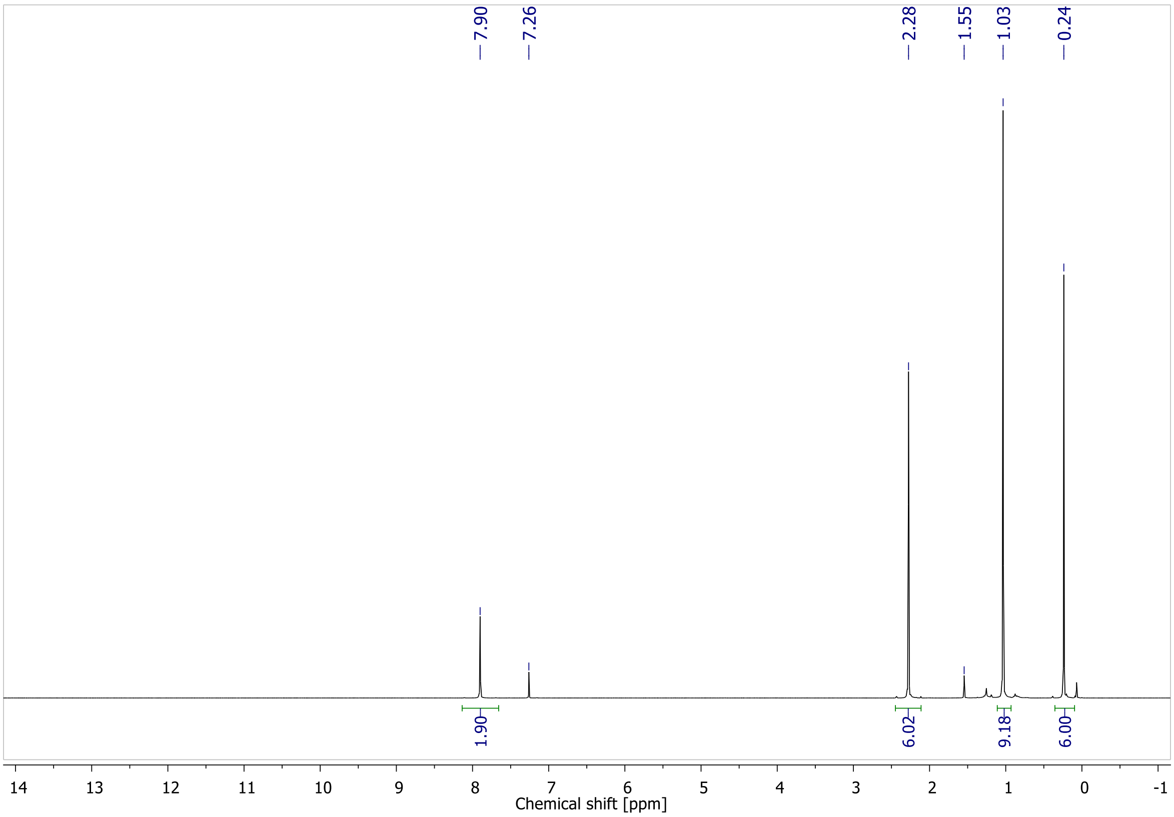

*Figure S54: ^{1}H NMR spectrum of **1b** (400 MHz, $CDCl_3$).*

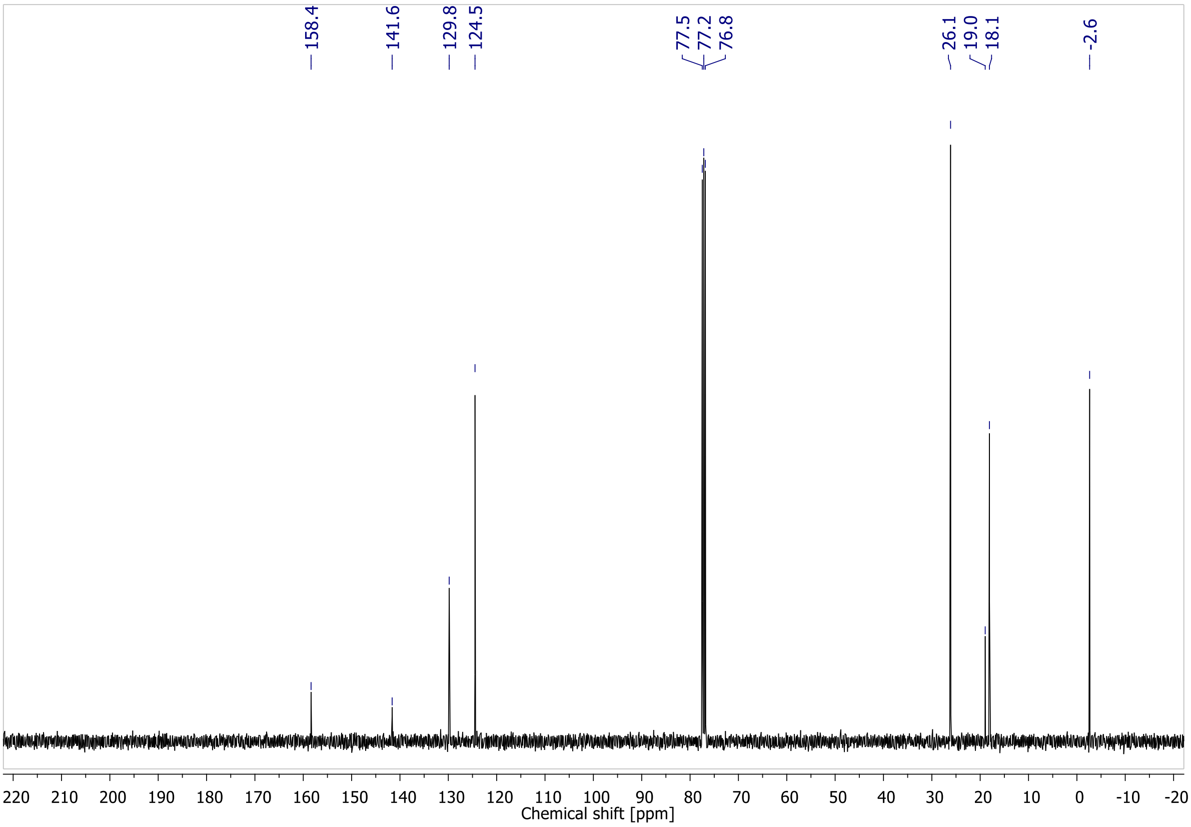

*Figure S55: ^{13}C NMR spectrum of **1b** (400 MHz, $CDCl_3$).*

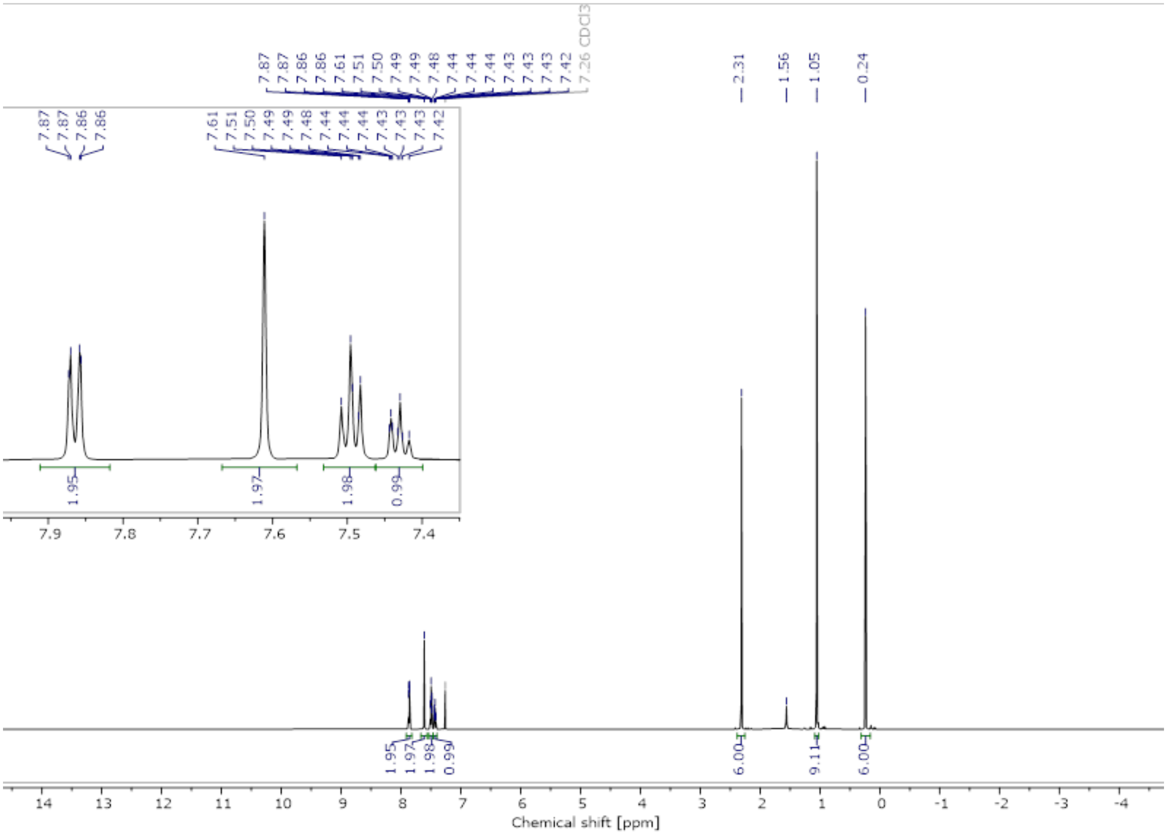

Figure S56: ^{1}H NMR spectrum of **2b** (600 MHz, $CDCl_3$).

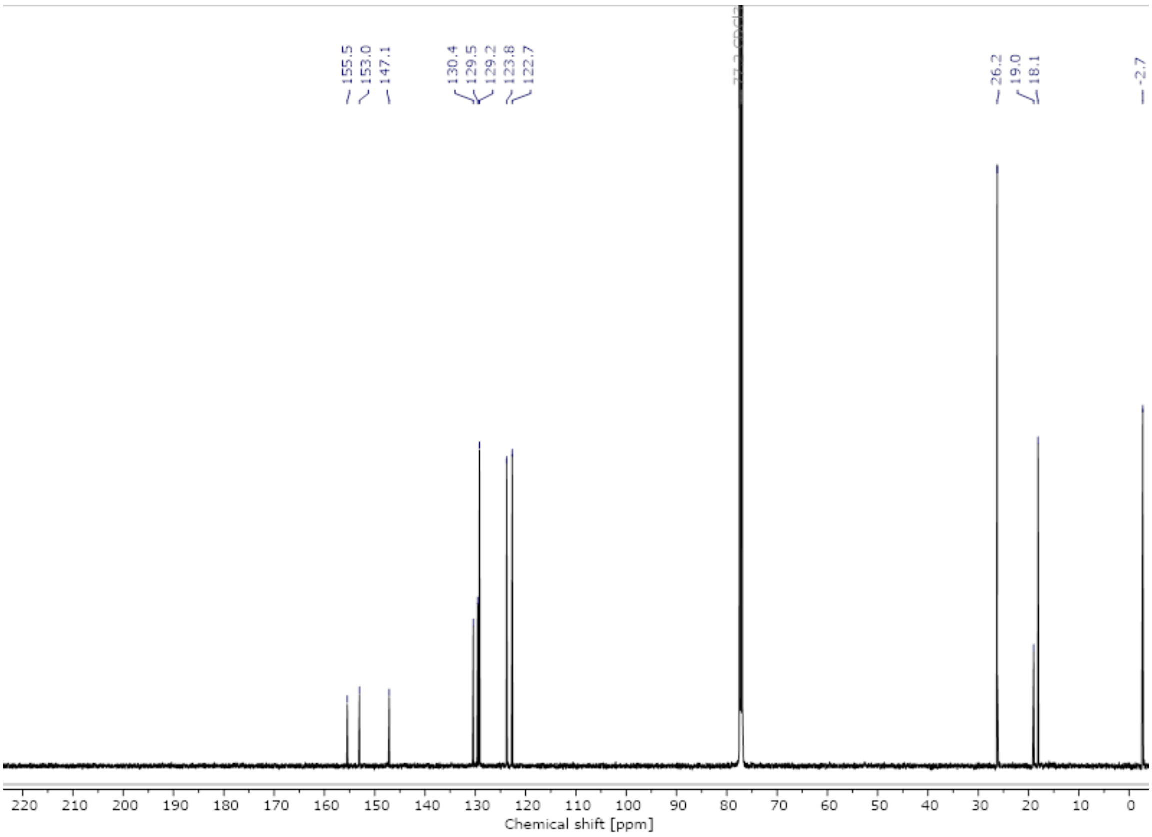

Figure S57: ^{13}C NMR spectrum of **2b** (151 MHz, $CDCl_3$).

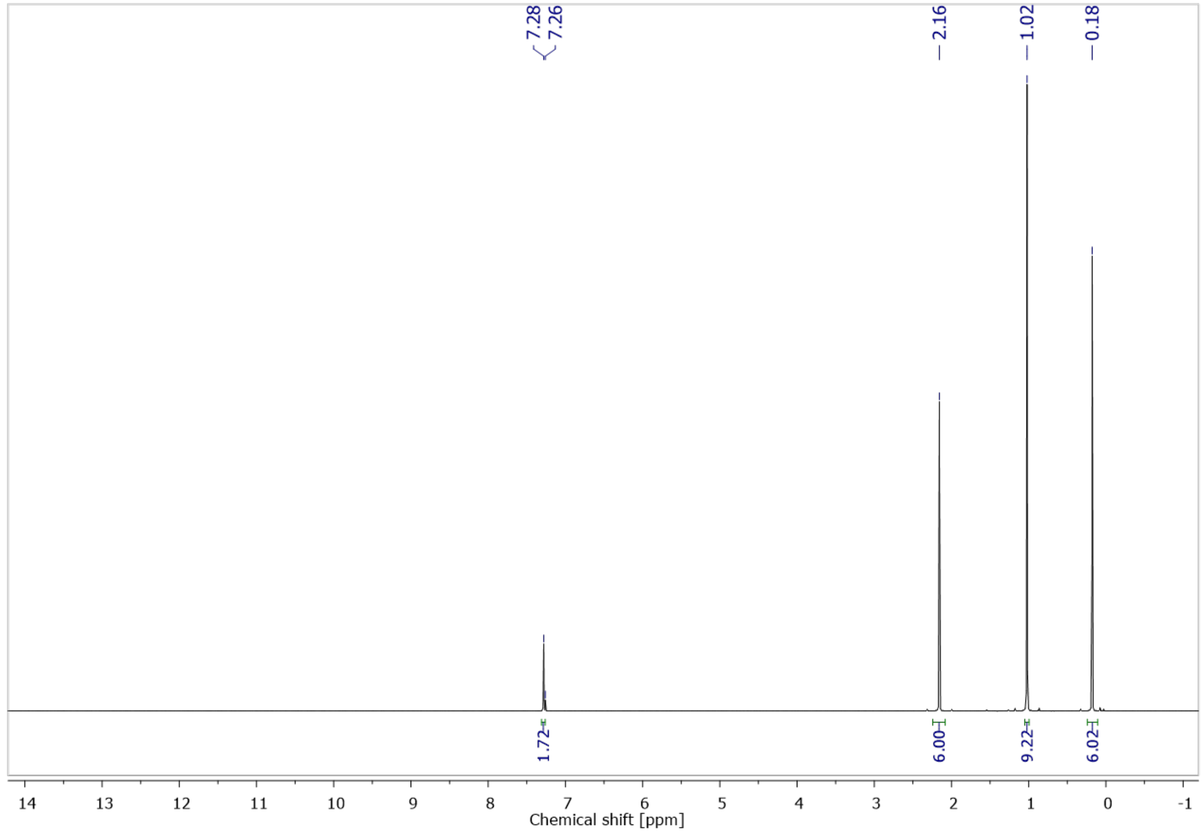

*Figure S58: ¹H NMR spectrum of **tert-butyl(4-iodo-2,6-dimethylphenoxy)dimethylsilane** (400 MHz, $CDCl_3$)*

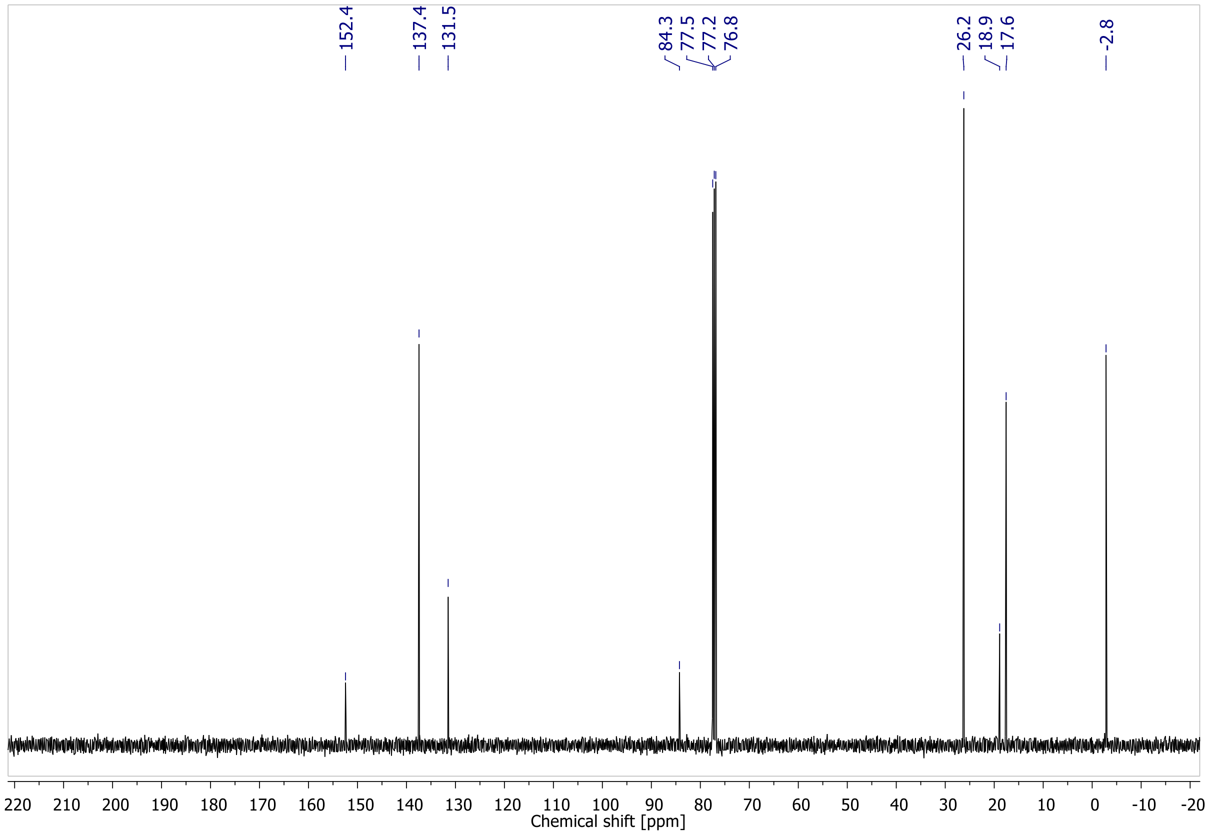

*Figure S59: ¹³C NMR spectrum of **tert-butyl(4-iodo-2,6-dimethylphenoxy)dimethylsilane** (400 MHz, $CDCl_3$)*

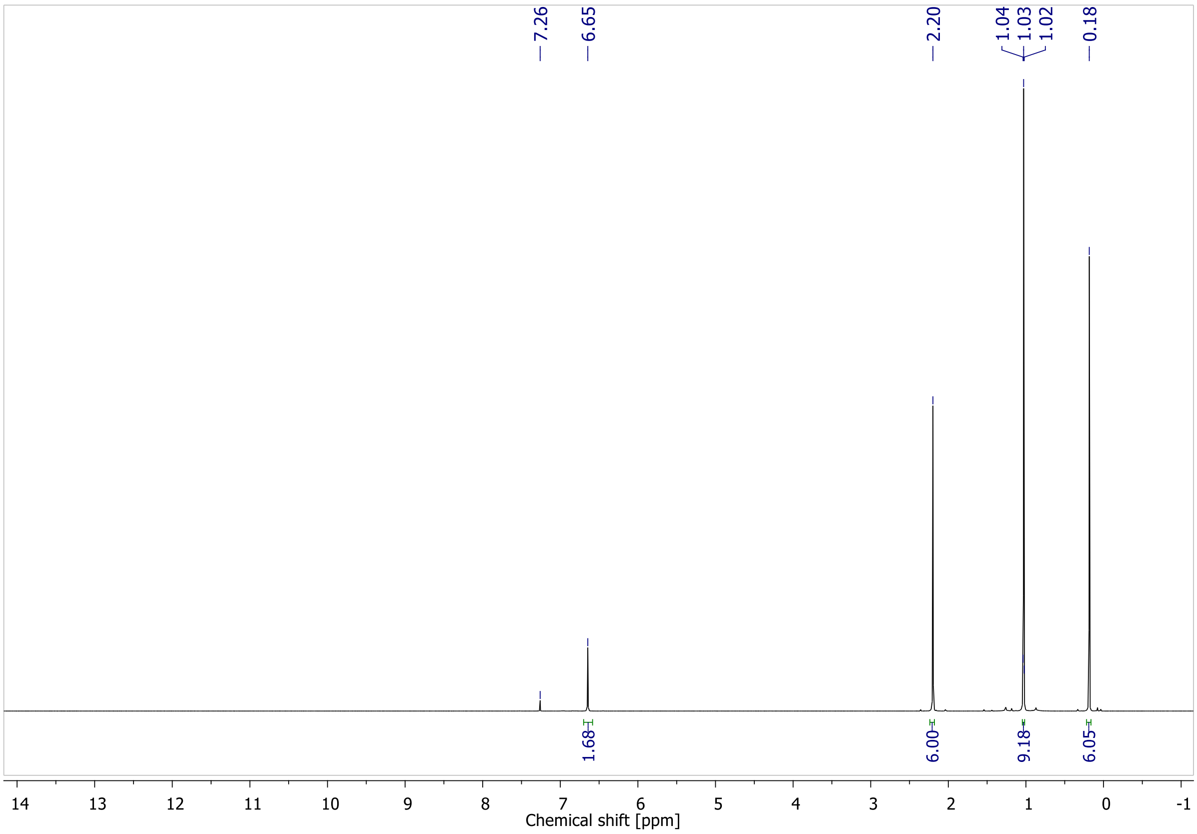

*Figure S60: ^{1}H NMR spectrum of **3b** (400 MHz, $CDCl_3$)*

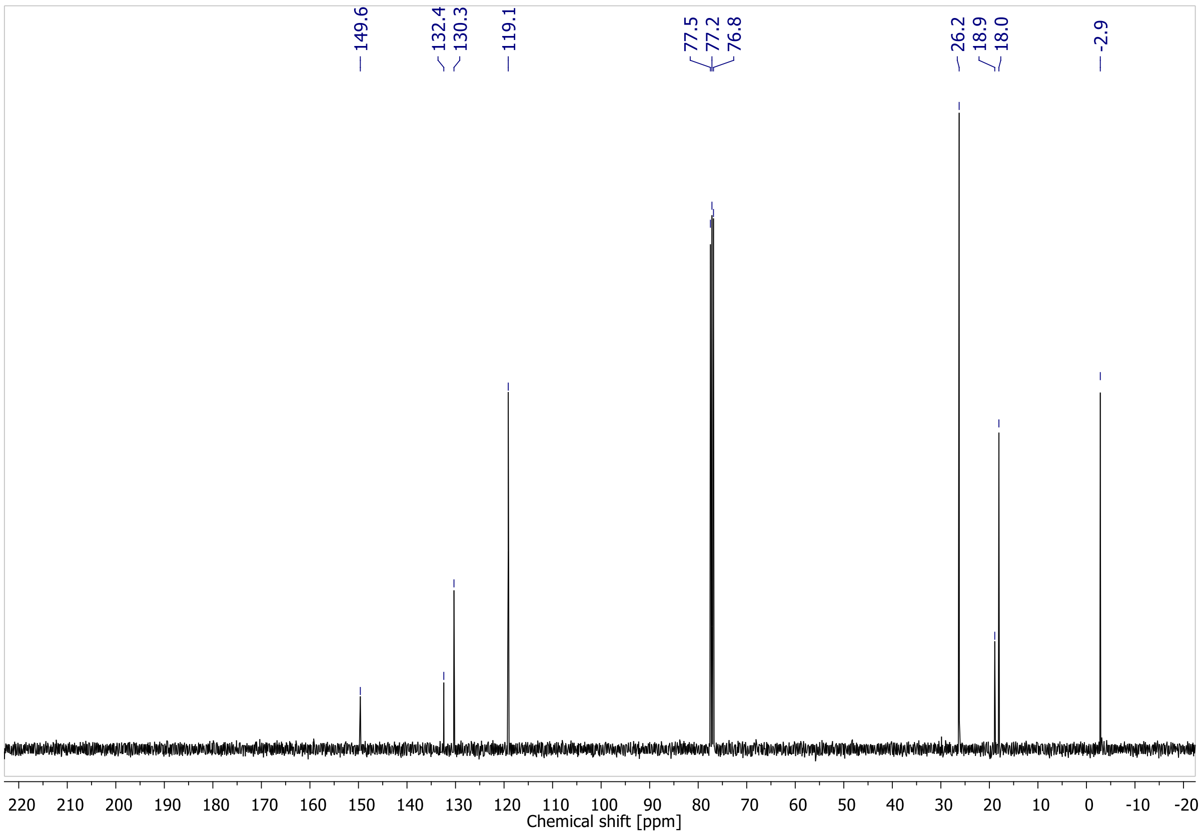

*Figure S61: ^{13}C NMR spectrum of **3b** (400 MHz, $CDCl_3$)*

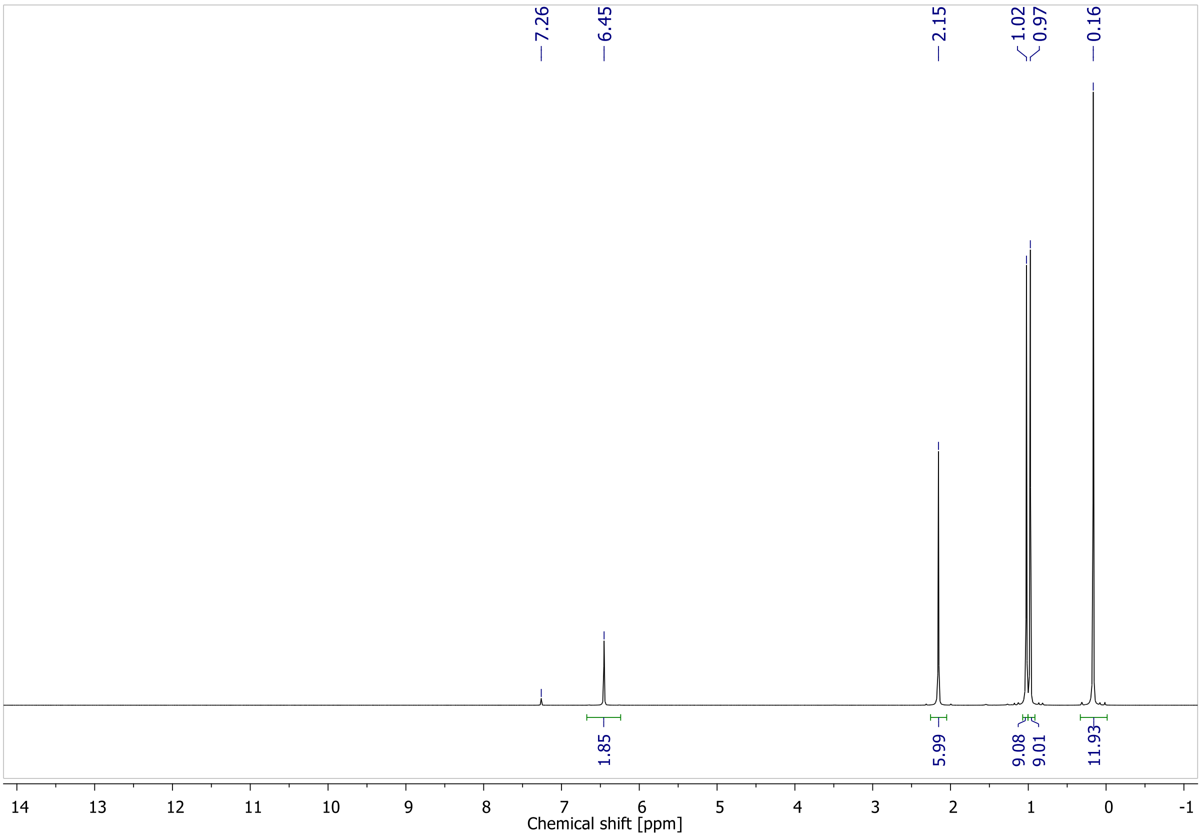

*Figure S62 ^{1}H NMR spectrum of **5b** (400 MHz, CDCl$_3$).*

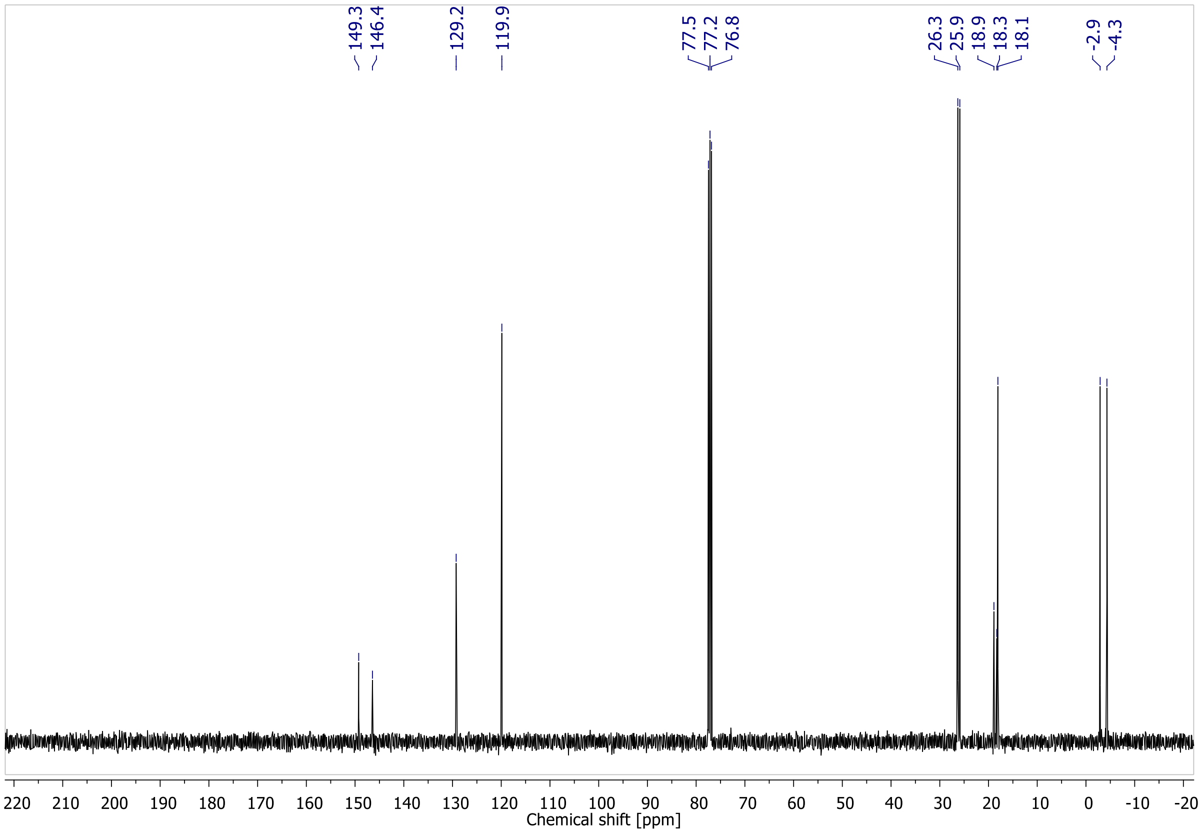

*Figure S63: ^{13}C NMR spectrum of **5b** (400 MHz, CDCl$_3$).*

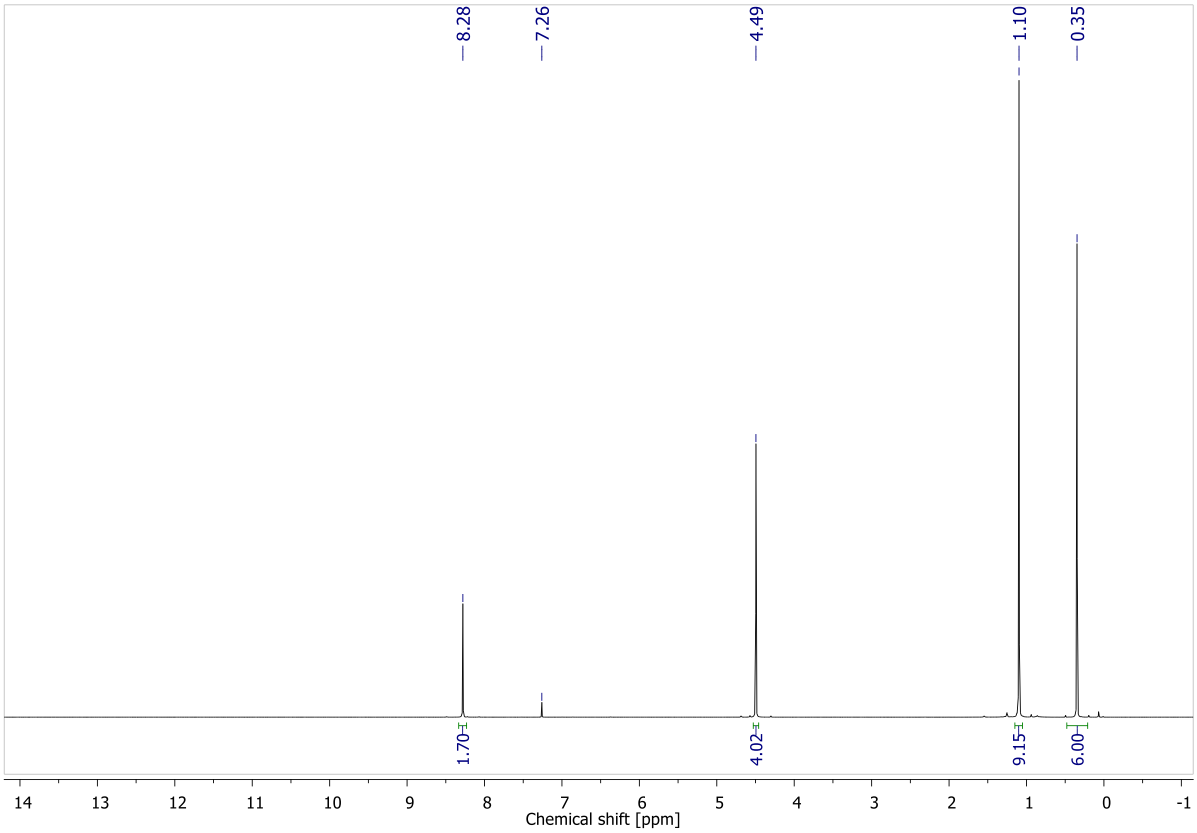

*Figure S64: ^{1}H NMR spectrum of **1c** (400 MHz, $CDCl_3$).*

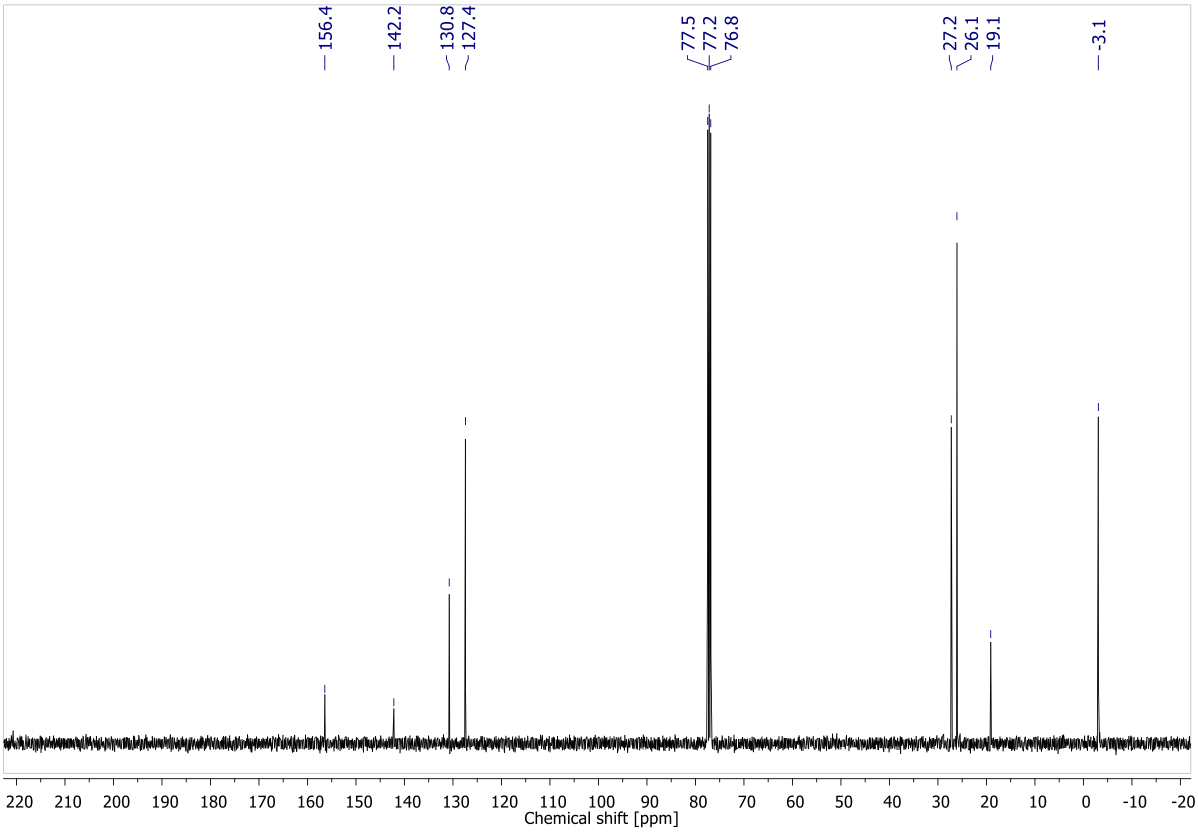

*Figure S65: ^{13}C NMR spectrum of **1c** (400 MHz, $CDCl_3$).*

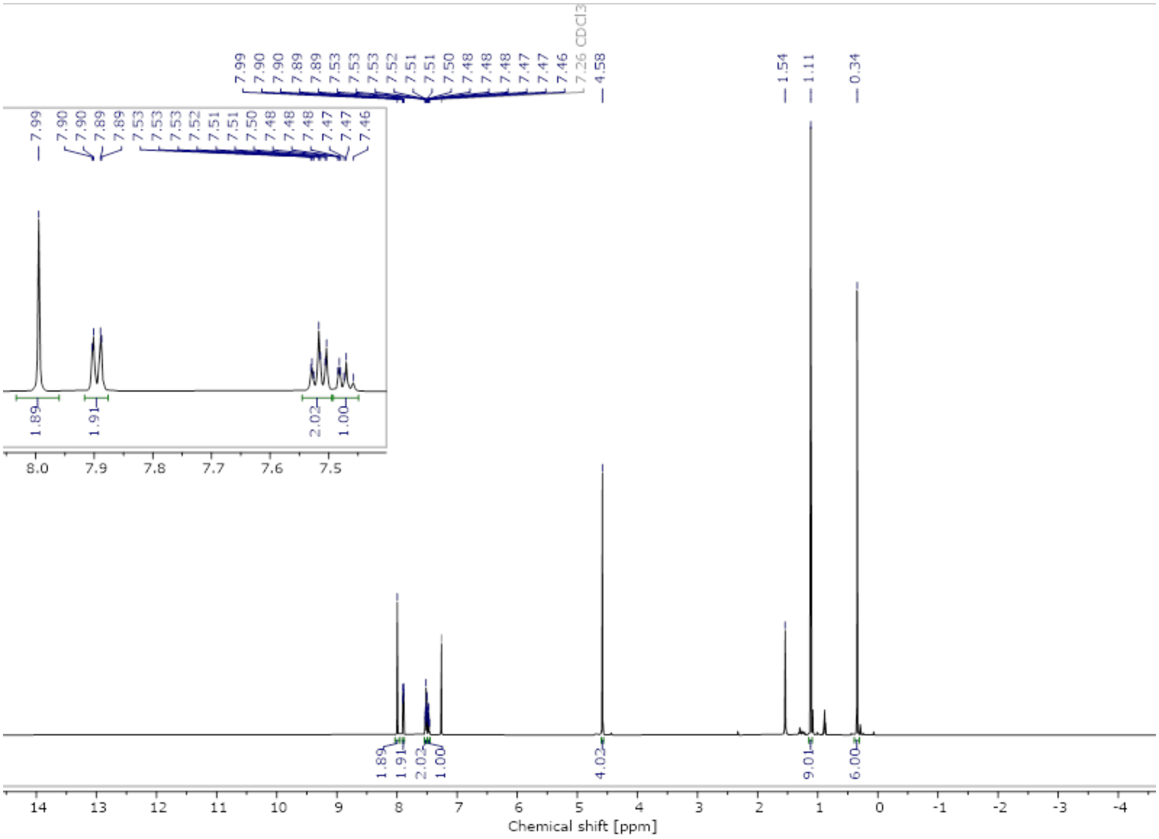

*Figure S66: ^{1}H NMR spectrum of **2c** (600 MHz, $CDCl_3$).*

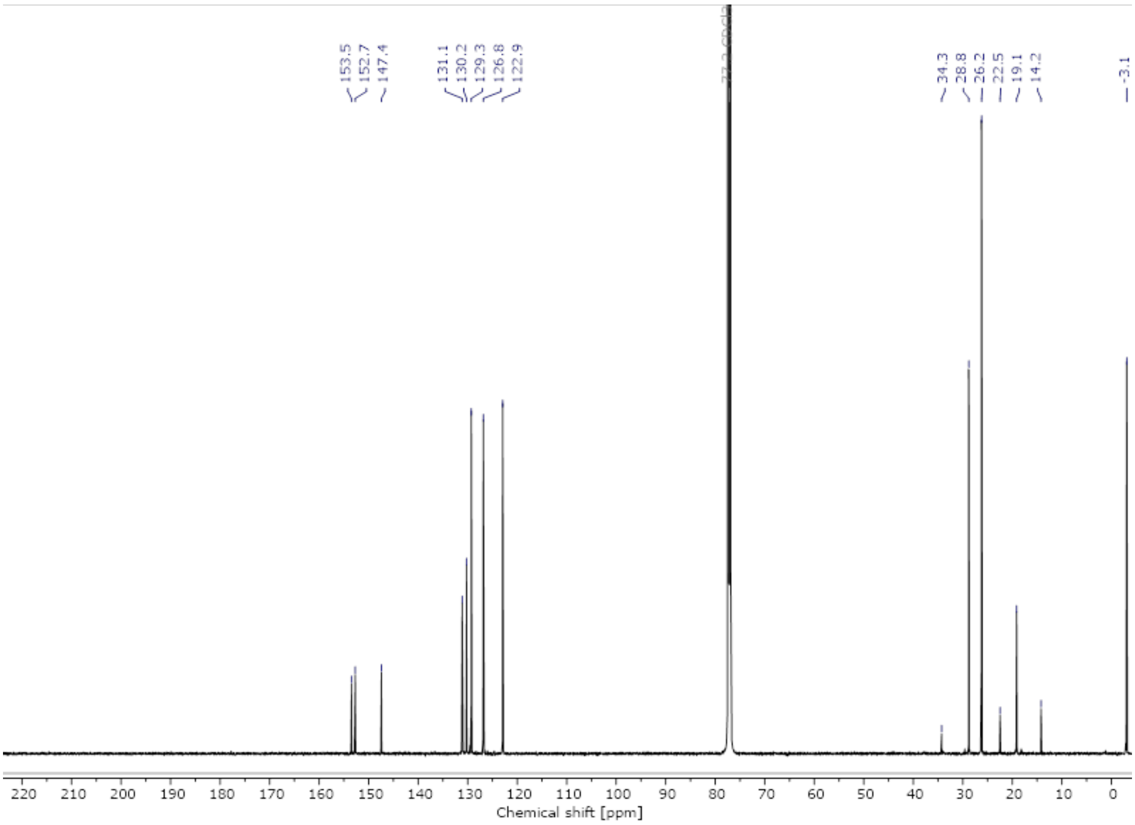

*Figure S67: ^{13}C NMR spectrum of **2c** (151 MHz, $CDCl_3$).*

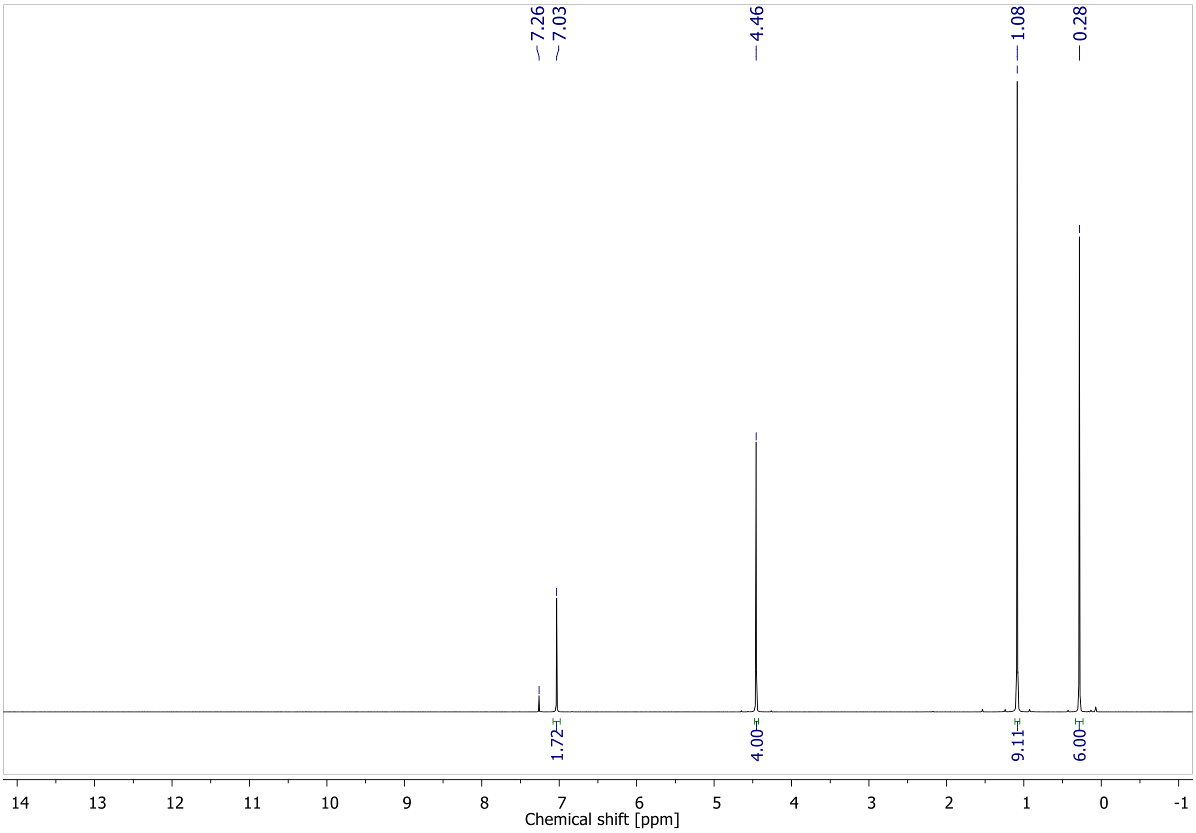

*Figure S68: ^{1}H NMR spectrum of **3c** (400 MHz, $CDCl_3$)*

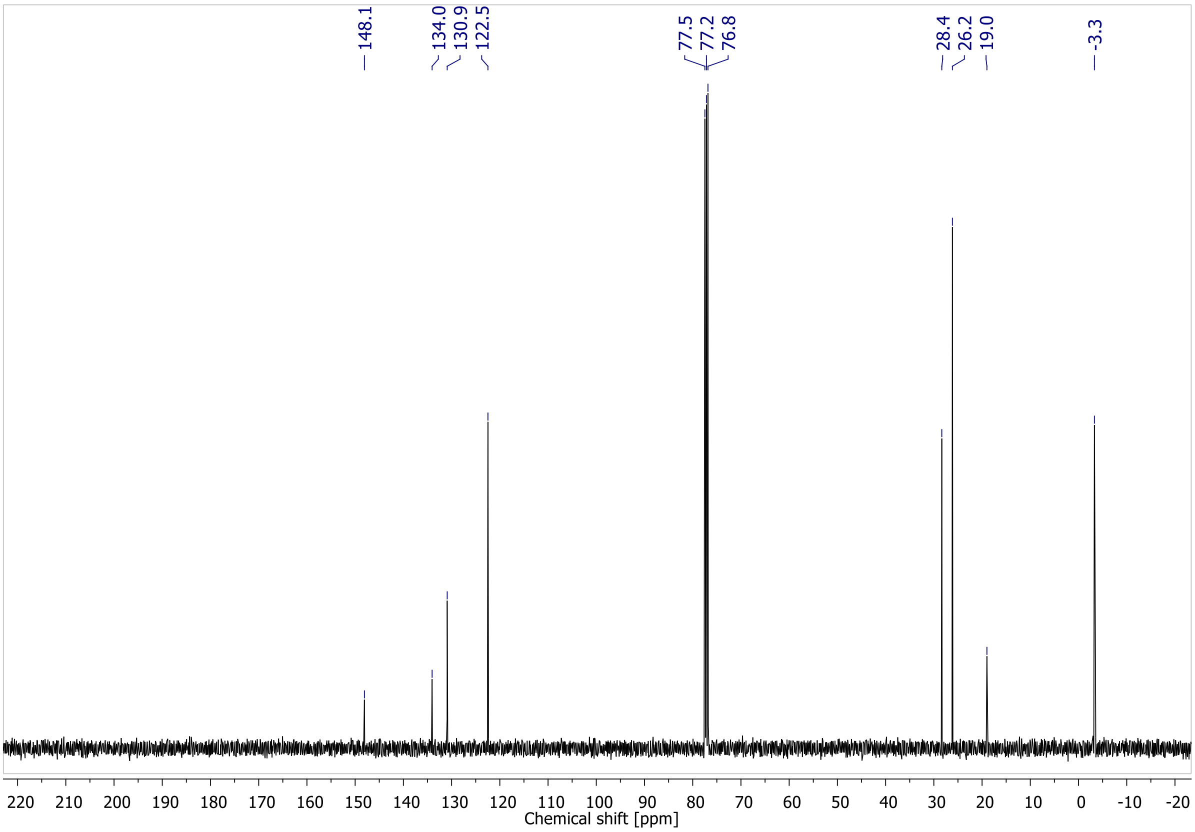

*Figure S69: ^{13}C NMR spectrum of **3c** (400 MHz, $CDCl_3$)*

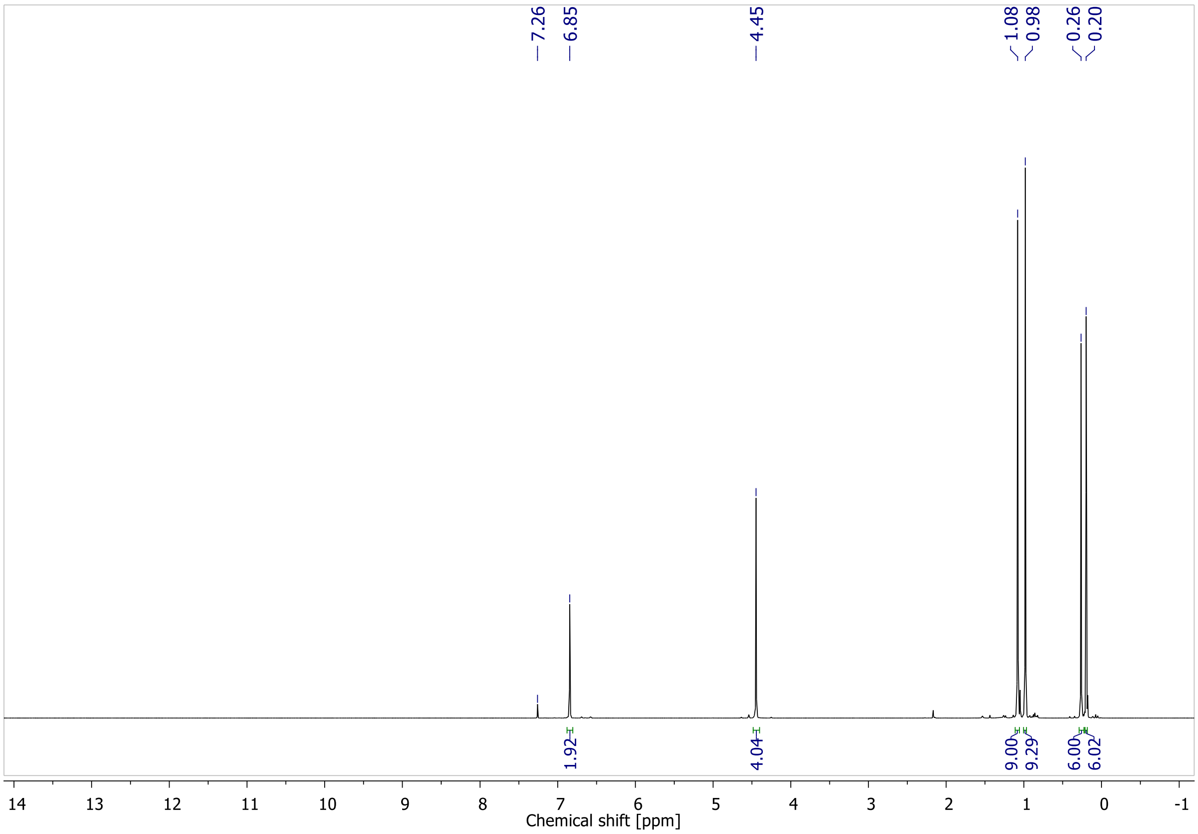

*Figure S70 ^{1}H NMR spectrum of **5c** (400 MHz, $CDCl_3$)*

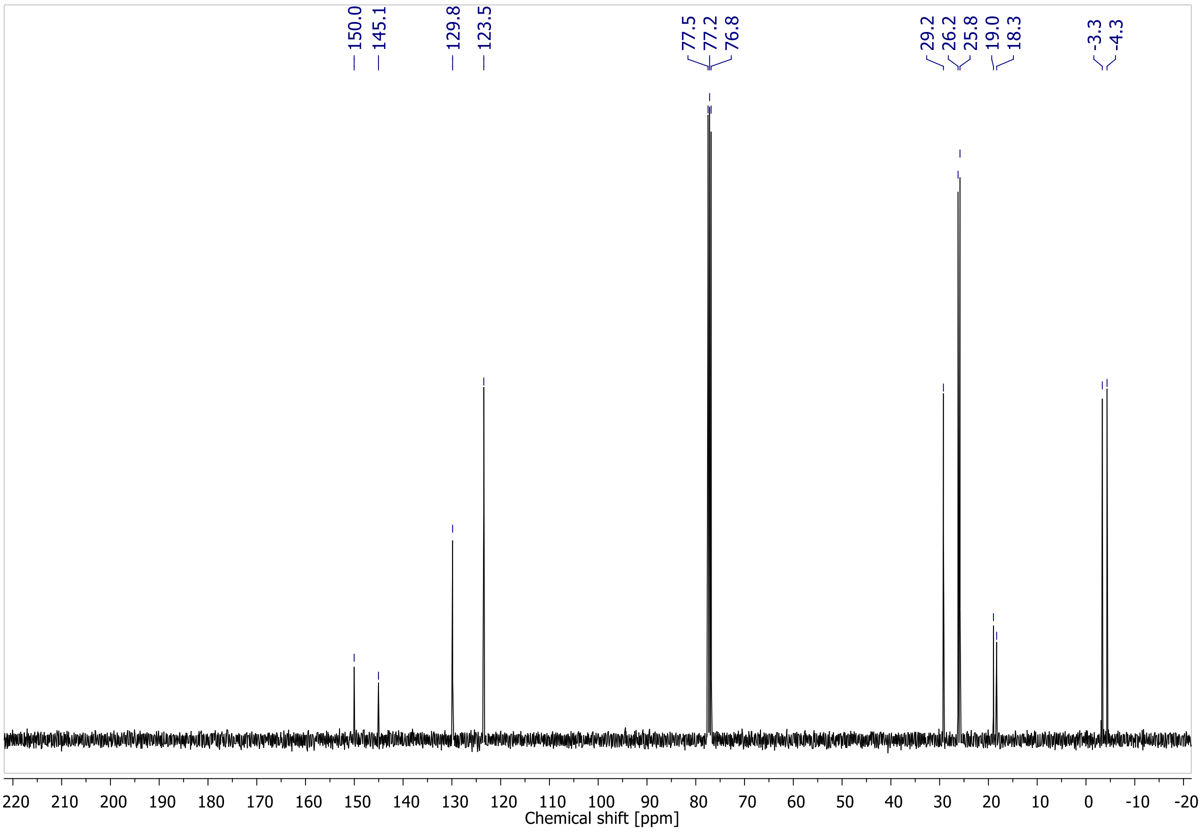

*Figure S71: ^{13}C NMR spectrum of **5c** (400 MHz, $CDCl_3$)*

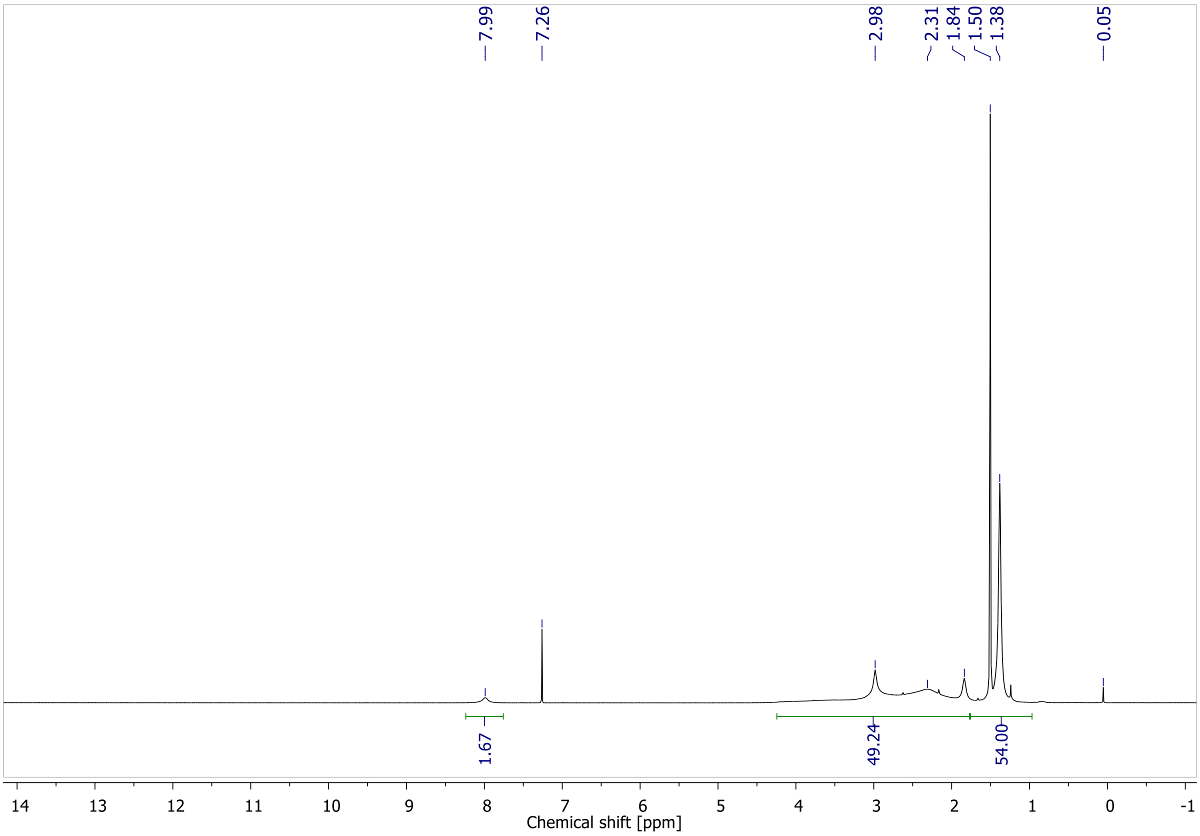

Figure S72: ^{1}H NMR spectrum of ***1e*** *(400 MHz, $CDCl_3$)*

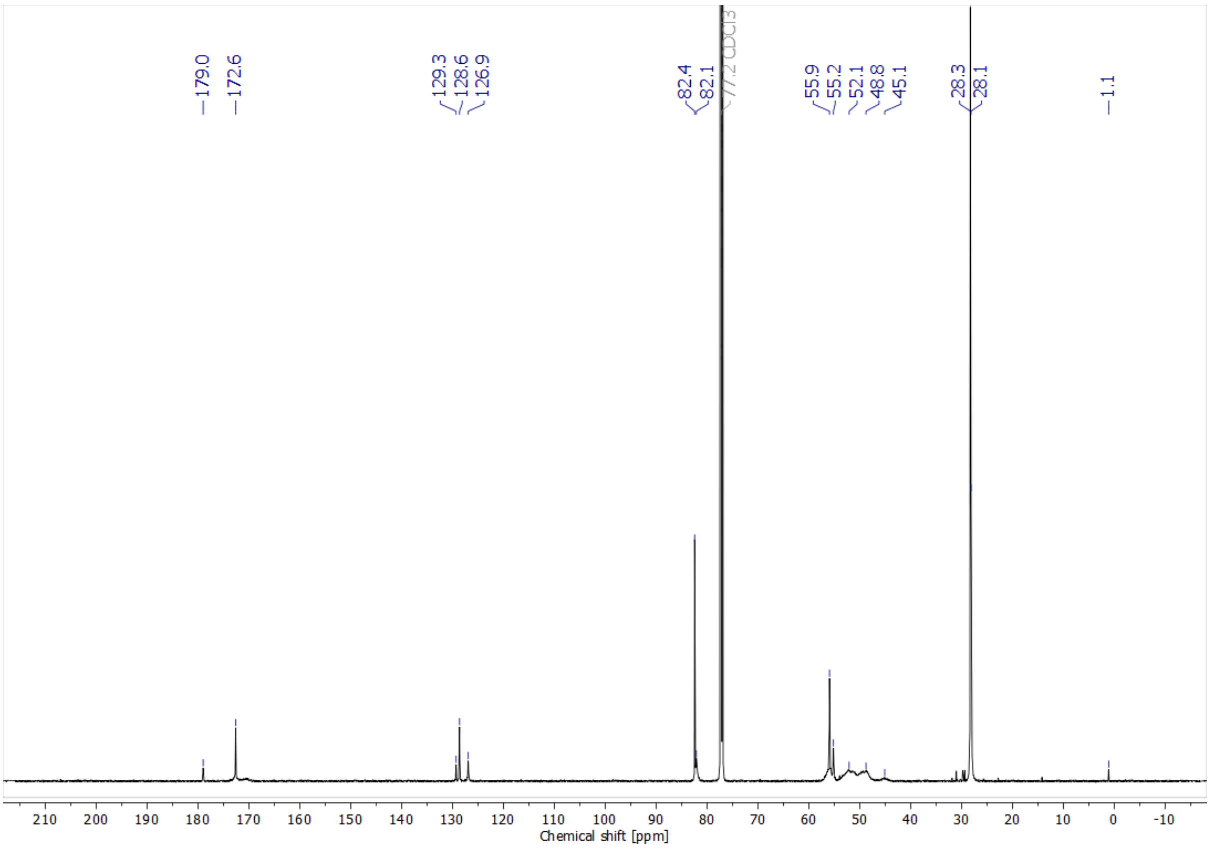

Figure S73: ^{13}C NMR spectrum of ***1e*** *(151 MHz, $CDCl_3$)*

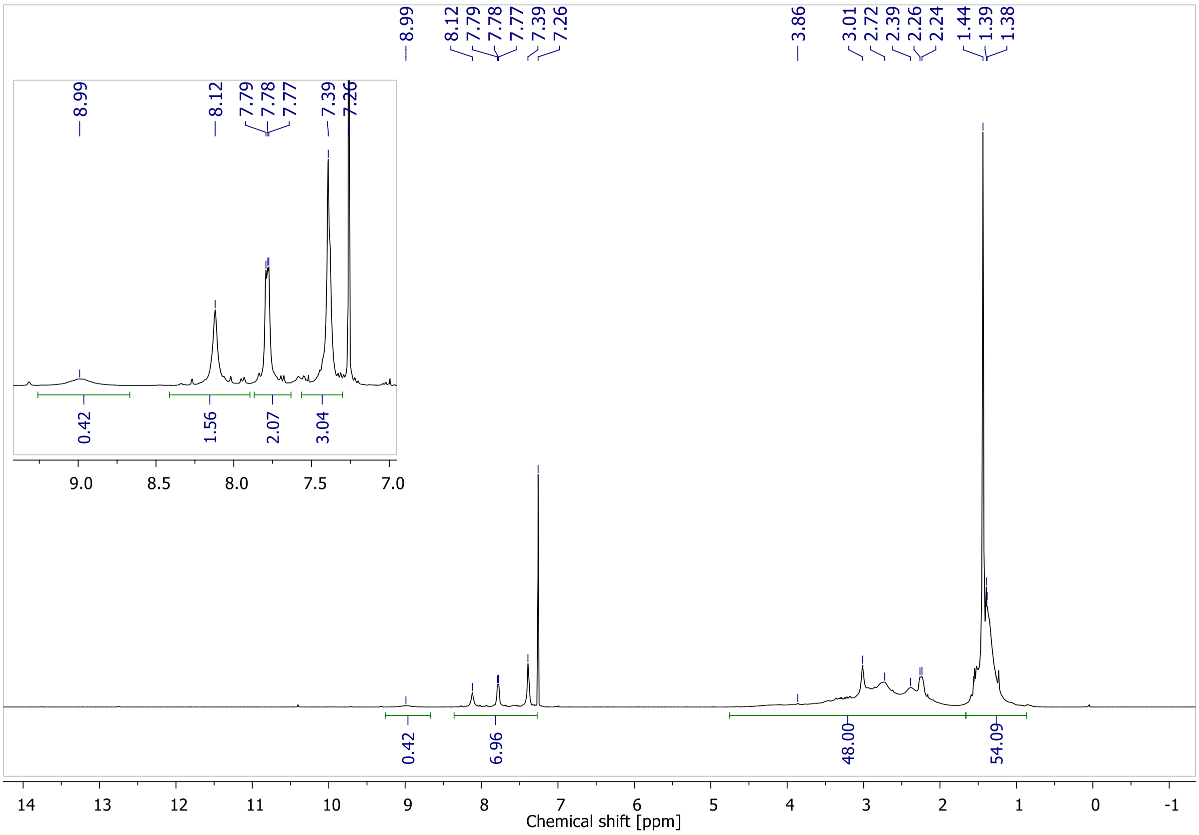

*Figure S74: ^{1}H NMR spectrum of **2e** (400 MHz, $CDCl_3$)*

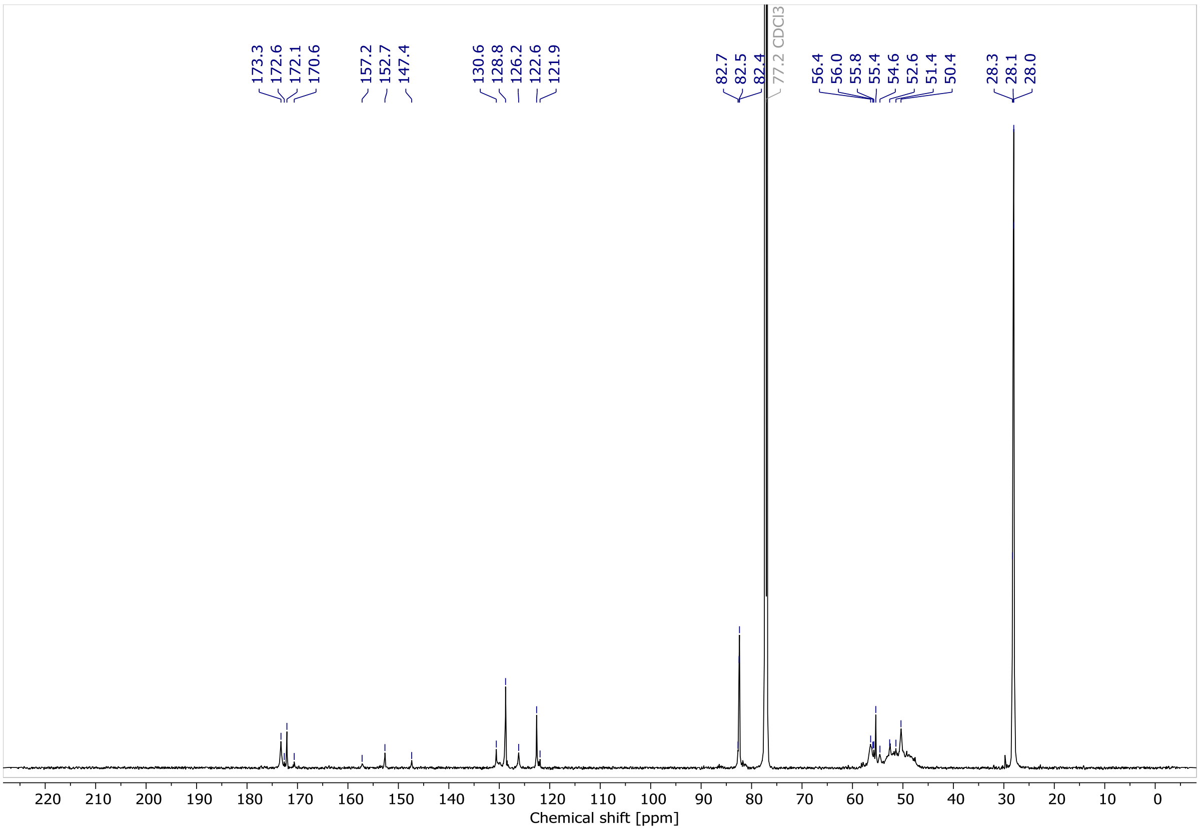

*Figure S75: ^{13}C NMR spectrum of **2e** (151 MHz, $CDCl_3$)*

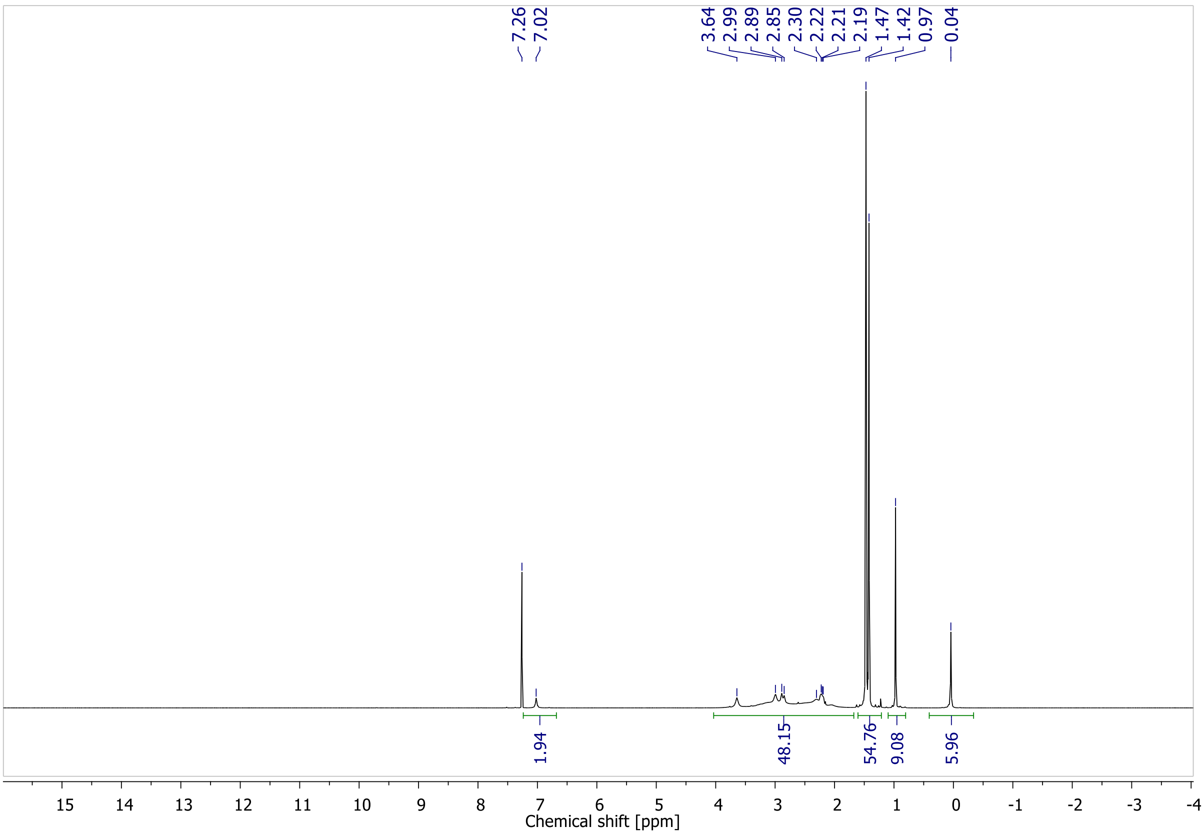

*Figure S76: ^{1}H NMR spectrum of **3d** (400 MHz, $CDCl_3$)*

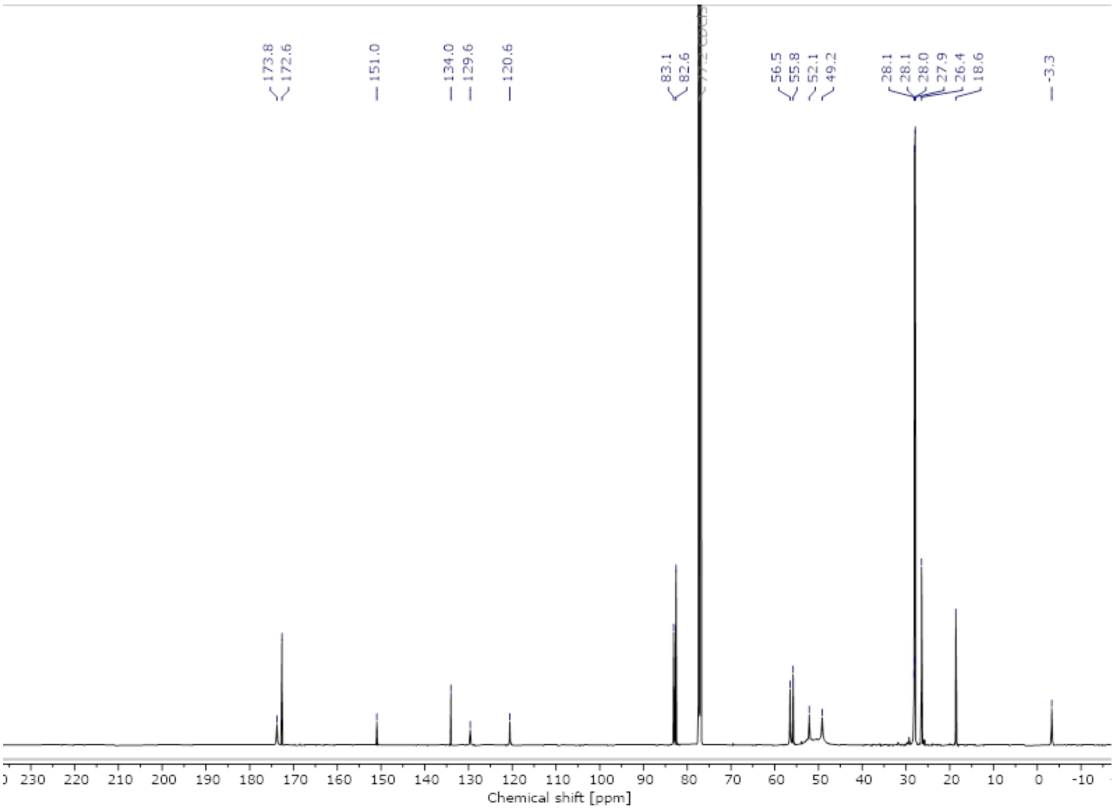

*Figure S77: ^{13}C NMR spectrum of **3d** (151 MHz, $CDCl_3$).*

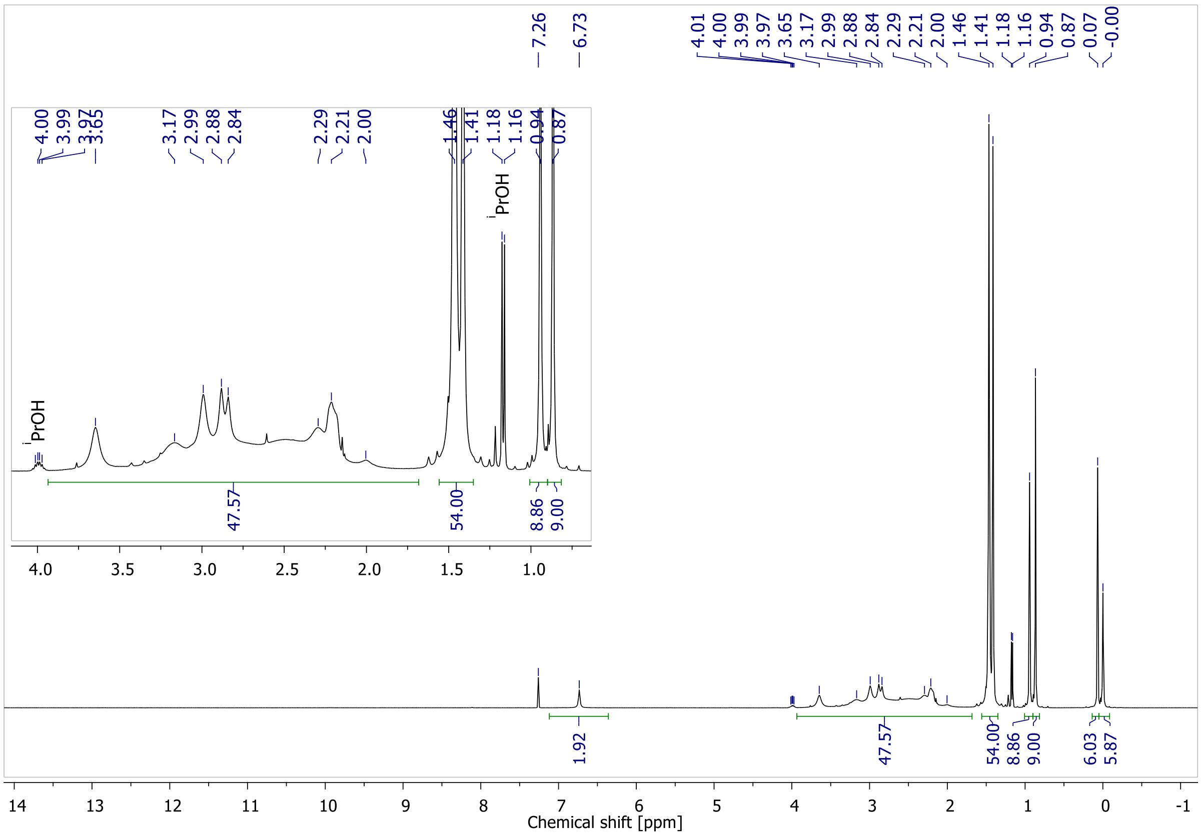

*Figure S78: ^{1}H NMR spectrum of **5d** (400 MHz, $CDCl_3$)*

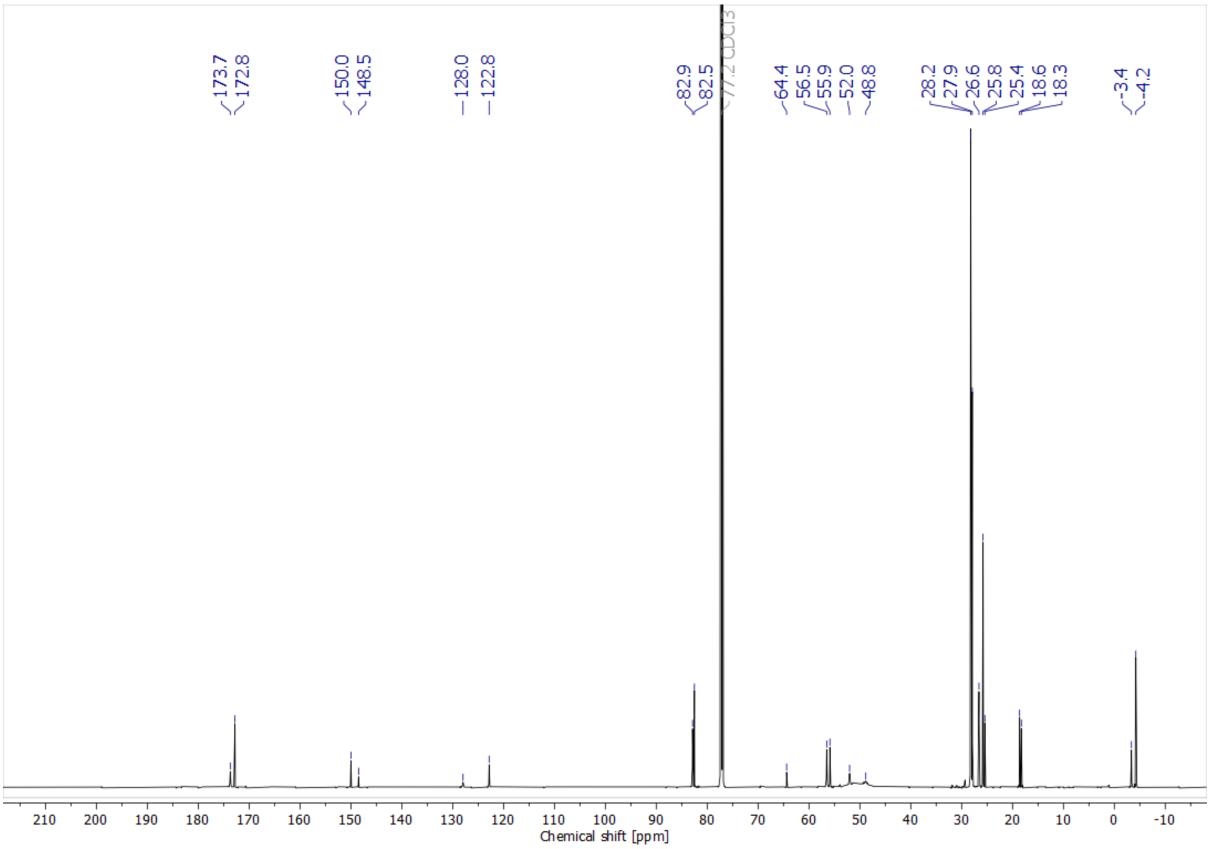

*Figure S79 ^{13}C NMR spectrum of **5d** (151 MHz, $CDCl_3$).*

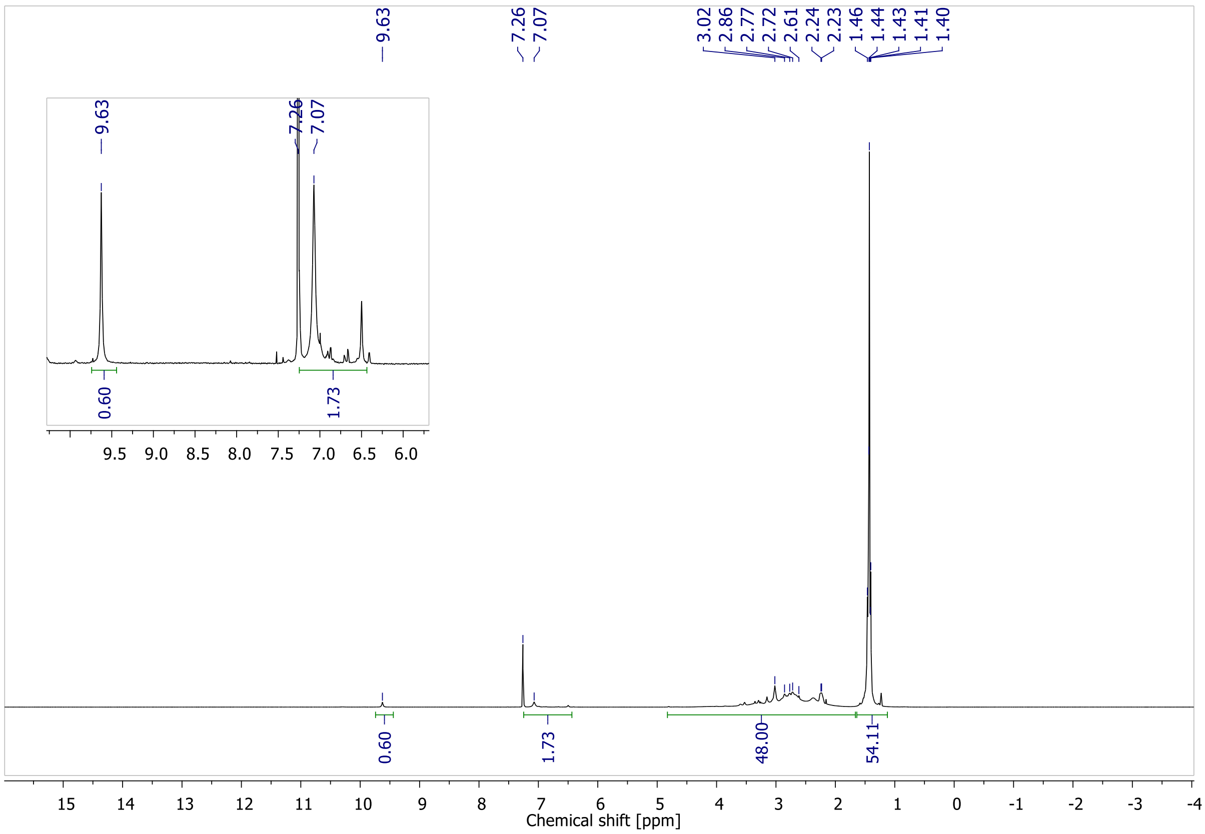

*Figure S80: ^{1}H NMR spectrum of **3e** (400 MHz, $CDCl_3$)*

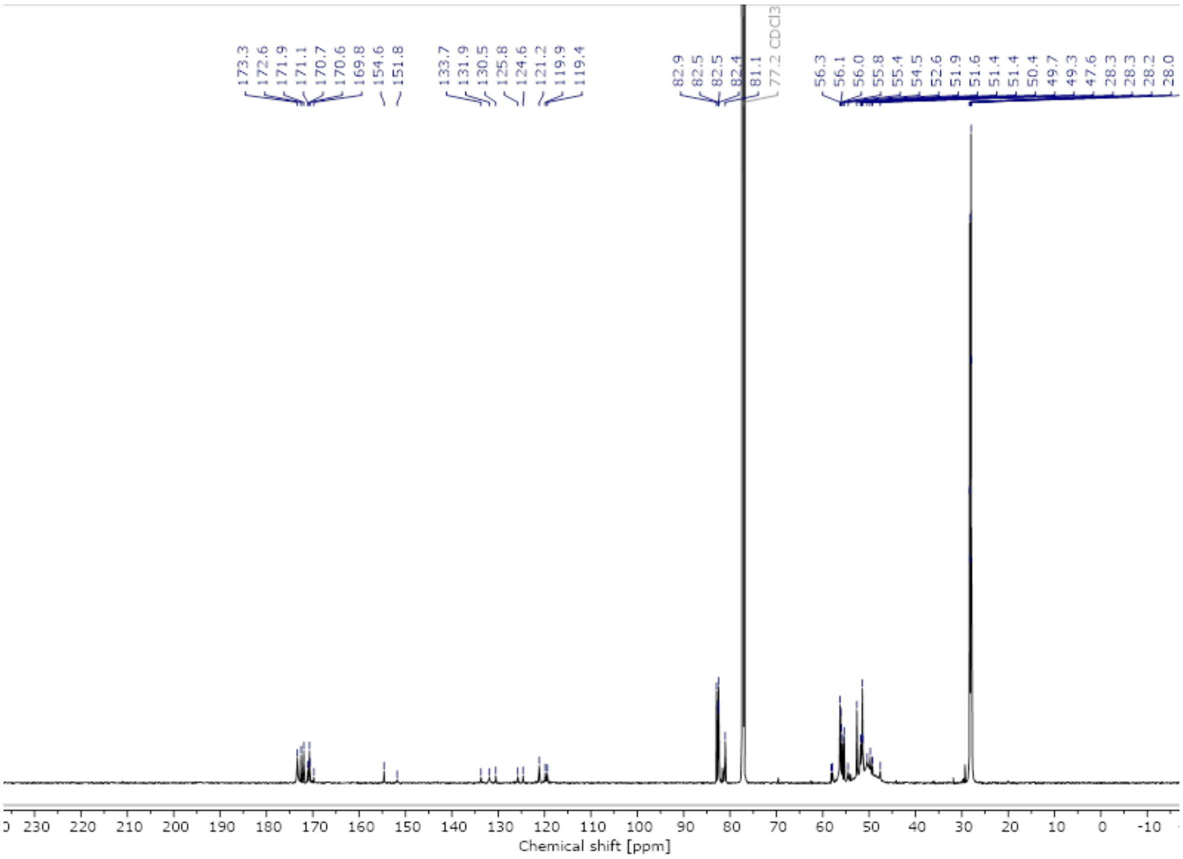

*Figure S81: ^{13}C NMR spectrum of **3e** (151 MHz, $CDCl_3$).*

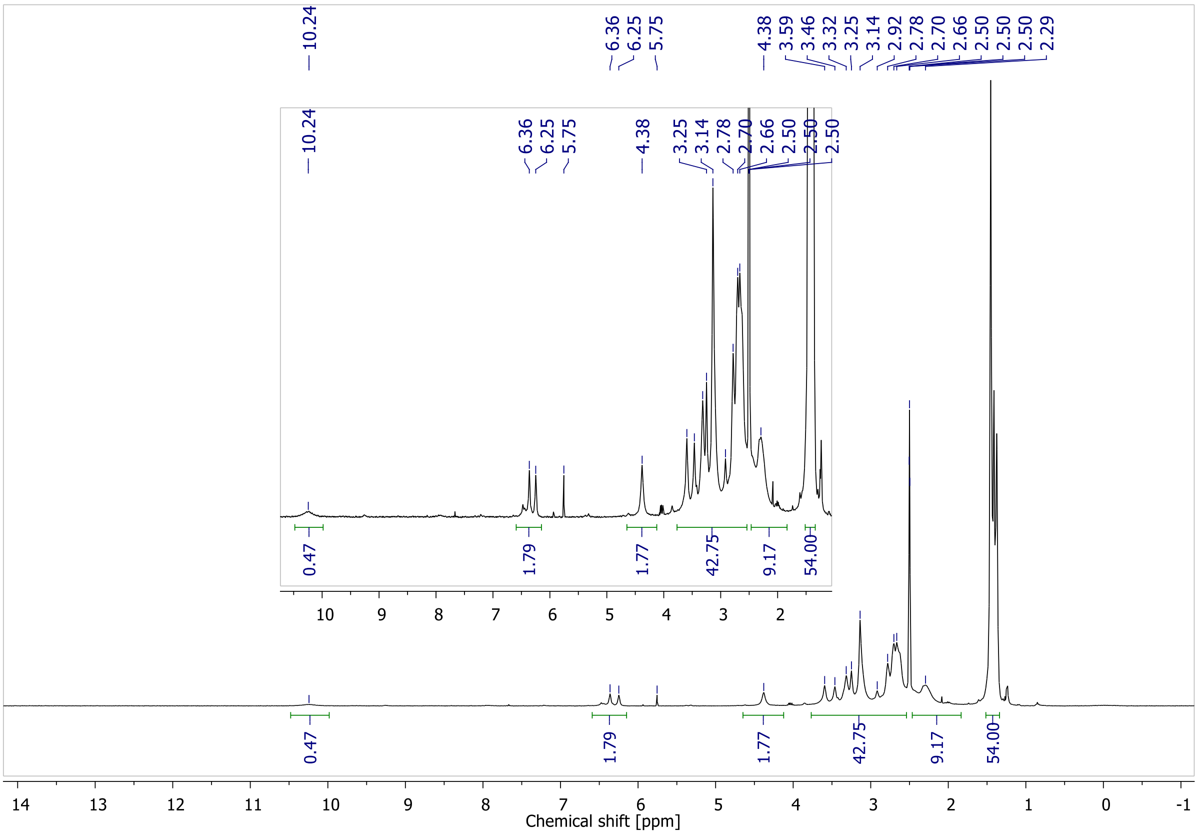

*Figure S82: ^{1}H NMR spectrum of **4e** (400 MHz, DMSO-d6)*

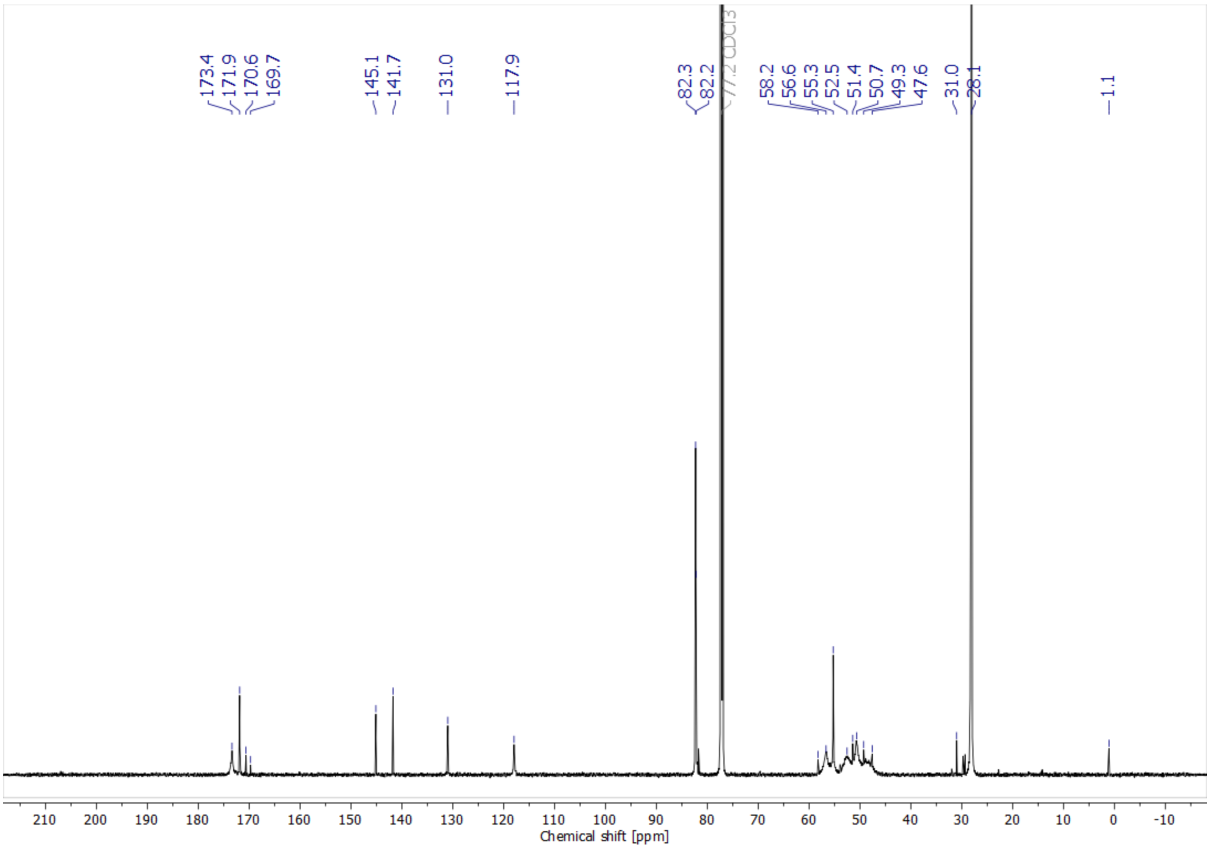

*Figure S83: ^{13}C NMR spectrum of **4e** (151 MHz, DMSO-d6)*

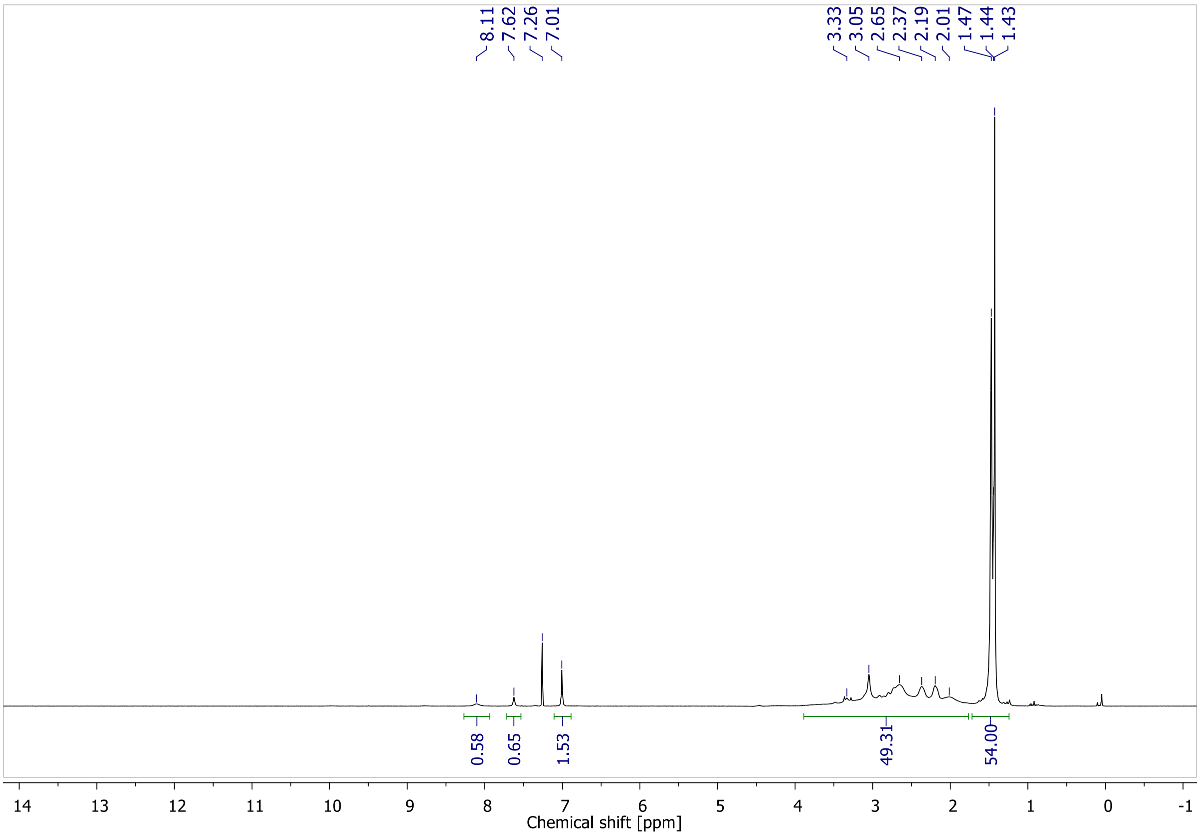

*Figure S84 ^{1}H NMR spectrum of **5e** (400 MHz, $CDCl_3$)*

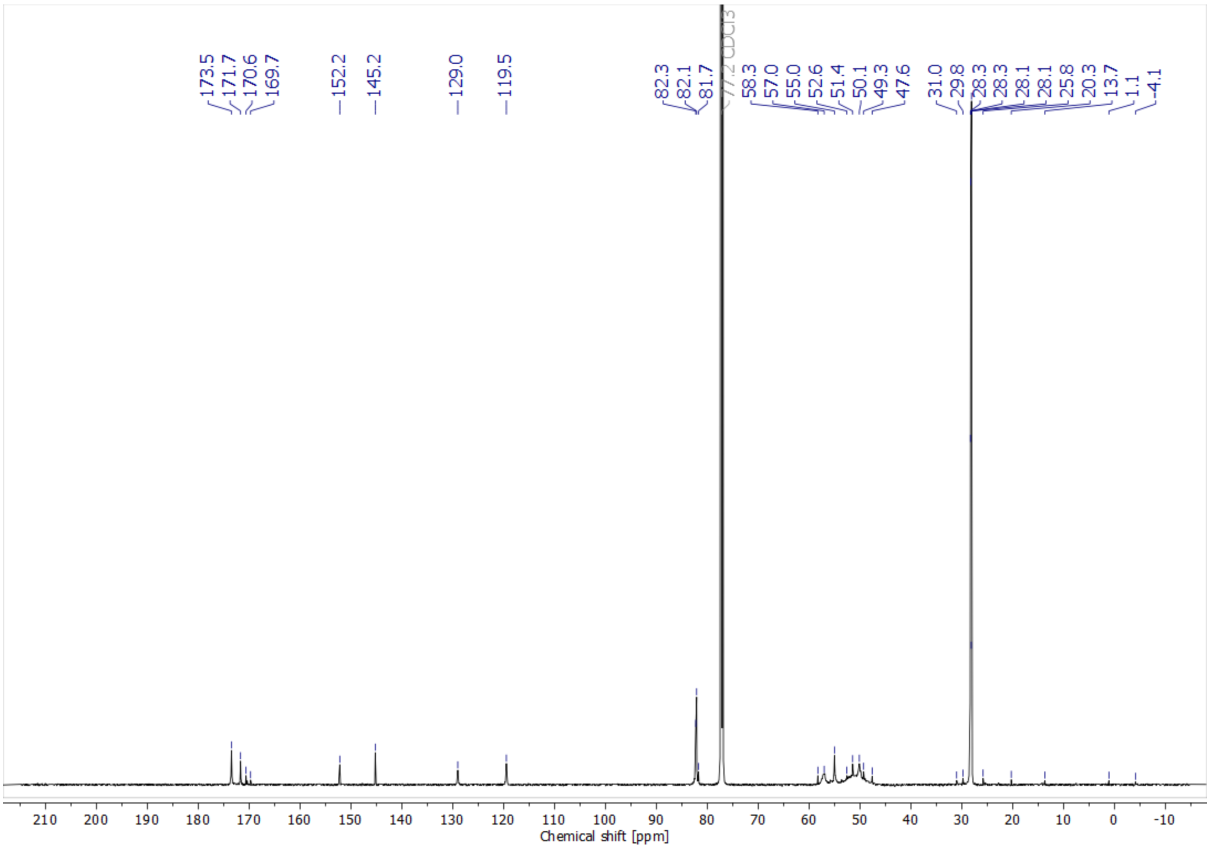

*Figure S85: ^{13}C NMR spectrum of **5e** (151 MHz, $CDCl_3$).*

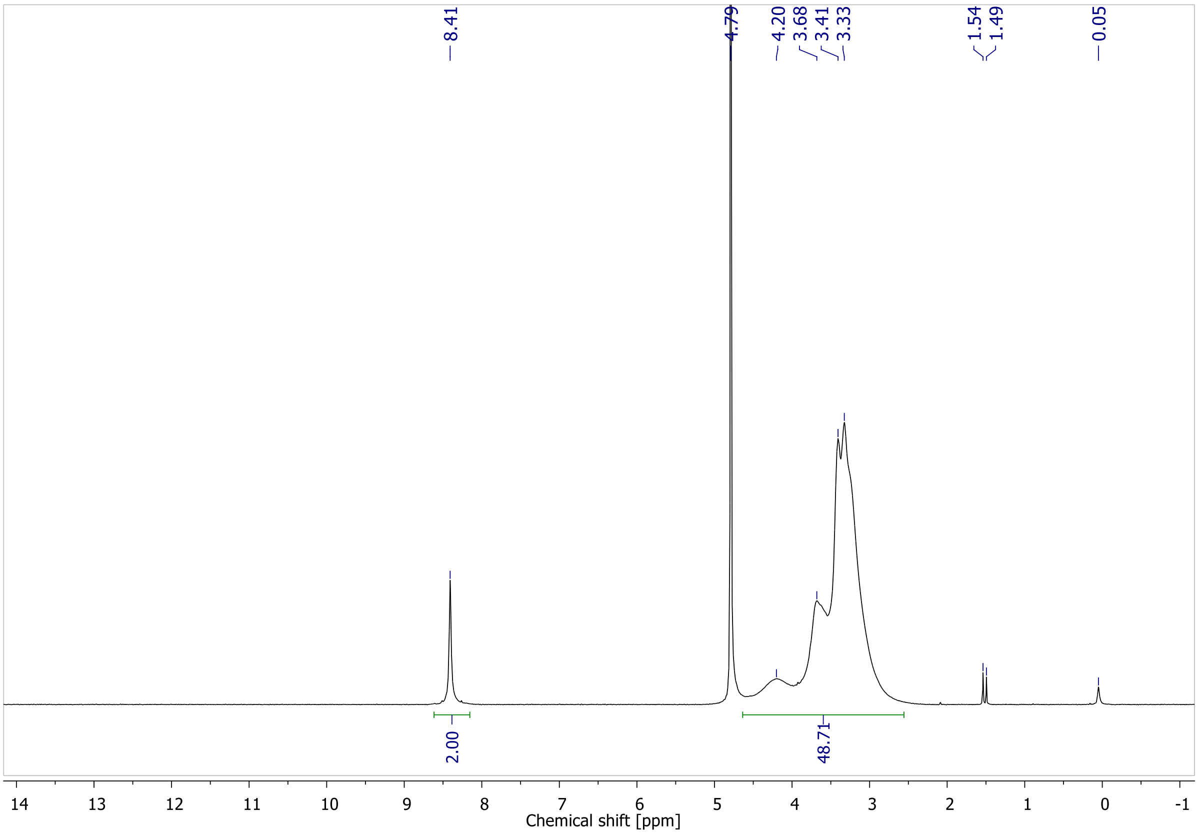

*Figure S86: ¹H NMR spectrum of **1f** (400 MHz, D_2O)*

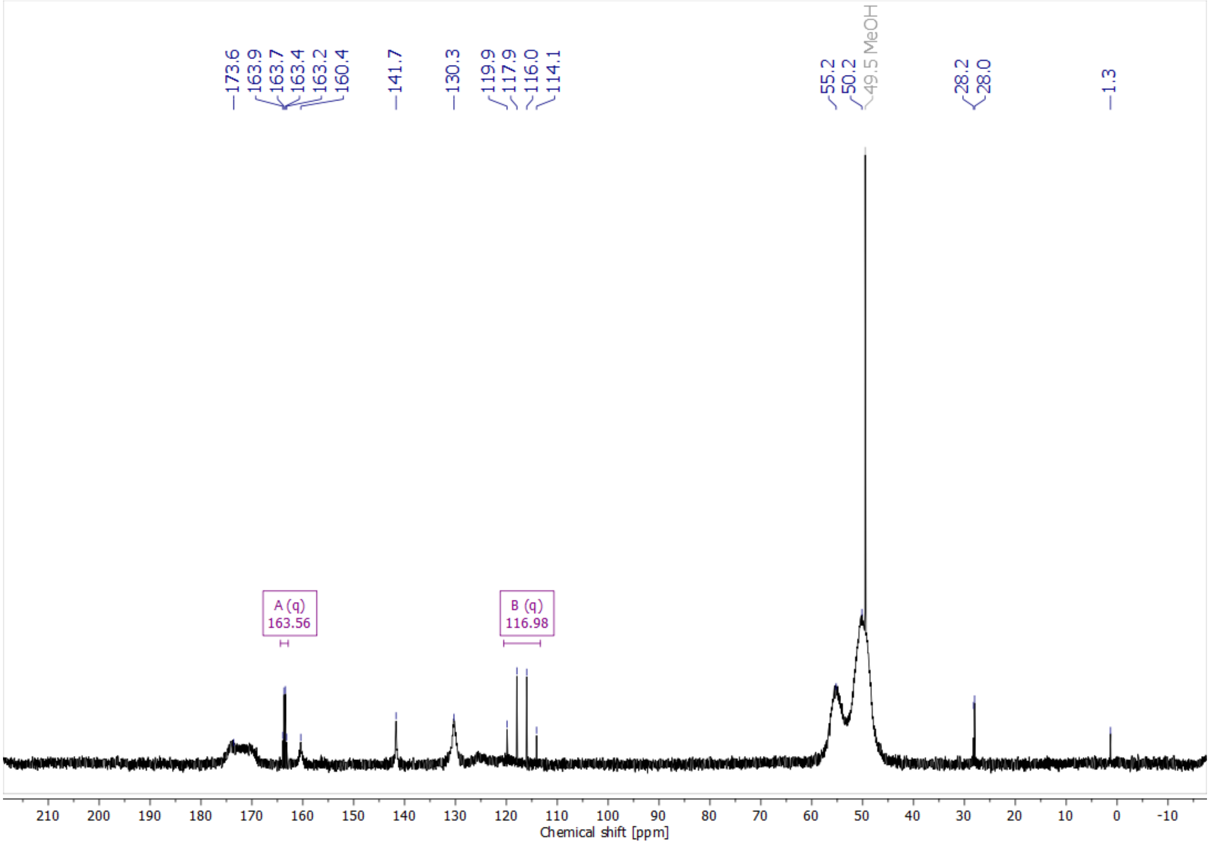

*Figure S87: ¹³C NMR spectrum of **1f** (151 MHz, D_2O)*

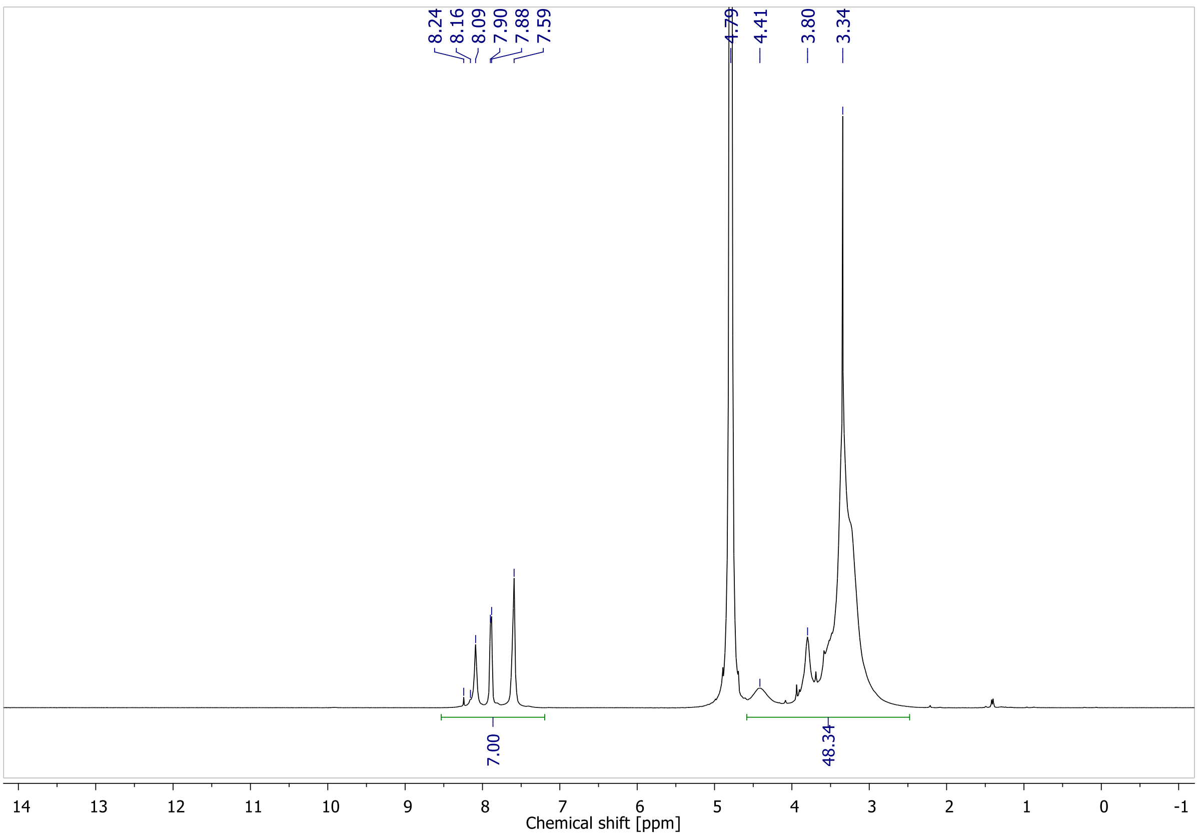

*Figure S88: ¹H NMR spectrum of **2f** (400 MHz, D_2O)*

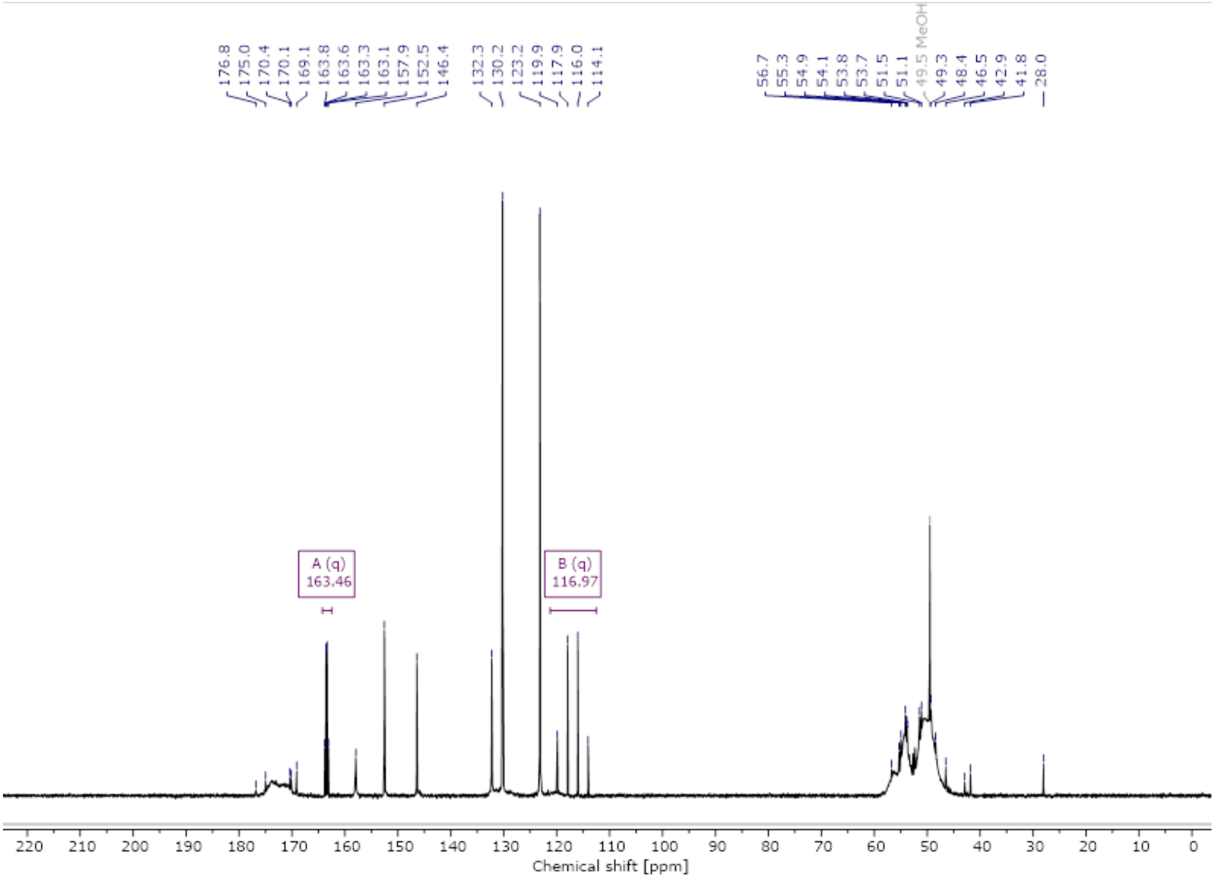

*Figure S89: ¹³C NMR spectrum of **2f** (151 MHz, D_2O).*

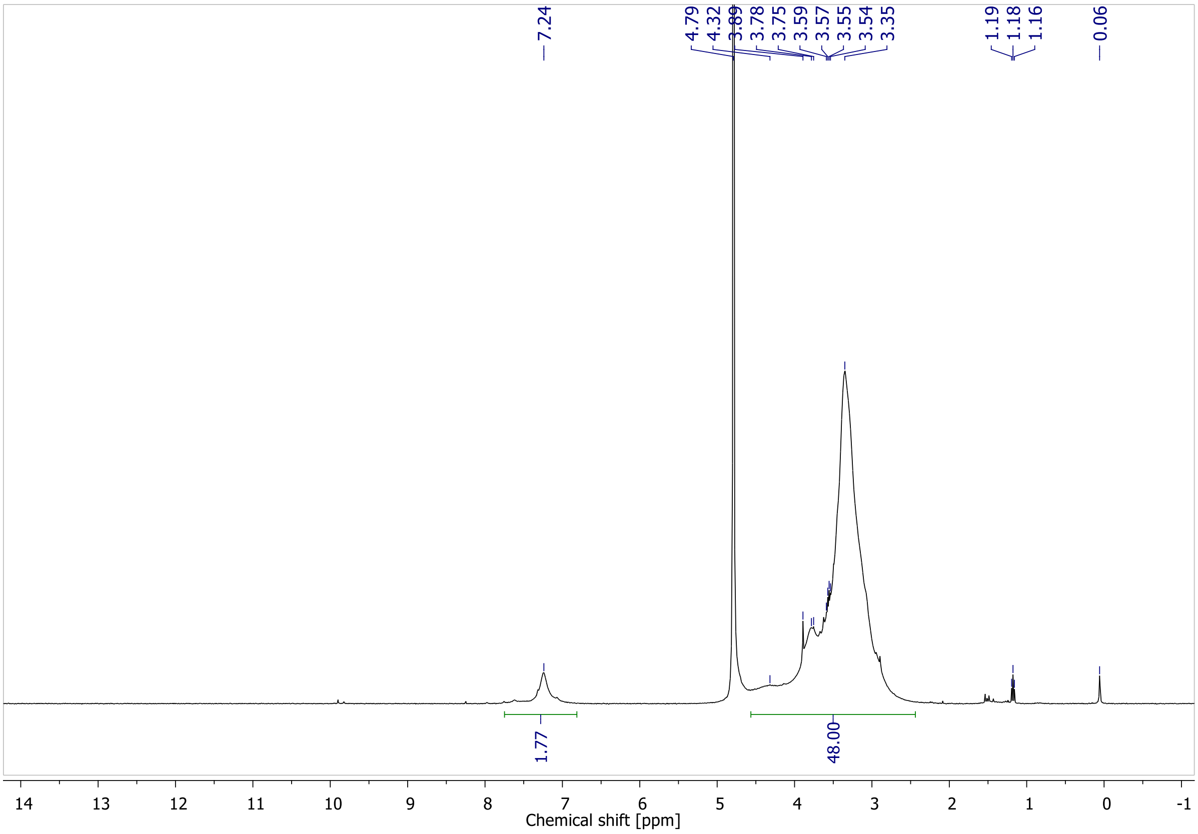

*Figure S90: ^{1}H NMR spectrum of **3f** (400 MHz, D_2O)*

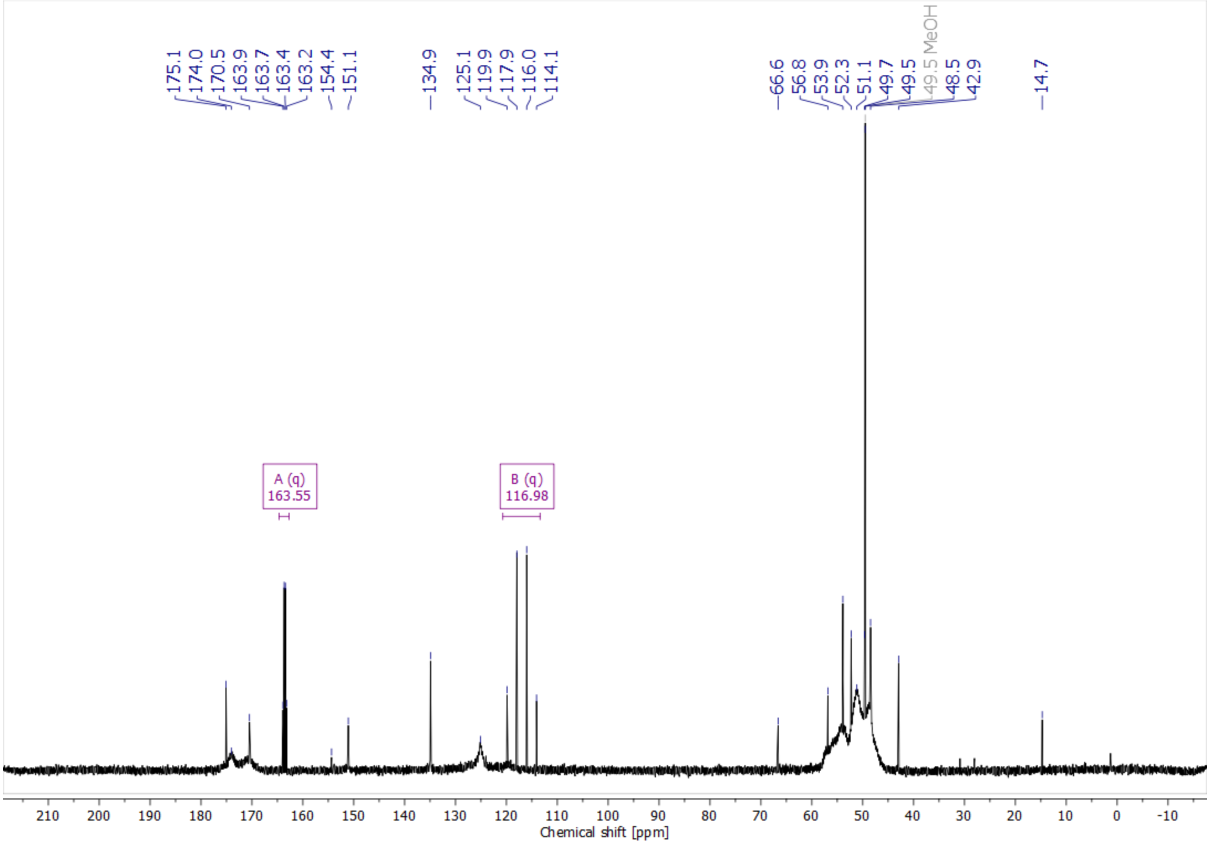

*Figure S91: ^{13}C NMR spectrum of **3f** (151 MHz, D_2O).*

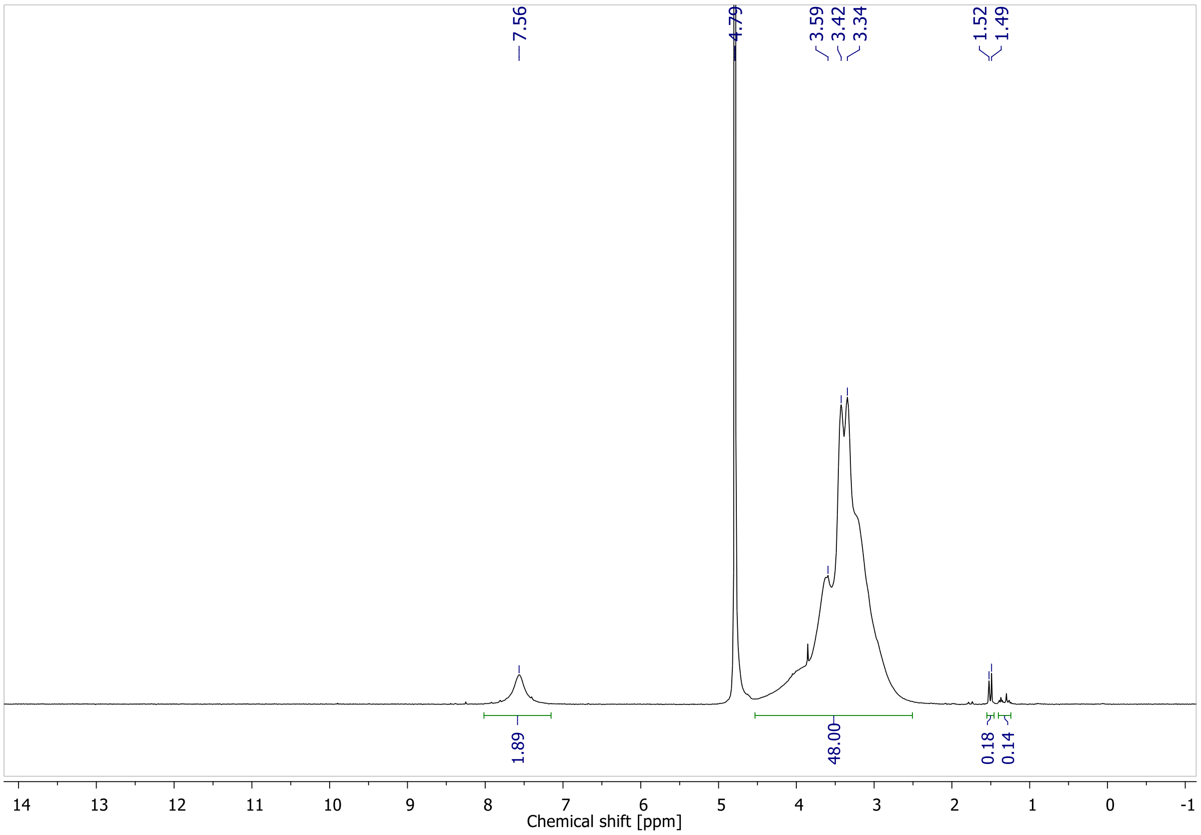

*Figure S92: ^{1}H NMR spectrum of **4f** (400 MHz, D_2O)*

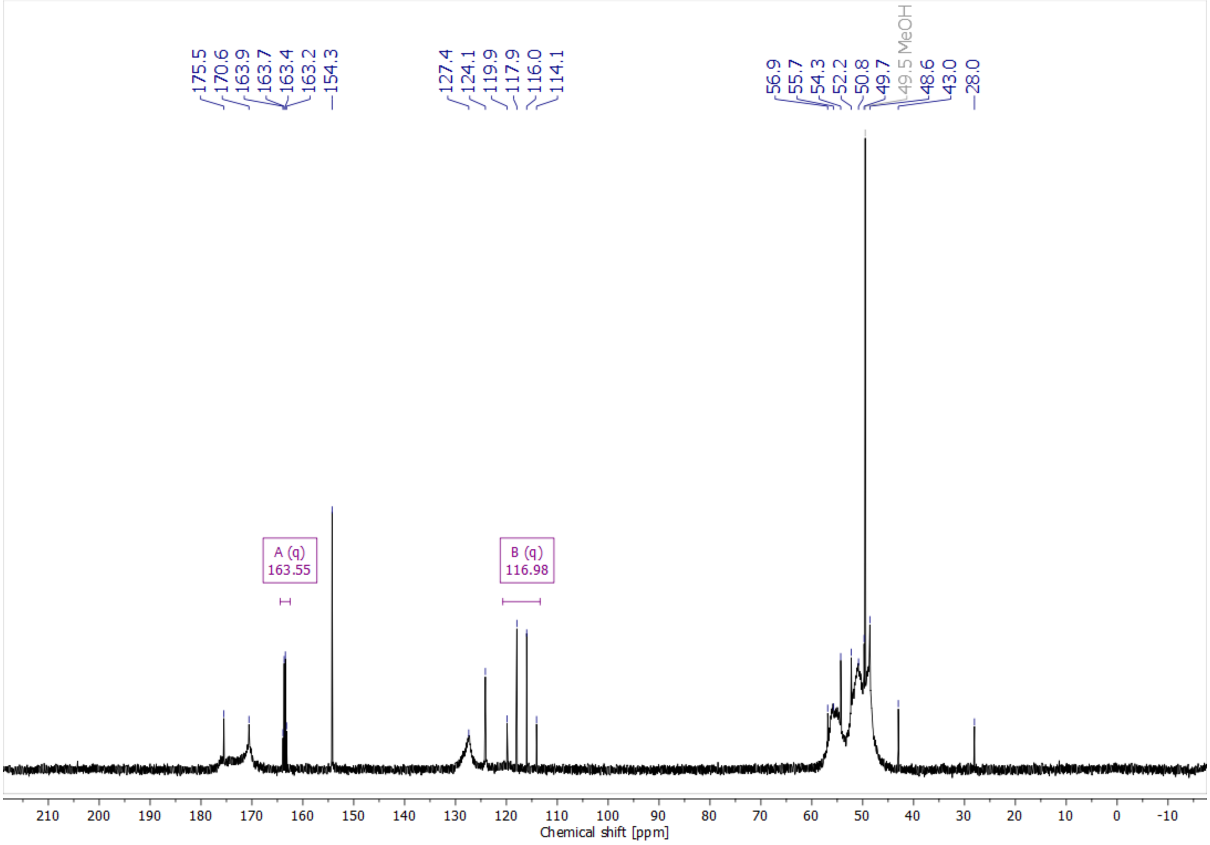

*Figure S93: ^{13}C NMR spectrum of **4f** (151 MHz, D_2O)*

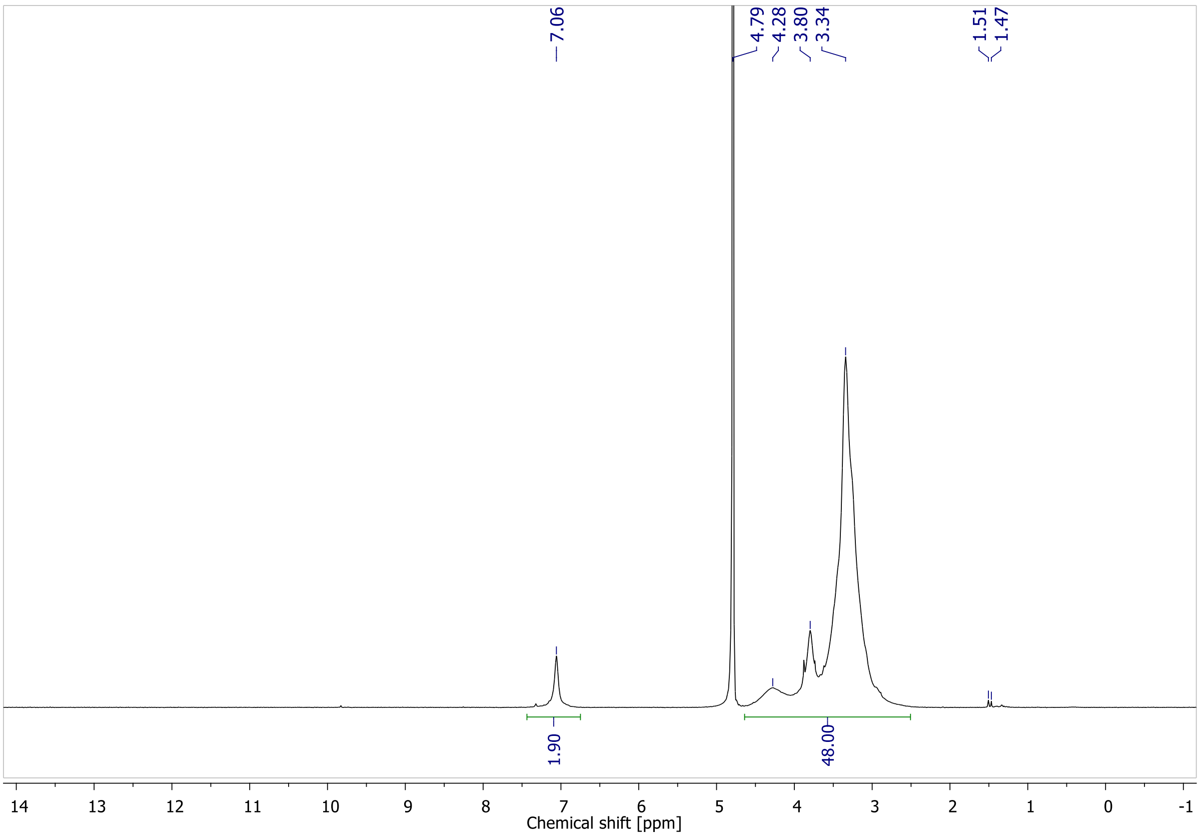

*Figure S94: ^{1}H NMR spectrum of **5f** (400 MHz, D_2O)*

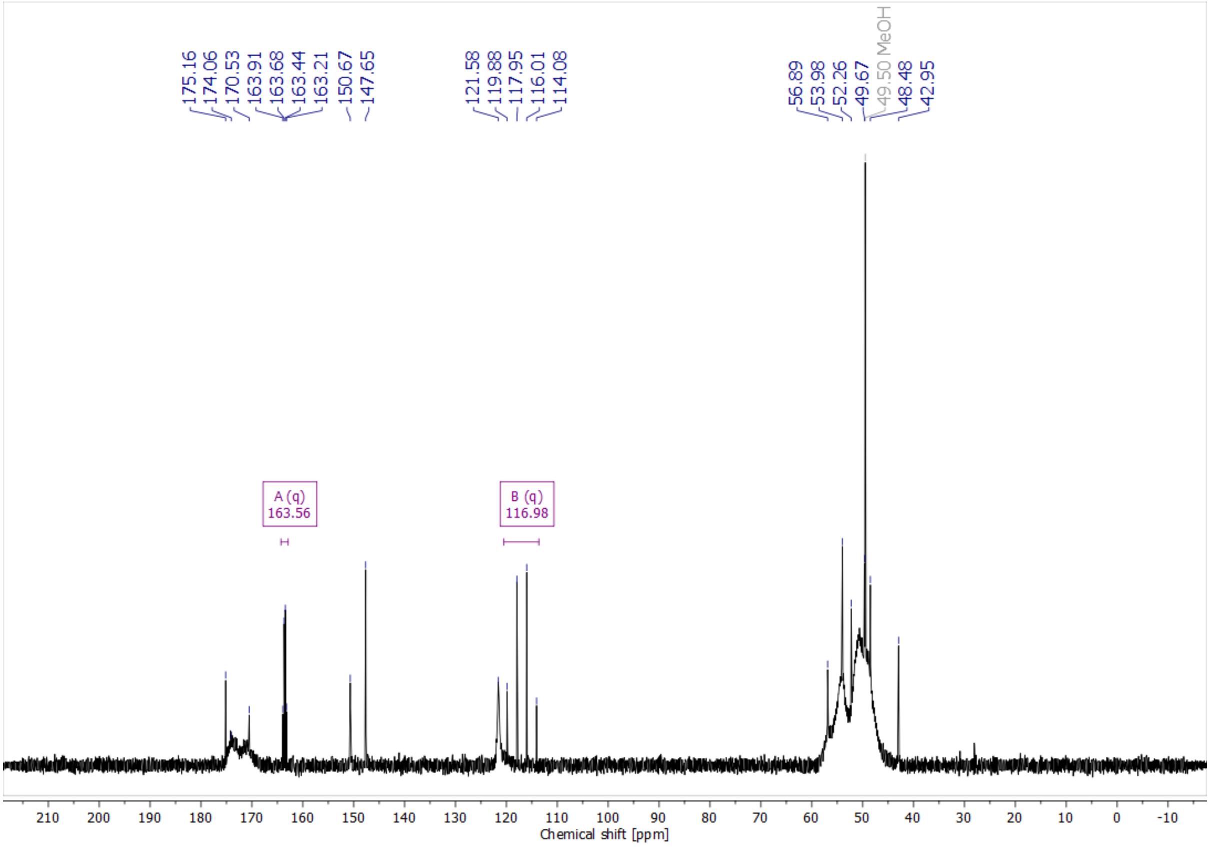

*Figure S95: ^{13}C NMR spectrum of **5f** (151 MHz, D_2O)*

References

[1] P. K. Glasoe, F. A. Long, *J Phys Chem-Us* **1960**, *64*, 188-190.

[2] A. Krezel, W. Bal, *J Inorg Biochem* **2004**, *98*, 161-166.

[3] D. Choi, N. Shiga, R. Franzén, T. Nemoto, *Eur J Org Chem* **2018**, *2018*, 1785-1788.

[4] A. Mariani, A. Bartoli, M. Atwal, K. C. Lee, C. A. Austin, R. Rodriguez, *Journal of Medicinal Chemistry* **2015**, *58*, 4851-4856.

[5] A. Veldhuizen, J., Edwin, H. Vaillancourt, Frédéric, J. Whiting, Cheryl, M.-Y. Hsiao, Marvin, G. Gingras, Y. Xiao, M. Tanguay, Robert, J. Boukouvalas, D. Eltis, Lindsay, *Biochemical Journal* **2005**, *386*, 305-314.

[6] O. A. Blackburn, M. Tropiano, L. S. Natrajan, A. M. Kenwright, S. Faulkner, *Chem Commun* **2016**, *52*, 6111-6114.

[7] P. Rodriguez-Maciá, A. Dutta, W. Lubitz, W. J. Shaw, O. Rüdiger, *Angewandte Chemie International Edition* **2015**, *54*, 12303-12307.

[8] Sokolova, D., Lurshay, T.C., Rowbotham, J.S., Stonadge, G., Reeve H.A., Cleery S.E., Sudmeier T., *Nat. Commun.,* **2024**, 15, 7297.
